# Atrial Fibrillation In Patients With Stroke Attributed to Large- or Small-Vessel Disease: 3-Year Results From the STROKE AF Randomized Clinical Trial

**DOI:** 10.1001/jamaneurol.2023.3931

**Published:** 2023-10-30

**Authors:** Richard A. Bernstein, Hooman Kamel, Christopher B. Granger, Jonathan P. Piccini, Jeffrey M. Katz, Pramod P. Sethi, Erika Pouliot, Noreli Franco, Paul D. Ziegler, Lee H. Schwamm

**Affiliations:** 1 Davee Department of Neurology, Feinberg School of Medicine of Northwestern University, Chicago, Illinois; 2 Department of Neurology, Weill Cornell Medicine, New York, New York; 3 Duke Clinical Research Institute, Duke University Medical Center, Durham, North Carolina; 4 Department of Neurology and Radiology, North Shore University Hospital, Manhasset, New York; 5 Guilford Neurology Associates, Moses H. Cone Hospital, Greensboro, North Carolina; 6 Cardiac Rhythm Management, Clinical Department, Medtronic, Minneapolis, Minnesota; 7 Cardiac Rhythm Management, Research Department, Medtronic, Minneapolis, Minnesota; 8 Department of Digital Strategy and Transformation, Yale School of Medicine, New Haven, Connecticut; 9 Deputy Editor, *JAMA Neurology*

## Abstract

**Importance:**

The STROKE AF study found that in patients with prior ischemic stroke attributed to large-artery atherosclerotic disease (LAD) or small-vessel occlusive disease (SVD), 12% developed AF over 1 year when monitored with an insertable cardiac monitor (ICM). The occurrence over subsequent years is unknown.

**Objectives:**

To compare the rates of AF detection through 3 years of follow-up between an ICM vs site-specific usual care in patients with prior ischemic stroke attributed to LAD or SVD.

**Design, Setting, and Participants:**

This multicenter, randomized (1:1) clinical trial took place at 33 sites in the US with enrollment between April 2016 and July 2019 and 3-year follow-up through July 2022. Eligible patients were aged 60 years or older, or aged 50 to 59 years with at least 1 additional stroke risk factor and had an index ischemic stroke attributed to LAD or SVD within 10 days prior to ICM insertion. Of the 496 patients enrolled, 492 were randomized and 4 were excluded.

**Interventions:**

ICM monitoring vs site-specific usual care.

**Main Outcomes and Measures:**

The prespecified long-term outcome of the trial was AF detection through study follow-up (up to 3 years). AF was defined as an episode lasting more than 30 seconds, adjudicated by an expert committee.

**Results:**

In total, 492 patients were randomized and included in the analyses (median [IQR] age, 66 [60-74] years; 307 men [62.4%] and 185 women [37.6%]), of whom 314 completed 3-year follow-up (63.8%). The incidence rate of AF at 3 years was 21.7% (46 patients) in the ICM group vs 2.4% (5 patients) in the control group (hazard ratio, 10.0; 95% CI, 4.0-25.2; *P* < .001).

**Conclusions and Relevance:**

Patients with ischemic stroke attributed to LAD or SVD face an increasing risk of AF over time and most of the AF occurrences are not reliably detected by standard medical monitoring methods. One year of negative monitoring should not reassure clinicians that patients who have experienced stroke will not develop AF over the next 2 years.

**Trial Registration:**

ClinicalTrials.gov Identifier: NCT02700945

## Key Points

**Question:**

Do insertable cardiac monitors (ICMs) detect more atrial fibrillation (AF) than usual care in patients with recent ischemic stroke attributed to large-artery atherosclerotic disease (LAD) or small-vessel occlusive disease (SVD)?

**Findings:**

The STROKE AF trial randomized 492 patients with stroke attributed to LAD or SVD to ICM or usual care. AF rates at 3 years were 21.7% in the ICM group and 2.4% in the control group, a statistically significant difference.

**Meaning:**

Patients with ischemic stroke attributed to LAD or SVD face an increasing risk of AF over time and most of the AF occurrences are not reliably detected by standard medical monitoring methods.

## Teaser

This study compares the rates of atrial fibrillation detection through 3 years of follow-up between an insertable cardiac monitor vs site-specific usual care, in patients with prior ischemic stroke attributed to large-artery disease or small-vessel disease.

## Introduction

Atrial fibrillation (AF) quintuples the risk of ischemic stroke in patients with other stroke risk factors.^1^ Prior ischemic stroke is a potent risk factor, and in common risk-stratification schemes, it increases the predicted risk of future stroke regardless of the putative mechanism of the prior stroke (eg, cardioembolic, small vessel, large vessel, or of unknown cause). In patients with AF and more than 1 stroke risk factor, long-term treatment with oral anticoagulation (OAC) for stroke prevention is among the most effective guideline-recommended therapies in medicine. When AF is detected after ischemic stroke, most patients begin OAC or receive other specific stroke-preventive therapy (eg, left atrial appendage occlusion), so detection carries significant treatment implications. AF often starts with infrequent, asymptomatic, and brief episodes that may elude detection with standard poststroke monitoring techniques (recently described as AF discovered after stroke [AFDAS]), but that can be detected with greater frequency using insertable cardiac monitors (ICM) and other more intensive strategies.^2,3^ Use of ICM has been shown to result in a higher rate of detection of AF compared with standard monitoring techniques in patients with cryptogenic stroke and an appearance suggesting embolic stroke of undetermined source, and more recently, with large-artery atherosclerotic (LAD) or small-vessel occlusive disease (SVD).^4,5,6^

In patients with embolic stroke of undetermined source, AF detection is presumed to identify the mechanism of the index stroke and justify the use of OAC. However, AFDAS is detected by ICM with similar frequency after stroke attributed to LAD or SVD.^4^ AFDAS may not be the mechanism of the preceding stroke, but it raises the risk of future stroke^2^ and should prompt consideration of the risks and benefits of OAC vs antiplatelet therapy. Therefore, AFDAS in patients with stroke presumed to be due to LAD or SVD has prognostic and treatment implications.

The Stroke of Known Cause and Underlying Atrial Fibrillation (STROKE AF) study measured the rate of AFDAS in patients with stroke attributed to LAD or SVD and multiple vascular risk factors. It demonstrated that ICM was superior to usual care (12.5% vs 1.8% at 1 year)^4^ and that patients with heart failure and/or left atrial enlargement were at significantly higher risk (23.4% vs 5.0% for patients with neither [hazard ratio [HR], 5.1; 95% CI, 2.0-12.8; *P* < .001]).^7^ It remains unknown for how long after index stroke the detection of AFDAS continues to increase and whether the superiority of ICM over standard monitoring persists beyond 1 year in this patient population. Here, we present the results of the STROKE AF 3-year poststroke follow-up, rate of AFDAS, assessment of high-risk subgroups, AF burden, and clinical implications.

## Methods

STROKE AF was a multicenter, randomized (1:1), parallel-group clinical trial conducted at 33 sites in the US that enrolled 496 patients from April 2016 to July 2019 with follow-up through July 2022. The study design, patient characteristics, settings, randomization, sample size calculations, and primary outcome have been previously described^4,8^ (protocol and statistical analysis plan, Supplement 3; protocol modifications after study initiation, eTable 1 in Supplement 1). Briefly, patients with an ischemic stroke classified as presumed due to LAD or SVD by TOAST criteria^9^ were randomized to site-specific usual care or received an ICM (Reveal LINQ; Medtronic) within 10 days of the qualifying stroke. Patients were 60 years or older, or 50 to 59 years with at least 1 stroke risk factor (congestive heart failure, hypertension, diabetes, ischemic stroke more than 90 days before the index stroke, or other ischemic vascular disease). The study was conducted in compliance with international ethical and scientific quality standards and the principles of the Declaration of Helsinki. All patients provided written informed consent and the study was approved by all relevant institutional review boards.

AF was defined as an episode of irregular heart rhythm without detectable P waves lasting more than 30 seconds, as adjudicated by a Clinical Events Committee. However, due to the ICM’s automatic detection algorithms, all detected episodes in the ICM group were at least 2 minutes in duration. AF burden is the daily cumulative time spent in AF. This metric was collected automatically by the device as a function of the Cardiac Compass Trends software and was not clinically adjudicated since the AF algorithm has previously been shown to quantify AF burden with 98.9% accuracy.^10^ Recurrent stroke was defined as any hemorrhagic or ischemic event with rapid onset of a focal or global neurologic deficit or other neurologic signs/symptoms consistent with stroke. Transient ischemic attack was defined as any new focal neurologic deficit with rapid symptom resolution (usually 1 to 2 hours), always within 24 hours, without tissue injury (based on neuroimaging).

The long-term outcome is a prespecified secondary outcome of the trial. We compared AF detection through study follow-up (up to 3 years) between randomized groups. One patient for whom 12-month detection data were missing at time of initial publication^4^ is now included in the 12-month rate. A subgroup analysis comparing patients according with index stroke subtype was also performed. Other prespecified analyses were to compare secondary stroke prevention interventions and rates of recurrent stroke between study groups, describe the proportion of asymptomatic AF episodes in the ICM group (AF detected without a corresponding symptomatic event trigger on the patient activation recorder), characterize AF duration and burden, and assess clinical, electrocardiographic, and echocardiographic predictors of AF.

### Statistical Analysis

**Figure 1.**
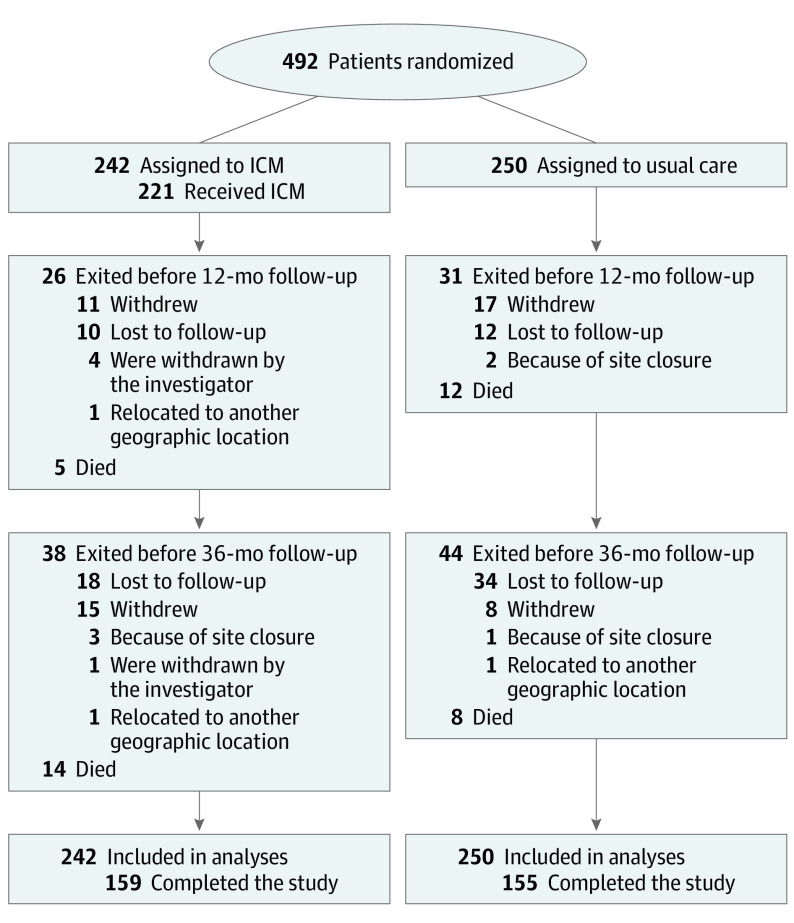
Patient Flow in STROKE AF Trial ICM indicates insertable cardiac monitor.

Statistical analyses were performed using SAS version 9.4 (SAS Institute) and R version 4.2.1 (R Foundation for Statistical Computing). The analysis set consisted of all randomized patients (Figure 1). Patients were analyzed according to their randomization group. Kaplan-Meier incidence estimates are reported for each group, as well as an HR estimate for the effect with corresponding 2-sided 95% CI. The Cox models analyzed time to first AF episode through the duration of the study. Prespecified subgroup analyses of patient by stroke subtype (LAD and SVD) were performed. Since there is potential for type I error due to multiple comparisons, findings for analyses of post hoc end points should be interpreted as exploratory. Statistical significance was set at a 2-sided *P* value of .05 for all analyses. For the AF predictors analysis, all patients with ICM assigned to the ICM group were included. Left atrial enlargement (LAE) was derived from left atrial volume, and if this was missing, then by left atrial diameter. Predictors of AF detection were selected for a multivariable model based on *P* < .10 in univariate analysis, using patients who had all available values of each predictor. Body mass index (BMI; calculated as weight in kilograms divided by height in meters squared) (30 or less vs more than 30) and QRS duration (120 milliseconds or less vs more than 120 milliseconds) were dichotomized into binary variables to allow for the creation of a composite predictor.

## Results

Of the 496 patients enrolled in the trial, 492 were randomized (Figure 1; 3 did not meet inclusion criteria and 1 withdrew consent). The median (IQR) age of the study population was 66 (60-74) years, 185 were women (37.6%), and the median (IQR) CHA_2_DS_2_-VASc score was 5 (4-6). Baseline characteristics for the entire cohort and for patients completing the 3-year follow-up are shown in eTable 2 in Supplement 1. The mean (SD) follow-up duration was 29.4 (12.5) months. There were 21 crossovers during follow-up: 8 assigned to ICM never received it, 5 assigned to ICM crossed to usual care more than 1 year after ICM insertion, and 8 assigned to usual care received an ICM during follow-up.

### AF Detection by 3 Years

**Figure 2.**
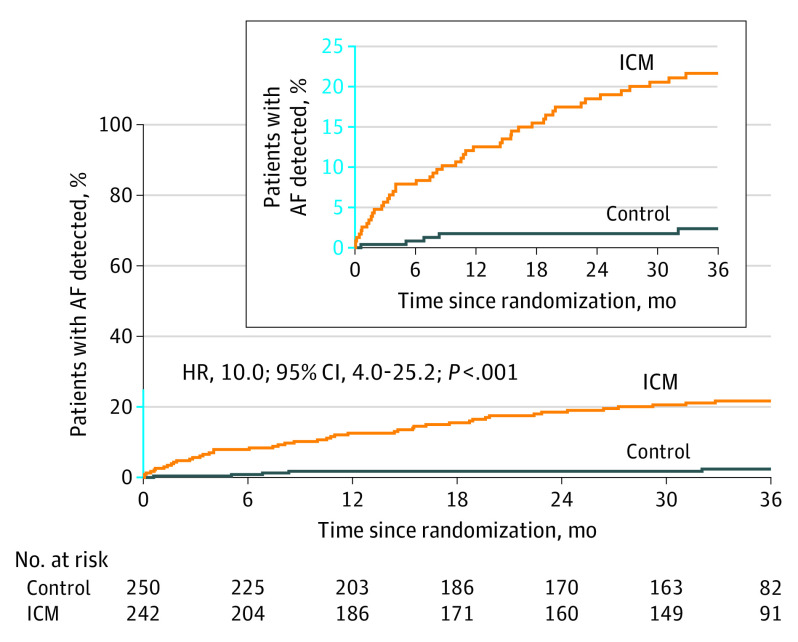
Time to First Detection of Atrial Fibrillation (AF) Through 3 Years in the STROKE AF Trial The median (IQR) observation time was 35.2 (18.6-36.4) months for the control group and 35.8 (29.5-36.9) months for the insertable cardiac monitor (ICM) group. HR indicates hazard ratio.

The incidence rate of AF at 3 years was 21.7% (n = 46) in the ICM group vs 2.4% (n = 5) in the control group (HR, 10.0; 95% CI, 4.0-25.2; *P* < .001) (Figure 2). AF detection levels in the ICM group increased throughout the study: 2.6% (n = 6) at 1 month, 7.9% (n = 18) at 6 months, 12.5% (n = 28) at 12 months, and 18.5% (n = 40) at 2 years. The median (IQR) time from randomization to AF detection was 9.3 (2.7-18.7) months and 6.8 (5.1-8.4) months for those with detection in the ICM (n = 46) and control groups (n = 5), respectively. When censoring data beyond 30 days from enrollment, 40 of 46 patients with AF and at 3 years in the ICM group would not have had their AF detected (87%).

A total of 200 heart rhythm recordings were performed in the control group: 162 electrocardiograms in 93 patients, 34 Holter monitor/event recorders in 30 patients, and 4 mobile cardiac telemetry devices in 4 patients. One of the 5 patients with AF detected in the control group had been assigned to receive usual care (20%) but crossed over and had AF detected via the ICM.

In the subgroup analysis of the AFDAS rate at 3 years according to index stroke subtype (LAD [n = 284 (57.3%)] and SVD [n = 208 (42.3%)]), AF incidence was significantly higher in ICM vs control regardless of stroke subtype: LAD (25 [20.4%] vs 4 [3.5%]; HR, 6.4; 95% CI, 2.2-18.3; *P* < .001 and SVD (21 [23.4%] vs 1 [1.0%]; HR, 24.7; 95% CI, 3.3-183.6; *P* < .001) (eFigure 1 in Supplement 1). In the ICM group, the rates of AFDAS among patients with index stroke subtypes classified as LAD vs SVD were similar (log-rank *P* value = .55).

### ICM-Detected AF

**Figure 3.**
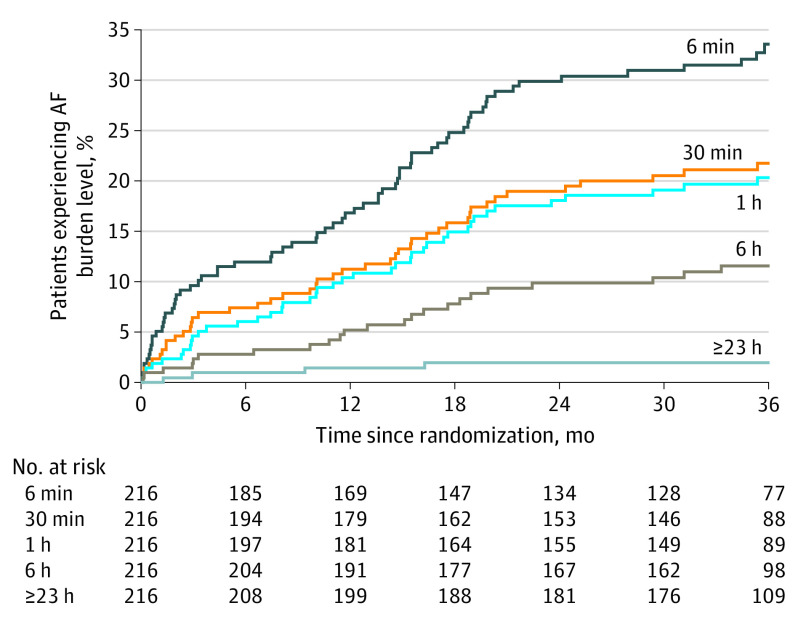
Time to Onset of 5 Thresholds of Daily Atrial Fibrillation (AF) Burden in Participants in STROKE AF Trial Only patients from the insertable cardiac monitor (ICM) group were included in the analysis. At 36 months, the incidence of AF burden was 33.9%, 22.0%, 20.5%, 11.6%, and 1.9% for 6 or more minutes, 30 or more minutes, 1 or more hours, 6 or more hours, and 23 or more hours of AF in a day, respectively. The median (IQR) observation time was 35.8 (29.5-36.9) months for the ICM group.

In the ICM group, AF was asymptomatic (ie, no event trigger recorded by the patient) in 88% of all episodes recorded by the device. The median (IQR) duration of the longest single AF episode per patient was 176 (26-732) minutes and 31 of 46 patients with AF had at least 1 episode lasting more than 1 hour (67.4%). The time to onset of AF burden at various thresholds for the ICM group is shown in Figure 3. By 3 years, 20.5% had at least 1 day where 1 hour or more of AF burden was detected.

In a post hoc analysis, progression of AF burden was evaluated in the 46 participants from the ICM group who had AF detected and confirmed through adjudication. Progression was measured from the first day with at least 6 minutes of AF burden to the day with the longest amount of AF burden. Of the 42 participants who had at least 6 minutes of AF burden, 28 of them had AF burden progression (66%). The median (IQR) increase in AF burden from first to longest instance was 9.8 (4.0-15.3) hours.

### Secondary Prevention

Through study follow-up, 2 patients in the ICM group received left-atrial appendage occlusion and no AF ablations were performed. OAC therapy was prescribed to patients with and without AF more often in ICM vs control (24.4% vs 8.0%; unadjusted odds ratio, 3.7; 95% CI, 2.2-6.4; *P* < .001); direct OAC was the predominant drug class (233 of 261 OAC prescriptions [89%]). The incidence rate of first recurrent ischemic and/or hemorrhagic stroke was similar in ICM vs control (17.0% vs 14.1%; HR, 1.1; 95% CI, 0.7-1.8; *P* = .71) (eFigure 2 in Supplement 1).

In the ICM group, 46 patients had AF detected and OAC was prescribed in 35 patients (76%). Of the 34 patients with recurrent stroke, 3 had AF detected prior to the recurrent stroke (8.8%) and of those 3 only 1 was taking OAC therapy at the time of the recurrent stroke. Use of OAC in the absence of AF was observed in 24 of 196 patients (12%).

In the control group, 5 patients had AF detected and OAC was newly prescribed in 3 patients and continued in 1 patient. None of the 31 patients with a recurrent stroke had AF detected, although occult AFDAS cannot be excluded, given the absence of continuous monitoring. Use of OAC in the absence of AF was observed in 16 of 245 patients (6.5%). None of the 20 patients on OAC (4 with AF and 16 without) had a stroke after initiation.

eTable 3 in Supplement 1 shows the classification of first recurrent stroke stratified by index stroke and randomization group. The incidence rate of recurrent stroke in patients with an index LAD was not statistically different than that for SVD (17.3% vs 13.4%; HR, 1.44; 95% CI, 0.86-2.39; *P* = .16) (eFigure 3 in Supplement 1). The 3-year incidence of recurrent transient ischemic attack (5.4% vs 3.7%; HR, 1.48; 95% CI, 0.61-3.63; *P* = .38) or hemorrhagic stroke (2.3% vs 0.5%; HR, 3.83; 95% CI, 0.43-34.3; *P* = .23) was not different in the ICM vs control groups.

### Predictors of AF

**Figure 4.**
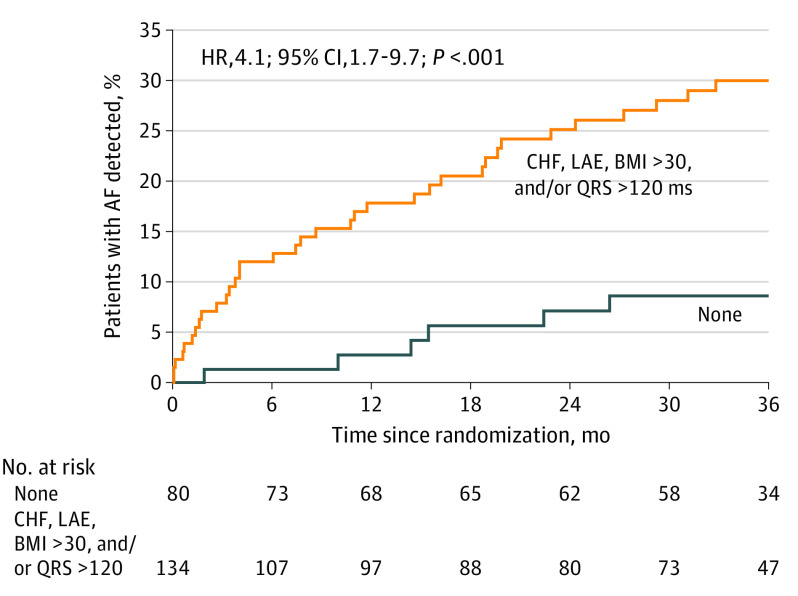
Time to First Detection of Atrial Fibrillation (AF) Through 3 Years Among Participants With Congestive Heart Failure (CHF), Left Atrial Enlargement (LAE), Body Mass Index (BMI) More Than 30, and/or QRS Duration More Than 120 Milliseconds Only patients from the insertable cardiac monitor group (ICM) group were included in the analysis. At 36 months, patients with CHF, LAE, BMI more than 30, and/or QRS more than 120 ms had an AF detection rate of 30.0% vs 8.6% in patients without any of those factors (hazard ratio [HR], 4.1; 95% CI, 1.7-9.7; *P* < .001). The median (IQR) observation time was 35.8 (29.5-36.9) months for the ICM group.

Univariate predictors of 3-year AF detection in the ICM group were older age, higher BMI, higher CHA_2_DS_2_-VASc score, congestive heart failure, higher diastolic blood pressure, hypertension, LAE, longer QRS duration, and kidney dysfunction (eTable 4 in Supplement 1). Only 1 left atrial measure, LAE, was included in the multivariable analysis due to collinearity. In multivariable analysis (n = 197) (Table), BMI, congestive heart failure, LAE, and QRS duration were independently associated with an increased likelihood of AF detection during 3 years of monitoring. Patients with at least 1 of the independent predictors in the multivariable model had a greater than 4-fold increase in AF detection by 3 years compared with those with no predictors (30.0% vs 8.6%; HR, 4.1; 95% CI, 1.7-9.7; *P* < .001) (Figure 4).

**Table.** Predictors of AF Through 3 Years in Participants of the STROKE AF Trial

Predictor	Unadjusted hazard ratio (95% CI)	*P* value	Adjusted hazard ratio (95% CI), n = 197	*P* value
Congestive heart failure	4.12 (1.47-11.52); n = 240	.01	4.31 (1.33-13.97)	.02
Left atrial enlargement	2.24 (1.21-4.16); n = 214	.01	2.10 (1.09-4.05)	.03
BMI	1.05 (1.00-1.09); n = 240	.04	1.07 (1.01-1.13)	.02
QRS duration	1.02 (1.00-1.03); n = 219	.01	1.02 (1.01-1.04)	.01
Hypertension	2.52 (0.90-7.02); n = 240	.08	2.19 (0.59-8.13)	.24
Age	1.04 (1.01-1.07); n = 240	.02	1.03 (0.98-1.08)	.25
CHA_2_DS_2_-VASc score	1.53 (1.22-1.92); n = 240	<.001	1.22 (0.84-1.78)	.29
Diastolic blood pressure	0.98 (0.96-1.00); n = 240	.09	1.01 (0.99-1.04)	.30
Kidney dysfunction	2.91 (1.30-6.50); n = 240	.01	1.32 (0.51-3.47)	.57

Abbreviations: BMI, body mass index; CHA2DS2-VASc, congestive heart failure, hypertension, age, diabetes, prior stroke or transient ischemic attack or thromboembolism, vascular disease, age, sex.

## Discussion

The 3-year results of the randomized STROKE AF trial showed the risk of AF detection after stroke attributed to LAD or SVD continues to rise beyond 1 year in patients monitored with an ICM, from 12.5% at 1 year to 21.7% at 3 years. In contrast, the detection rate in patients undergoing standard monitoring remained low at 2.4% despite the performance of numerous intermittent recordings. Similar to the findings at 1 year, risk factors for ICM detection of AFDAS include heart failure and left atrial enlargement, with QRS duration and BMI achieving significance as additional risk factors.^7^ Most AF episodes in the ICM group were asymptomatic and most patients with AF had at least 1 episode greater than an hour. AF burden increased over the course of monitoring in many patients with at least 6 minutes of AF detected.

Our findings have several clinical implications. First, AF is common in patients after stroke attributed to noncardioembolic mechanisms. Second, clinicians should not be reassured after 1 year of negative monitoring that the patient’s risk for developing AF will continue to remain low, as the risk continues to increase for at least 3 years after stroke. Third, this AF is largely not detected by patients themselves and negative intermittent episodic rhythm monitoring should not be reassuring. Fourth, patients with left atrial enlargement, heart failure, obesity, or prolonged QRS may constitute high-risk groups in which monitoring might be most cost-effective. Lastly, even if the initial episode of AF is short and of questionable clinical significance, most patients with AF will experience increasing burden over time with a likely corresponding increase in the risk of stroke.

In contrast to a similar study of continuous monitoring in patients with cryptogenic stroke,^6^ a substantial minority of patients with device-detected AF were not prescribed OAC. While the reasons for this were not documented by the investigators, it is possible that there is uncertainty among neurologists and other treating physicians about the clinical significance of AFDAS in patients with presumed noncardioembolic index strokes. We emphasize again that the goal of diagnostic testing after clinical practice should be to detect all risks for future stroke, not just those related to the presumed mechanism of the index stroke. Just as clinicians should not overlook treatment of diabetes or dyslipidemia in a patient with recent cardioembolic stroke, they should not overlook treatment of AF in a patient with recent lacunar stroke. AFDAS has been shown to increase the risk of both recurrent stroke and death,^2^ although the optimal thresholds of AF burden and risk factors for initiating anticoagulation remain unknown. Given the fact that in most patients who demonstrate AF occurrence, there is an increase in AF burden (and presumably related risk of stroke) over time, it may be that early so-called device-detected subclinical AF identifies AF at a time when the stroke risk may be relatively low. However, it is not known how long it is safe to wait before initiating OAC, so the risk of harm from exposing a patient to OAC prematurely must be weighed against the risk of harm from failing to treat with OAC prior to a disabling cardioembolic stroke. Given that most patients with AF will eventually be prescribed lifelong OAC, the additive risk of initiating OAC is only for the period of time that AF remains subclinical or below a critical burden threshold.

There are several potential explanations for why we did not see a reduction in recurrent stroke in the ICM vs control group. First, our study was not powered to detect such a difference. Second, 24% of patients with AF in the ICM group were not anticoagulated, limiting the clinical impact of AF detection on stroke prevention. Third, AFDAS is a progressive disease in which the burden increases over time. Many of the AF episodes detected by ICM were only minutes long and the risk of stroke and benefit of anticoagulation at this early stage may be lower than when AF is greater and able to be detected by less sensitive methods of sporadic monitoring or symptoms. Consistent with this, a recent analysis suggested that there is a temporal association of stroke with AF episodes greater vs less than 5.5 hours.^11^ The LOOP study failed to show a reduction in stroke incidence with monitoring compared with usual care in a high-risk population.^12^ A meta-analysis of randomized clinical trials showed a reduction in stroke associated with prolonged monitoring including several different monitoring modalities, although that analysis included both cryptogenic and noncryptogenic stroke patients.^13^ However, there may be residual or unmeasured confounding when comparing characteristics of patients with known AF vs AFDAS in other studies. Therefore, the clinical significance of our findings remains unknown.

### Limitations

Our study has several limitations. Treatment for AF was neither randomized nor prescribed by the study protocol, reasons for OAC use/nonuse were not captured and the study was not powered to detect differences in recurrent stroke rates. These features limit the conclusions that can be drawn about the clinical impact of AF detection. In addition, while the presumed etiology of index and recurrent stroke was determined by experienced stroke specialists and not centrally adjudicated, this approach reflects real-world clinical decision making. Lastly, the control group in our study did not include recently approved noninvasive wearable devices for AF detection so we cannot comment on their relative performance compared with an ICM.

## Conclusion

In summary, patients with ischemic stroke attributed to LAD or SVD face an increasing risk of AF over time and most of the AF occurrences are not reliably detected by standard medical monitoring methods. One year of negative monitoring should not reassure clinicians that stroke patients will not develop AF over the next 2 years.

## Notes

Supplement 1. eTable 1. Modifications made to the Clinical Investigation Plan after study initiation eTable 2: Baseline Characteristics in Participants in the Stroke of Known Cause and Underlying Atrial Fibrillation (STROKE AF) Trial eTable 3. Classification of First Recurrent Stroke Stratified by Index Stroke in Participants in the Stroke of Known Cause and Underlying Atrial Fibrillation (STROKE AF) Trial eTable 4: Univariate Predictors of AF Through 3 Years in Participants in the Stroke of Known Cause and Underlying Atrial Fibrillation (STROKE AF) Trial eFigure1: Time to First Detection of Atrial Fibrillation Through Study Duration in Participants in the Stroke of Known Cause and Underlying Atrial Fibrillation (STROKE AF) Trial eFigure 2: Recurrent strokes in Participants in the Stroke of Known Cause and Underlying Atrial Fibrillation (STROKE AF) Trial eFigure 3: Recurrent Strokes According to Index Stroke Subtype in Participants of the Stroke of Known Cause and Underlying Atrial Fibrillation (STROKE AF) Trial

Supplement 2. Nonauthor collaborators

Supplement 3. Trial protocol

Supplement 4. Data sharing statement
