# Supplementary material for: Atrial Fibrillation In Patients With Stroke Attributed to Large- or Small-Vessel Disease: 3-Year Results From the STROKE AF Randomized Clinical Trial
Source: JAMA Neurol. 2023 Oct 30;80(12):1277–83. doi: 10.1001/jamaneurol.2023.3931 (PMC10616765; doi:10.1001/jamaneurol.2023.3931)
Supplement: Supplement 1. — eTable 1. Modifications made to the Clinical Investigation Plan after study initiation eTable 2: Baseline Characteristics in Participants in the Stroke of Known Cause and Underlying Atrial Fibrillation (STROKE AF) Trial eTable 3. Classification of First Recurrent Stroke Stratified by Index Stroke in Participants in the Stroke of Known Cause and Underlying Atrial Fibrillation (STROKE AF) Trial eTable 4: Univariate Predictors of AF Through 3 Years in Participants in the Stroke of Known Cause and Underlying Atrial Fibrillation (STROKE AF) Trial eFigure1: Time to First Detection of Atrial Fibrillation Through Study Duration in Participants in the Stroke of Known Cause and Underlying Atrial Fibrillation (STROKE AF) Trial eFigure 2: Recurrent strokes in Participants in the Stroke of Known Cause and Underlying Atrial Fibrillation (STROKE AF) Trial eFigure 3: Recurrent Strokes According to Index Stroke Subtype in Participants of the Stroke of Known Cause and Underlying Atrial Fibrillation (STROKE AF) Trial [file jamaneurol-e233931-s001.pdf]

Supplemental Online Content

Bernstein RA, Kamel H, Granger CB, et al; STROKE AF Investigators. Atrial fibrillation in patients with stroke attributed to large- or small-vessel disease: 3-year results from the STROKE AF randomized clinical trial. *JAMA Neurol*. Published online October 30, 2023. doi:10.1001/jamaneurol.2023.3931

eTable 1. Modifications made to the Clinical Investigation Plan after study initiation

eTable 2: Baseline Characteristics in Participants in the Stroke of Known Cause and Underlying Atrial Fibrillation (STROKE AF) Trial

eTable 3. Classification of First Recurrent Stroke Stratified by Index Stroke in Participants in the Stroke of Known Cause and Underlying Atrial Fibrillation (STROKE AF) Trial

eTable 4: Univariate Predictors of AF Through 3 Years in Participants in the Stroke of Known Cause and Underlying Atrial Fibrillation (STROKE AF) Trial

eFigure1: Time to First Detection of Atrial Fibrillation Through Study Duration in Participants in the Stroke of Known Cause and Underlying Atrial Fibrillation (STROKE AF) Trial

eFigure 2: Recurrent strokes in Participants in the Stroke of Known Cause and Underlying Atrial Fibrillation (STROKE AF) Trial

eFigure 3: Recurrent Strokes According to Index Stroke Subtype in Participants of the Stroke of Known Cause and Underlying Atrial Fibrillation (STROKE AF) Trial

This supplemental material has been provided by the authors to give readers additional information about their work.

© 2023 Bernstein RA et al. JAMA Neurol.

eTable 1. Modifications made to the Clinical Investigation Plan after study initiation

CIP Version	Change	Rationale
4.0	From 200 patients with small-vessel disease (lacunar stroke) to a maximum of 50%	Allow equal characterization of an ischemic stroke population
4.0	Data Collection and Study Procedures were updated to allow either interrogation or remote CareLink transmission during follow-up (patients in ICM arm)	Include either mode of device data collection for patients completing visits in-clinic or remotely
3.0	Addition of AF related hospitalization to Ancillary Endpoint #7 related to describing economic burden of the disease	Business decision for additional analysis
3.0	Removed adjudication of ALL reported arrhythmias	Consistency with adjudication of time to first event
3.0	Removed “in-clinic” for Follow-up Procedure. Data can be collected remotely	Reduce patient burden of attending in-clinic as all data may be captured remotely
3.0	Enrollment timeline updated from 20 to 42 months and study from 4.5 to 6.5 years	Estimate updated per observed enrollment rate
3.0	Removed stopping rule for enrollment based on number of AF events (initially enrollment would be closed once the required number of AF events needed to demonstrate a statistical difference between hazard rates in the 2 arms was achieved)	Robust study with adequate data for analysis of all objectives

© 2023 Bernstein RA et al. JAMA Neurol.

eTable 2: Baseline Characteristics in Participants in the Stroke of Known Cause and Underlying Atrial Fibrillation (STROKE AF) Trial

Characteristic	ICM (n=242)[a]	Control (n=250)	ICM without an early exit or death (n=159)	Control without an early exit or death (n=155)
Age, Median (IQR) [N], years	66.0 (60.0-73.5), [240]	67.0 (61.0-75.0)	65.0 (59.0-73.0)	68.0 (61.0-75.0)
<65, n (%)	107/240 (44.2%)	108 (43.2%)	74 (46.5%)	56 (36.1%)
65 – 74, n (%)	81/240 (33.5%)	77 (30.8%)	53 (33.3%)	58 (37.4%)
75+, n (%)	52/240 (21.5%)	65 (26.0%)	32 (20.1%)	41 (26.5%)
Male, n (%)	144/240 (60.0%)	161 (64.4%)	94 (59.1%)	97 (62.6%)
Female, n (%)	96/240 (40.0%)	89 (35.6%)	65 (40.9%)	58 (37.4%)
QRS duration (ms), Median (IQR)	90 (82-100)	90 (84-102)	90 (82-100)	90 (82-101)
CHA_2DS_2-VASc Score, Median (IQR)[b]	5.0 (4.0 - 5.0)	5.0 (4.0 - 6.0)	5.0 (3.0 - 5.0)	5.0 (4.0 - 6.0)
Comorbidities/risk factors				
Congestive Heart Failure, n (%)	28 (11.6%)	23 (9.2%)	14 (8.8%)	11 (7.1%)
Hypertension, n (%)	197 (81.4%)	200 (80.0%)	129 (81.1%)	130 (83.9%)
Diabetes, n (%)	87 (36.0%)	100 (40.0%)	48 (30.2%)	62 (40.0%)
Stroke, n (%)	242 (100.0%)	250 (100.0%)	159 (100.0%)	155 (100.0%)
Vascular Disease, n (%)	45 (18.6%)	47 (18.8%)	27 (17.0%)	30 (19.4%)
Smoking tobacco, n (%)	130 (53.7%)	133 (53.2%)	95 (59.7%)	82 (52.9%)

© 2023 Bernstein RA et al. JAMA Neurol.

TOAST Classification[c]				
Large-artery disease	140 (57.9%)	142 (56.8%)	90 (56.6%)	79 (51.0%)
Small-vessel disease	100 (41.3%)	108 (43.2%)	69 (43.4%)	76 (49.0%)
CT Only	13 (5.4%)	11 (4.4%)	8 (5.0%)	6 (3.9%)
MRI Only	11 (4.5%)	20 (8.0%)	6 (3.8%)	11 (7.1%)
Both CT and MRI	211 (87.2%)	219 (87.6%)	143 (89.9%)	138 (89.0%)
Neither CT nor MRI	7 (2.9%)	0 (0.0%)	2 (1.3%)	0 (0.0%)
Score on NIH Stroke Scale, Median (IQR)[d]	2.0 (1.0 – 4.0)	2.0 (1.0 – 5.0)	2.0 (0.0 – 4.0)	2.0 (1.0 – 4.0)

Abbreviation: TIA: transient ischemic attack.

[a] Unless otherwise noted. Two patients in the ICM group for whom age and sex are not known exited the study early. One patient exited the same day of randomization and the other patient exited a day after being randomized. Both patients met the inclusion criterion stating that the subject had an ischemic stroke believed to be due to small-vessel disease, large vessel cervical or intracranial atherosclerosis within the past 10 days of enrollment. This information was used to calculate a CHA_2DS_2-VASc Score of 2. Type of qualifying stroke event was provided at the enrollment case report form and was used as the TOAST Classification of Subtypes of Acute Ischemic Stroke.

[b] Scores on the CHA_2DS_2-VASc risk assessment range from 0 to 9, with higher scores indicating a greater risk of stroke. A score of 5 corresponds to an estimated stroke risk of 7.2% per year.[13]

[c] The TOAST (Trial of Org 10172 in Acute Stroke Treatment) classification categorizes subtypes of ischemic stroke based on etiology into: 1) large-artery atherosclerosis (or large-vessel disease), 2) cardioembolism, 3) small-vessel occlusion (or small-vessel disease), 4) stroke of other determined

© 2023 Bernstein RA et al. JAMA Neurol.

etiology, and 5) stroke of undetermined etiology (either due to multiple competing causes or no cause identified).

© 2023 Bernstein RA et al. JAMA Neurol.

eTable 3. Classification of First Recurrent Stroke Stratified by Index Stroke in Participants in the Stroke of Known Cause and Underlying Atrial Fibrillation (STROKE AF) Trial

Recurrent stroke type	Index stroke n (%)			
	ICM		Control	
	Large-artery disease	Small-vessel disease	Large-artery disease	Small-vessel disease
TOAST				
Cardioembolic	1 (4.8%)	3 (23.1%)	1 (4.8%)	0
Large-artery disease	5 (23.8%)	1 (7.7%)	12 (57.1%)	1 (10.0%)
Small-vessel disease	7 (33.3%)	3 (23.1%)	2 (9.5%)	5 (50.0%)
Stroke of other determined etiology	0	0	1 (4.8%)	2 (20.0%)
Stroke of undetermined etiology	3 (14.3%)	3 (23.1%)	3 (14.3%)	2 (20.0%)
TOAST N/A	3 (14.3%)	1 (7.7%)	1 (4.8%)	0
Hemorrhagic	2 (9.5%)	2 (15.4%)	1 (4.8%)	0
**All**	**21**	**13**	**21**	**10**

© 2023 Bernstein RA et al. JAMA Neurol.

eTable 4: Univariate Predictors of AF Through 3 Years in Participants in the Stroke of Known Cause and Underlying Atrial Fibrillation (STROKE AF) Trial

Predictor	N	Hazard Ratio (95% CI)	P value
Baseline Characteristics			
Age, years	240	1.04 (1.01, 1.07)	**0.02**
BMI, kg/m^2	240	1.05 (1.00, 1.09)	**0.04**
Sex, Female	240	1.27 (0.71, 2.27)	0.418
Heart Rate, bpm	238	0.99 (0.96, 1.01)	0.296
Systolic Blood Pressure, mmHg	240	1.00 (0.99, 1.02)	0.655
Diastolic Blood Pressure, mmHg	240	0.98 (0.96, 1.00)	**0.089**
Testing Results			
Left Atrial Diameter, mm	166	1.04 (1.00, 1.09)	**0.05**
Left Atrial Enlargement	214	2.24 (1.21, 4.16)	**0.01**
Left Atrial Volume Index, mL/m^2	142	1.05 (1.02, 1.08)	**0.001**
Left Atrial Volume Index (+10 mL/m^2)	142	1.65 (1.22, 2.22)	**0.001**
QRS Duration, ms	219	1.02 (1.00, 1.03)	**0.012**
PR Interval, ms	218	1.00 (0.99, 1.01)	0.613
RR Interval, ms	180	1.00 (1.00, 1.00)	0.353

© 2023 Bernstein RA et al. JAMA Neurol.

QTc interval, ms	219	1.00 (1.00, 1.01)	0.338
Qualifying stroke			
Side (left vs right)	240	0.80 (0.45, 1.44)	0.717
Stroke type (LAD or SVD)	242	1.19 (0.67, 2.13)	0.552
Infarction location			
Brainstem	240	1.23 (0.59, 2.55)	0.575
Cerebellum	240	0.82 (0.30, 2.30)	0.711
Cerebral Artery, Anterior	240	0.73 (0.23, 2.35)	0.595
Cerebral Artery, Middle	240	1.07 (0.60, 1.91)	0.814
Cerebral Artery, Posterior	240	1.48 (0.73, 2.98)	0.276
No ischemic area showing on image	240	0.57 (0.08, 4.17)	0.584
Modified Rankin Score	239	1.00 (0.83, 1.21)	0.969
NIHSS Score	240	1.01 (0.94, 1.09)	0.73
Medical History			
Aortic Plaque	240	N/A[a]	0.989
Cerebral Artery Stenosis	240	1.19 (0.47, 3.02)	0.712
Cerebral Artery Revascularization or Percutaneous Intervention	240	N/A[a]	0.987

© 2023 Bernstein RA et al. JAMA Neurol.

CHA_2DS_2-VASc Score	240	1.53 (1.22, 1.92)	**<0.001**
Chronic obstructive pulmonary disease (COPD)	240	1.91 (0.76, 4.84)	0.172
Congestive Heart Failure	240	4.12 (1.47, 11.52)	**0.007**
Coronary Artery Disease	240	1.55 (0.77, 3.12)	0.22
Coronary artery bypass graft (CABG)	240	1.61 (0.58, 4.50)	0.362
Coronary artery intervention	240	1.78 (0.79, 3.97)	0.161
Depression	240	1.21 (0.58, 2.50)	0.61
Diabetes	240	1.29 (0.71, 2.33)	0.4
Insulin dependent	240	1.41 (0.70, 2.84)	0.34
Non-insulin dependent	240	1.05 (0.49, 2.24)	0.91
Hypertension	240	2.52 (0.90, 7.02)	**0.078**
Myocardial Infarction	240	1.97 (0.83, 4.64)	0.123
Obstructive Sleep Apnea	240	1.06 (0.38, 2.96)	0.91
Peripheral Vascular Disease	240	1.92 (0.86, 4.28)	0.113
Renal Dysfunction	240	2.91 (1.30, 6.50)	**0.009**
Sleep Apnea: Central or Obstructive	240	1.06 (0.38, 2.96)	0.91

© 2023 Bernstein RA et al. JAMA Neurol.

Stroke or stroke-related event diagnosed prior to qualifying stroke event	240	1.21 (0.65, 2.27)	0.55
Hemorrhagic Stroke	240	N/A[a]	0.987
Ischemic stroke, cryptogenic	240	N/A[a]	0.988
Ischemic stroke, of known origin	240	1.53 (0.79, 2.96)	0.205
Thromboembolism	240	2.81 (0.39, 20.41)	0.307
Transient Ischemic Attack	240	0.67 (0.21, 2.16)	0.503

[a] Wald 95% confidence intervals for hazard ratios were not calculated due to very small proportions of occurrence.

© 2023 Bernstein RA et al. JAMA Neurol.

eFigure1: Time to First Detection of Atrial Fibrillation Through Study Duration in Participants in the Stroke of Known Cause and Underlying Atrial Fibrillation (STROKE AF) Trial

Participants with Large-Artery Disease

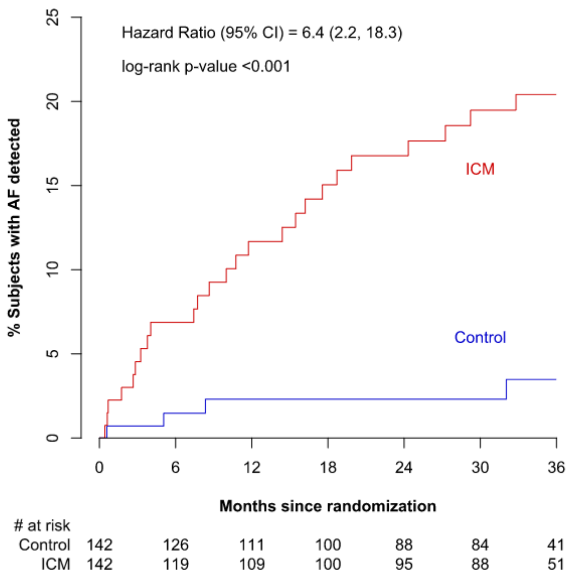

Participants with Small-Vessel Disease

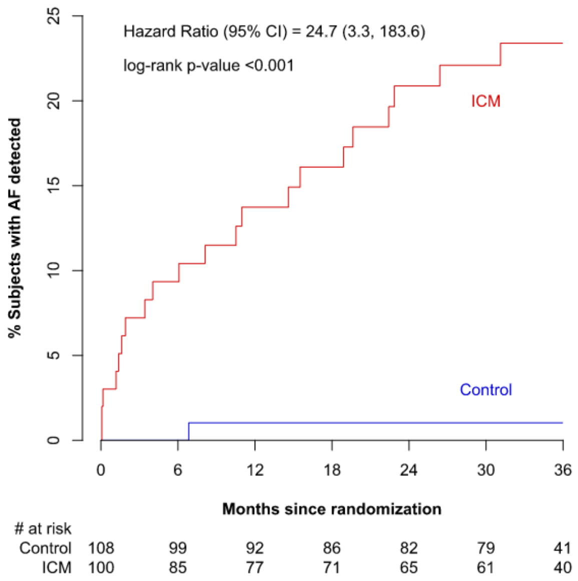

© 2023 Bernstein RA et al. JAMA Neurol.

eFigure 2: Recurrent strokes in Participants in the Stroke of Known Cause and Underlying Atrial Fibrillation (STROKE AF) Trial

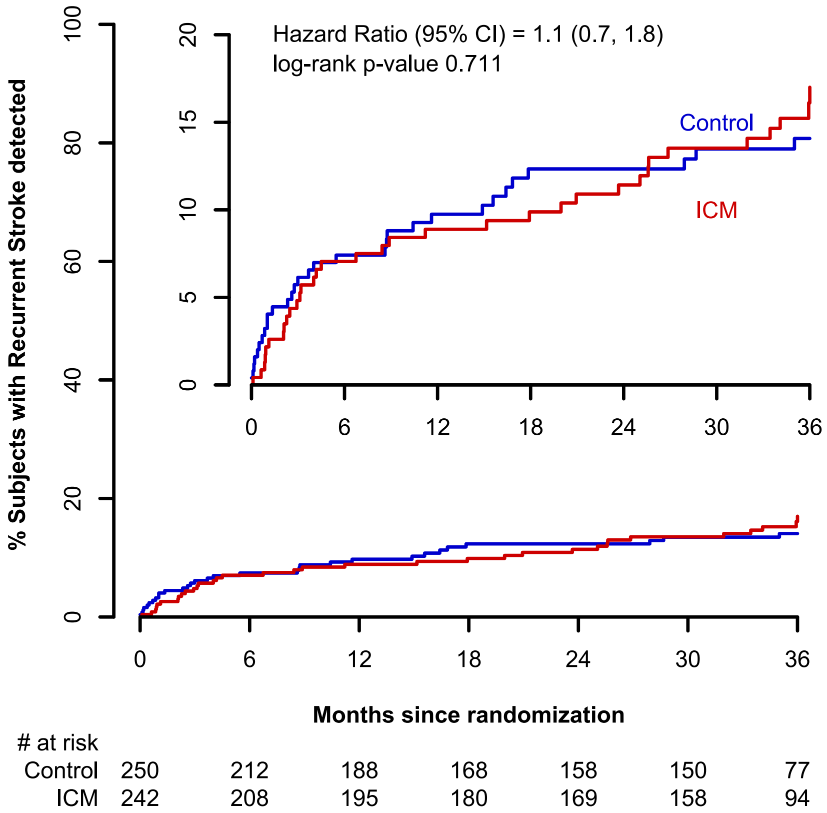

© 2023 Bernstein RA et al. JAMA Neurol.

eFigure 3: Recurrent Strokes According to Index Stroke Subtype in Participants of the Stroke of Known Cause and Underlying Atrial Fibrillation (STROKE AF) Trial

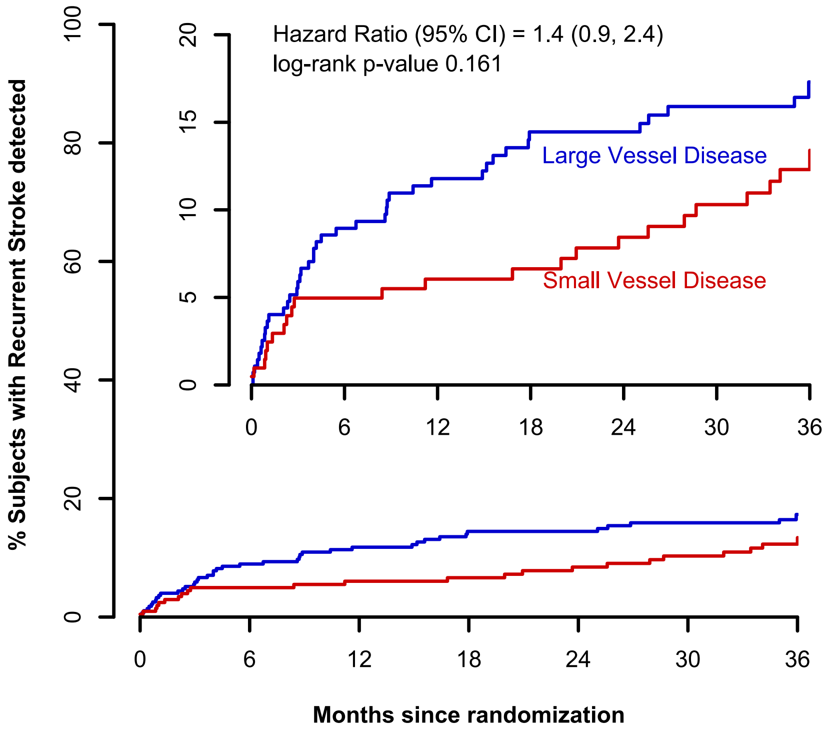

# at risk							
Large Vessel	284	236	211	189	178	169	91
Small Vessel	208	184	172	159	149	139	80

© 2023 Bernstein RA et al. JAMA Neurol.
