# Supplementary material for: Atrial Fibrillation In Patients With Stroke Attributed to Large- or Small-Vessel Disease: 3-Year Results From the STROKE AF Randomized Clinical Trial
Source: JAMA Neurol. 2023 Oct 30;80(12):1277–83. doi: 10.1001/jamaneurol.2023.3931 (PMC10616765; doi:10.1001/jamaneurol.2023.3931)
Supplement: Supplement 2. — Nonauthor collaborators [file jamaneurol-e233931-s002.pdf]

Supplemental Online Content: Nonauthor Collaborators
*First name, last name, and suffix (if applicable) are required and will appear in PubMed.

*Group Name(s): STROKE AF Investigators							
***First Name and Middle Initial(s)**	***Last Name**	***Suffix (eg, Jr, III)**	Academic Degrees	Institution	Location (city, state/province, country)	Role or Contribution, eg, chair, principal investigator	Group (if more than 1 Group listed in the byline) and/or Subgroup (eg, Steering Committee)
Indrani	Acosta			AdventHealth Neuroscience Institute	Orlando, FL, United States	Principal Investigator	
Pradipkumar	Jamnadas			AdventHealth Neuroscience Institute	Orlando, FL, United States	Sub-Investigator	
Sushma	Manda			AdventHealth Neuroscience Institute	Orlando, FL, United States	Sub-Investigator	
Rizwan	Alimohammad			Albany Medical Center	Albany, NY, United States	Principal Investigator	
Valerie	Arias			Albany Medical Center	Albany, NY, United States	Sub-Investigator	
Kristi	Tempro			Albany Medical Center	Albany, NY, United States	Sub-Investigator	
Kathleen	Ward			Albany Medical Center	Albany, NY, United States	Sub-Investigator	
Khaled	Asi			Aurora Saint Luke’s Medical Center	Milwaukee, WI, United States	Former PI	
Indrajit	Choudhuri			Aurora Saint Luke’s Medical Center	Milwaukee, WI, United States	Sub-Investigator	
Waldo	Guerrero			Aurora Saint Luke’s Medical Center	Milwaukee, WI, United States	Sub-Investigator	
Junaid	Kalia			Aurora Saint Luke’s Medical Center	Milwaukee, WI, United States	Sub-Investigator	
Imran	Niazi			Aurora Saint Luke’s Medical Center	Milwaukee, WI, United States	Sub-Investigator	
Rehan	Sajjad			Aurora Saint Luke’s Medical Center	Milwaukee, WI, United States	Sub-Investigator	
Varoon	Thavapalan			Aurora Saint Luke’s Medical Center	Milwaukee, WI, United States	Principal Investigator	

Supplemental Online Content: Nonauthor Collaborators
*First name, last name, and suffix (if applicable) are required and will appear in PubMed.

***First Name and Middle Initial(s)**	***Last Name**	***Suffix (eg, Jr, III)**	Academic Degrees	Institution	Location (city, state/province, country)	Role or Contribution, eg, chair, principal investigator	Group (if more than 1 Group listed in the byline) and/or Subgroup (eg, Steering Committee)
Manish	Assar			Baylor Research Institute Dallas - Baylor University Medical Center (BUMC)	Dallas, TX, United States	Sub-Investigator	
Javier	Banchs			Baylor Research Institute Dallas - Baylor University Medical Center (BUMC)	Dallas, TX, United States	Sub-Investigator	
James	Black			Baylor Research Institute Dallas - Baylor University Medical Center (BUMC)	Dallas, TX, United States	Sub-Investigator	
Peter	Cheung			Baylor Research Institute Dallas - Baylor University Medical Center (BUMC)	Dallas, TX, United States	Sub-Investigator	
Alan	Donsky			Baylor Research Institute Dallas - Baylor University Medical Center (BUMC)	Dallas, TX, United States	Sub-Investigator	
Dion	Graybeal			Baylor Research Institute Dallas - Baylor University Medical Center (BUMC)	Dallas, TX, United States	Sub-Investigator	
Rashedul	Hasan			Baylor Research Institute Dallas - Baylor University Medical Center (BUMC)	Dallas, TX, United States	Principal Investigator	
Osman	Mir			Baylor Research Institute Dallas - Baylor University Medical Center (BUMC)	Dallas, TX, United States	Sub-Investigator	
Claude	Nguyen			Baylor Research Institute Dallas - Baylor University Medical Center (BUMC)	Dallas, TX, United States	Sub-Investigator	
Gregory	Olsovsky			Baylor Research Institute Dallas - Baylor University Medical Center (BUMC)	Dallas, TX, United States	Sub-Investigator	

Supplemental Online Content: Nonauthor Collaborators
*First name, last name, and suffix (if applicable) are required and will appear in PubMed.

***First Name and Middle Initial(s)**	***Last Name**	***Suffix (eg, Jr, III)**	Academic Degrees	Institution	Location (city, state/province, country)	Role or Contribution, eg, chair, principal investigator	Group (if more than 1 Group listed in the byline) and/or Subgroup (eg, Steering Committee)
Jennifer	Rasmussen			Baylor Research Institute Dallas - Baylor University Medical Center (BUMC)	Dallas, TX, United States	Sub-Investigator	
Sanjeev	Hasabnis			Cardiovascular Associates of the Southeast	Birmingham, AL, United States	Sub-Investigator	
Russell	Reeves			Cardiovascular Associates of the Southeast	Birmingham, AL, United States	Principal Investigator	
Chris	Rowley			Cardiovascular Associates of the Southeast	Birmingham, AL, United States	Former PI	
Jitendra	Sharma			Cardiovascular Associates of the Southeast	Birmingham, AL, United States	Sub-Investigator	
Macey	Smith			Cardiovascular Associates of the Southeast	Birmingham, AL, United States	Sub-Investigator	
Kay	Bonyak			Centra Medical Group Stroobants Cardiovascular Center	Lynchburg, VA, United States	Sub-Investigator	
Matthew	Sackett			Centra Medical Group Stroobants Cardiovascular Center	Lynchburg, VA, United States	Principal Investigator	
James	Allred			Cone Health	Greensboro, NC, United States	Sub-Investigator	
Pramod	Sethi			Cone Health	Greensboro, NC, United States	Principal Investigator	
Richard	Jung			Cox Medical Center South	Springfield, MO, United States	Principal Investigator	
Jennifer	Lynch			Cox Medical Center South	Springfield, MO, United States	Principal Investigator	
Steven	Rowe			Cox Medical Center South	Springfield, MO, United States	Sub-Investigator	
Subasini	Dash			Hackensack University Medical Center	Hackensack, NJ, United States	Principal Investigator	
Taya	Glotzer			Hackensack University Medical Center	Hackensack, NJ, United States	Sub-Investigator	

*First name, last name, and suffix (if applicable) are required and will appear in PubMed.

***First Name and Middle Initial(s)**	***Last Name**	***Suffix (eg, Jr, III)**	Academic Degrees	Institution	Location (city, state/province, country)	Role or Contribution, eg, chair, principal investigator	Group (if more than 1 Group listed in the byline) and/or Subgroup (eg, Steering Committee)
Sameer	Jamal			Hackensack University Medical Center	Hackensack, NJ, United States	Sub-Investigator	
Glauco	Radoslovich			Hackensack University Medical Center	Hackensack, NJ, United States	Sub-Investigator	
Gunjan	Shukla			Hackensack University Medical Center	Hackensack, NJ, United States	Sub-Investigator	
John	Zimmerman			Hackensack University Medical Center	Hackensack, NJ, United States	Sub-Investigator	
Haitham	Hussein			HealthPartners Institute (Bloomington MN)	Bloomington, MN, United States	Principal Investigator	
Dennis	Zhu			HealthPartners Institute (Bloomington MN)	Bloomington, MN, United States	Sub-Investigator	
Kristopher	Krueger			HealthPartners Institute (Saint Louis Park MN)	Saint Louis Park, MN, United States	Sub-Investigator	
Matthew	Ostrander			HealthPartners Institute (Saint Louis Park MN)	Saint Louis Park, MN, United States	Principal Investigator	
Darwin	Ramirez			HealthPartners Institute (Saint Louis Park MN)	Saint Louis Park, MN, United States	Principal Investigator	
Jeffrey	Shultz			HealthPartners Institute (Saint Louis Park MN)	Saint Louis Park, MN, United States	Sub-Investigator	
Jay	Simonson			HealthPartners Institute (Saint Louis Park MN)	Saint Louis Park, MN, United States	Sub-Investigator	
Brett	Cucchiara			Hospital of the University of Pennsylvania	Philadelphia, PA, United States	Sub-Investigator	
Rajat	Deo			Hospital of the University of Pennsylvania	Philadelphia, PA, United States	Sub-Investigator	
David	Frankel			Hospital of the University of Pennsylvania	Philadelphia, PA, United States	Sub-Investigator	
Judy	Jia			Hospital of the University of Pennsylvania	Philadelphia, PA, United States	Sub-Investigator	

Supplemental Online Content: Nonauthor Collaborators
*First name, last name, and suffix (if applicable) are required and will appear in PubMed.

***First Name and Middle Initial(s)**	***Last Name**	***Suffix (eg, Jr, III)**	Academic Degrees	Institution	Location (city, state/province, country)	Role or Contribution, eg, chair, principal investigator	Group (if more than 1 Group listed in the byline) and/or Subgroup (eg, Steering Committee)
Scott	Kasner			Hospital of the University of Pennsylvania	Philadelphia, PA, United States	Principal Investigator	
Jeanie	Luciano			Hospital of the University of Pennsylvania	Philadelphia, PA, United States	Sub-Investigator	
Steven	Messe			Hospital of the University of Pennsylvania	Philadelphia, PA, United States	Sub-Investigator	
Michael	Mullen			Hospital of the University of Pennsylvania	Philadelphia, PA, United States	Sub-Investigator	
Pasquale	Santangeli			Hospital of the University of Pennsylvania	Philadelphia, PA, United States	Sub-Investigator	
Robert	Schaller			Hospital of the University of Pennsylvania	Philadelphia, PA, United States	Sub-Investigator	
Qingyang	Yuan			Hospital of the University of Pennsylvania	Philadelphia, PA, United States	Sub-Investigator	
Don	Bledsoe			Houston Methodist Hospital	Houston,TX, United States	Sub-Investigator	
Christian	Cajavilca			Houston Methodist Hospital	Houston,TX, United States	Sub-Investigator	
David	Chiu			Houston Methodist Hospital	Houston,TX, United States	Sub-Investigator	
Rajan	Gadhia			Houston Methodist Hospital	Houston,TX, United States	Sub-Investigator	
Maranda Randi	Grimes			Houston Methodist Hospital	Houston,TX, United States	Sub-Investigator	
Larry	Katz			Houston Methodist Hospital	Houston,TX, United States	Sub-Investigator	
Stacy	Moye			Houston Methodist Hospital	Houston,TX, United States	Sub-Investigator	
Tapan	Rami			Houston Methodist Hospital	Houston,TX, United States	Sub-Investigator	

Supplemental Online Content: Nonauthor Collaborators
*First name, last name, and suffix (if applicable) are required and will appear in PubMed.

***First Name and Middle Initial(s)**	***Last Name**	***Suffix (eg, Jr, III)**	Academic Degrees	Institution	Location (city, state/province, country)	Role or Contribution, eg, chair, principal investigator	Group (if more than 1 Group listed in the byline) and/or Subgroup (eg, Steering Committee)
Abraham	Thomas			Houston Methodist Hospital	Houston,TX, United States	Sub-Investigator	
John	Volpi			Houston Methodist Hospital	Houston,TX, United States	Principal Investigator	
Ali	Al Balushi			Icahn School of Medicine at Mount Sinai	New York, NY, United States	Sub-Investigator	
Clara	Boyd			Icahn School of Medicine at Mount Sinai	New York, NY, United States	Sub-Investigator	
Subbarao	Choudry			Icahn School of Medicine at Mount Sinai	New York, NY, United States	Sub-Investigator	
Mandip	Dhamoon			Icahn School of Medicine at Mount Sinai	New York, NY, United States	Principal Investigator	
Srinivas	Dukkipati			Icahn School of Medicine at Mount Sinai	New York, NY, United States	Sub-Investigator	
Davida	Goltz			Icahn School of Medicine at Mount Sinai	New York, NY, United States	Sub-Investigator	
Qing	Hao			Icahn School of Medicine at Mount Sinai	New York, NY, United States	Sub-Investigator	
Deborah	Horowitz			Icahn School of Medicine at Mount Sinai	New York, NY, United States	Sub-Investigator	
Gurmeen	Kaur			Icahn School of Medicine at Mount Sinai	New York, NY, United States	Sub-Investigator	
Jacob	Koruth			Icahn School of Medicine at Mount Sinai	New York, NY, United States	Sub-Investigator	
Christeena	Kurian			Icahn School of Medicine at Mount Sinai	New York, NY, United States	Sub-Investigator	
Marie-Noelle	Langan			Icahn School of Medicine at Mount Sinai	New York, NY, United States	Sub-Investigator	
Ivan	Matos Diaz			Icahn School of Medicine at Mount Sinai	New York, NY, United States	Sub-Investigator	

Supplemental Online Content: Nonauthor Collaborators
*First name, last name, and suffix (if applicable) are required and will appear in PubMed.

***First Name and Middle Initial(s)**	***Last Name**	***Suffix (eg, Jr, III)**	Academic Degrees	Institution	Location (city, state/province, country)	Role or Contribution, eg, chair, principal investigator	Group (if more than 1 Group listed in the byline) and/or Subgroup (eg, Steering Committee)
Marc	Miller			Icahn School of Medicine at Mount Sinai	New York, NY, United States	Sub-Investigator/Implanter	
Vivek	Reddy			Icahn School of Medicine at Mount Sinai	New York, NY, United States	Sub-Investigator	
Kara	Sheinart			Icahn School of Medicine at Mount Sinai	New York, NY, United States	Sub-Investigator	
Laura	Stein			Icahn School of Medicine at Mount Sinai	New York, NY, United States	Sub-Investigator	
Aaron	Tansy			Icahn School of Medicine at Mount Sinai	New York, NY, United States	Sub-Investigator	
Stanley	Tuhrim			Icahn School of Medicine at Mount Sinai	New York, NY, United States	Sub-Investigator	
Jesse	Weinberger			Icahn School of Medicine at Mount Sinai	New York, NY, United States	Sub-Investigator	
William	Whang			Icahn School of Medicine at Mount Sinai	New York, NY, United States	Sub-Investigator	
Jonathan	Cross			Innovative Medical Research of South Florida	Miami, FL, United States	Sub-Investigator	
Howard	Kreger			Innovative Medical Research of South Florida	Miami, FL, United States	Principal Investigator	
Marc	Saltzman			Innovative Medical Research of South Florida	Miami, FL, United States	Sub-Investigator	
Kenneth	Zide			Innovative Medical Research of South Florida	Miami, FL, United States	Sub-Investigator	
Sandeep	Bansal			Lancaster General Hospital	Lancaster, PA , United States	Principal Investigator	
Matthew	Bernabei			Lancaster General Hospital	Lancaster, PA , United States	Sub-Investigator	
Murray	Flaster			Lancaster General Hospital	Lancaster, PA , United States	Principal Investigator	

Supplemental Online Content: Nonauthor Collaborators
*First name, last name, and suffix (if applicable) are required and will appear in PubMed.

***First Name and Middle Initial(s)**	***Last Name**	***Suffix (eg, Jr, III)**	Academic Degrees	Institution	Location (city, state/province, country)	Role or Contribution, eg, chair, principal investigator	Group (if more than 1 Group listed in the byline) and/or Subgroup (eg, Steering Committee)
Conor	Barrett			Massachusetts General Hospital	Boston, MA, United States	Sub-Investigator	
Mark	Etherton			Massachusetts General Hospital	Boston, MA, United States	Sub-Investigator	
E. Kevin	Heist			Massachusetts General Hospital	Boston, MA, United States	Sub-Investigator	
Steven	Lubitz			Massachusetts General Hospital	Boston, MA, United States	Sub-Investigator	
Robert	Regenhardt			Massachusetts General Hospital	Boston, MA, United States	Sub-Investigator	
Richa	Sharma			Massachusetts General Hospital	Boston, MA, United States	Sub-Investigator	
Scott	Silverman			Massachusetts General Hospital	Boston, MA, United States	Principal Investigator	
Kelly	Sloane			Massachusetts General Hospital	Boston, MA, United States	Sub-Investigator	
Anand	Viswanathan			Massachusetts General Hospital	Boston, MA, United States	Sub-Investigator	
Amit	Doshi			Mercy Hospital Saint Louis	St. Louis, MO, United States	Sub-Investigator	
William	Logan			Mercy Hospital Saint Louis	St. Louis, MO, United States	Sub-Investigator	
Maheen	Malik			Mercy Hospital Saint Louis	St. Louis, MO, United States	Sub-Investigator	
David	Rempe			Mercy Hospital Saint Louis	St. Louis, MO, United States	Principal Investigator	
Rohan	Arora			North Shore University Hospital	Manhasset, NY, United States	Sub-Investigator	
Stuart	Beldner			North Shore University Hospital	Manhasset, NY, United States	Sub-Investigator	

Supplemental Online Content: Nonauthor Collaborators
*First name, last name, and suffix (if applicable) are required and will appear in PubMed.

***First Name and Middle Initial(s)**	***Last Name**	***Suffix (eg, Jr, III)**	Academic Degrees	Institution	Location (city, state/province, country)	Role or Contribution, eg, chair, principal investigator	Group (if more than 1 Group listed in the byline) and/or Subgroup (eg, Steering Committee)
Laurence	Epstein			North Shore University Hospital	Manhasset, NY, United States	Sub-Investigator	
Haisam	Ismail			North Shore University Hospital	Manhasset, NY, United States	Sub-Investigator	
Ram	Jadonath			North Shore University Hospital	Manhasset, NY, United States	Sub-Investigator	
Jeffrey	Katz			North Shore University Hospital	Manhasset, NY, United States	Principal Investigator	
Richard	Libman			North Shore University Hospital	Manhasset, NY, United States	Sub-Investigator	
Mohammad	Moussavi			North Shore University Hospital	Manhasset, NY, United States	Sub-Investigator	
Anand	Patel			North Shore University Hospital	Manhasset, NY, United States	Sub-Investigator	
Apoor	Patel			North Shore University Hospital	Manhasset, NY, United States	Sub-Investigator	
Jonathan	Willner			North Shore University Hospital	Manhasset, NY, United States	Sub-Investigator	
Paul	Wright			North Shore University Hospital	Manhasset, NY, United States	Principal Investigator	
Frances	Caprio			Northwestern University	Evanston, IL, United States	Principal Investigator	
Susan	Kim			Northwestern University	Evanston, IL, United States	Sub-Investigator	
Scott	Mendelson			Northwestern University	Evanston, IL, United States	Sub-Investigator	
Rod	Passman			Northwestern University	Evanston, IL, United States	Sub-Investigator	
Ilana	Ruff			Northwestern University	Evanston, IL, United States	Sub-Investigator	

Supplemental Online Content: Nonauthor Collaborators
*First name, last name, and suffix (if applicable) are required and will appear in PubMed.

***First Name and Middle Initial(s)**	***Last Name**	***Suffix (eg, Jr, III)**	Academic Degrees	Institution	Location (city, state/province, country)	Role or Contribution, eg, chair, principal investigator	Group (if more than 1 Group listed in the byline) and/or Subgroup (eg, Steering Committee)
Mohammad	Alsorogi			Norton Neuroscience Institute	Louisville, KY, United States	Sub-Investigator	
Gauhar	Chaudhary			Norton Neuroscience Institute	Louisville, KY, United States	Sub-Investigator	
Kent	Morris			Norton Neuroscience Institute	Louisville, KY, United States	Sub-Investigator	
Savannah	Mullins			Norton Neuroscience Institute	Louisville, KY, United States	Sub-Investigator	
Nadeem	Talpur			Norton Neuroscience Institute	Louisville, KY, United States	Principal Investigator	
Kevin	Thomas			Norton Neuroscience Institute	Louisville, KY, United States	Sub-Investigator	
Breehan	Chancellor			NYU Langone Medical Center	New York, NY, United States	Sub-Investigator	
Larry	Chinitz			NYU Langone Medical Center	New York, NY, United States	Sub-Investigator	
Andre	Culpepper			NYU Langone Medical Center	New York, NY, United States	Sub-Investigator	
Michael	Fara			NYU Langone Medical Center	New York, NY, United States	Sub-Investigator	
Koto	Ishida			NYU Langone Medical Center	New York, NY, United States	Principal Investigator	
Kaitlyn	Lillemoe			NYU Langone Medical Center	New York, NY, United States	Sub-Investigator	
Aaron	Lord			NYU Langone Medical Center	New York, NY, United States	Sub-Investigator	
Jose	Torres			NYU Langone Medical Center	New York, NY, United States	Sub-Investigator	
Cen	Zhang			NYU Langone Medical Center	New York, NY, United States	Sub-Investigator	

Supplemental Online Content: Nonauthor Collaborators
*First name, last name, and suffix (if applicable) are required and will appear in PubMed.

***First Name and Middle Initial(s)**	***Last Name**	***Suffix (eg, Jr, III)**	Academic Degrees	Institution	Location (city, state/province, country)	Role or Contribution, eg, chair, principal investigator	Group (if more than 1 Group listed in the byline) and/or Subgroup (eg, Steering Committee)
Freddy	Abi-Samra			Ochsner Medical Center	New Orleans, LA, United States	Sub-Investigator	
Michael	Bernard			Ochsner Medical Center	New Orleans, LA, United States	Sub-Investigator	
Jacqueline	Carter			Ochsner Medical Center	New Orleans, LA, United States	Sub-Investigator	
Himanshu	Chokhawala			Ochsner Medical Center	New Orleans, LA, United States	Sub-Investigator	
Amanda	Downey			Ochsner Medical Center	New Orleans, LA, United States	Sub-Investigator	
Samuel	Khatib			Ochsner Medical Center	New Orleans, LA, United States	Sub-Investigator	
Daniel	Morin			Ochsner Medical Center	New Orleans, LA, United States	Sub-Investigator	
Glenn	Polin			Ochsner Medical Center	New Orleans, LA, United States	Sub-Investigator	
Paul	Rogers			Ochsner Medical Center	New Orleans, LA, United States	Sub-Investigator	
Gabriel	Vidal			Ochsner Medical Center	New Orleans, LA, United States	Sub-Investigator	
Richard	Zweifler			Ochsner Medical Center	New Orleans, LA, United States	Principal Investigator	
Gary	Belt			Overlook Medical Center	Summit, NJ, United States	Sub-Investigator	
Robert	Felberg			Overlook Medical Center	Summit, NJ, United States	Principal Investigator	
Steve	Furer			Overlook Medical Center	Summit, NJ, United States	Sub-Investigator	
John	Hanna			Overlook Medical Center	Summit, NJ, United States	Sub-Investigator	

Supplemental Online Content: Nonauthor Collaborators
*First name, last name, and suffix (if applicable) are required and will appear in PubMed.

***First Name and Middle Initial(s)**	***Last Name**	***Suffix (eg, Jr, III)**	Academic Degrees	Institution	Location (city, state/province, country)	Role or Contribution, eg, chair, principal investigator	Group (if more than 1 Group listed in the byline) and/or Subgroup (eg, Steering Committee)
Angela	McCall-Brown			Overlook Medical Center	Summit, NJ, United States	Sub-Investigator	
Susan	Seeger			Overlook Medical Center	Summit, NJ, United States	Sub-Investigator	
Ramakota	Reddy			PeaceHealth Sacred Heart Medical Center at Riverbend	Springfield, OR, United States	Sub-Investigator	
Elaine	Skalabrin			PeaceHealth Sacred Heart Medical Center at Riverbend	Springfield, OR, United States	Principal Investigator	
Diane	Soik			PeaceHealth Sacred Heart Medical Center at Riverbend	Springfield, OR, United States	Sub-Investigator	
Michael	Wilder			PeaceHealth Sacred Heart Medical Center at Riverbend	Springfield, OR, United States	Sub-Investigator	
Michelle	Kearney			Saint Thomas Research Institute	Nashville, TN, United States	Sub-Investigator	
Eugene	LaFranchise			Saint Thomas Research Institute	Nashville, TN, United States	Principal Investigator	
Brett	Parker			Saint Thomas Research Institute	Nashville, TN, United States	Sub-Investigator	
Robert Andrew (Drew)	Pickett			Saint Thomas Research Institute	Nashville, TN, United States	Sub-Investigator	
Heather	Bonaguidi			Scripps Memorial	San Diego, CA, United States	Sub-Investigator	
Douglas	Gibson			Scripps Memorial	San Diego, CA, United States	Sub-Investigator/Implanter	
Mary	Kalafut			Scripps Memorial	San Diego, CA, United States	Principal Investigator	
Beth	Mattera			Scripps Memorial	San Diego, CA, United States	Sub-Investigator	

Supplemental Online Content: Nonauthor Collaborators
*First name, last name, and suffix (if applicable) are required and will appear in PubMed.

***First Name and Middle Initial(s)**	***Last Name**	***Suffix (eg, Jr, III)**	Academic Degrees	Institution	Location (city, state/province, country)	Role or Contribution, eg, chair, principal investigator	Group (if more than 1 Group listed in the byline) and/or Subgroup (eg, Steering Committee)
Nicholas	Olson			Scripps Memorial	San Diego, CA, United States	Sub-Investigator/Implanter	
Mary	Parker			Scripps Memorial	San Diego, CA, United States	Sub-Investigator	
John	Rogers			Scripps Memorial	San Diego, CA, United States	Sub-Investigator	
Poulina	Uddin			Scripps Memorial	San Diego, CA, United States	Sub-Investigator	
Cherylee	Chang			The Queen's Medical Center	Honolulu, HI, United States	Former PI	
Sarah	Graner			The Queen's Medical Center	Honolulu, HI, United States	Sub-Investigator	
Joseph	Kipta			The Queen's Medical Center	Honolulu, HI, United States	Sub-Investigator	
Rony	Salem			The Queen's Medical Center	Honolulu, HI, United States	Principal Investigator	
David	Singh			The Queen's Medical Center	Honolulu, HI, United States	Sub-Investigator/Implanter	
Ahmad	Al-Awwad			The University of Oklahoma Health Sciences Center (OUHSC)	Oklahoma City, OK, United States	Sub-Investigator	
Bahar	Beaver			The University of Oklahoma Health Sciences Center (OUHSC)	Oklahoma City, OK, United States	Sub-Investigator	
Shuchi	Chaudhary			The University of Oklahoma Health Sciences Center (OUHSC)	Oklahoma City, OK, United States	Sub-Investigator	
Stephen	Clayton			The University of Oklahoma Health Sciences Center (OUHSC)	Oklahoma City, OK, United States	Sub-Investigator	
Claire	Delpirou Nouh			The University of Oklahoma Health Sciences Center (OUHSC)	Oklahoma City, OK, United States	Sub-Investigator	

Supplemental Online Content: Nonauthor Collaborators
*First name, last name, and suffix (if applicable) are required and will appear in PubMed.

***First Name and Middle Initial(s)**	***Last Name**	***Suffix (eg, Jr, III)**	Academic Degrees	Institution	Location (city, state/province, country)	Role or Contribution, eg, chair, principal investigator	Group (if more than 1 Group listed in the byline) and/or Subgroup (eg, Steering Committee)
Paul	Garabelli			The University of Oklahoma Health Sciences Center (OUHSC)	Oklahoma City, OK, United States	Sub-Investigator	
David	Gordon			The University of Oklahoma Health Sciences Center (OUHSC)	Oklahoma City, OK, United States	Sub-Investigator	
Aneesh	Pakala			The University of Oklahoma Health Sciences Center (OUHSC)	Oklahoma City, OK, United States	Sub-Investigator	
Bappaditya	Ray			The University of Oklahoma Health Sciences Center (OUHSC)	Oklahoma City, OK, United States	Sub-Investigator	
Scott	Saucedo			The University of Oklahoma Health Sciences Center (OUHSC)	Oklahoma City, OK, United States	Sub-Investigator	
Evgeny	Sidorov			The University of Oklahoma Health Sciences Center (OUHSC)	Oklahoma City, OK, United States	Principal Investigator	
Stavros	Stavrakis			The University of Oklahoma Health Sciences Center (OUHSC)	Oklahoma City, OK, United States	Sub-Investigator	
Marilou	Ching			University at Buffalo, The State University of New York	Buffalo, NY, United States	Principal Investigator	
Christopher	Deline			University at Buffalo, The State University of New York	Buffalo, NY, United States	Sub-Investigator	
J. Maurice	Hourihane			University at Buffalo, The State University of New York	Buffalo, NY, United States	Sub-Investigator	
Amit	Kandel			University at Buffalo, The State University of New York	Buffalo, NY, United States	Sub-Investigator	
Chee	Kim			University at Buffalo, The State University of New York	Buffalo, NY, United States	Sub-Investigator	
Rakesh	Magun			University at Buffalo, The State University of New York	Buffalo, NY, United States	Sub-Investigator	
Ashkan	Mowla			University at Buffalo, The State University of New York	Buffalo, NY, United States	Principal Investigator	
Robert	Sawyer			University at Buffalo, The State University of New York	Buffalo, NY, United States	Sub-Investigator	

Supplemental Online Content: Nonauthor Collaborators
*First name, last name, and suffix (if applicable) are required and will appear in PubMed.

***First Name and Middle Initial(s)**	***Last Name**	***Suffix (eg, Jr, III)**	Academic Degrees	Institution	Location (city, state/province, country)	Role or Contribution, eg, chair, principal investigator	Group (if more than 1 Group listed in the byline) and/or Subgroup (eg, Steering Committee)
Donald	Switzer			University at Buffalo, The State University of New York	Buffalo, NY, United States	Sub-Investigator	
Nitish	Badhwar			University of California San Francisco UCSF Medical Center	San Francisco, CA, United States	Sub-Investigator	
Randall	Lee			University of California San Francisco UCSF Medical Center	San Francisco, CA, United States	Sub-Investigator	
Karl	Meisel			University of California San Francisco UCSF Medical Center	San Francisco, CA, United States	Principal Investigator	
Wade	Smith			University of California San Francisco UCSF Medical Center	San Francisco, CA, United States	Sub-Investigator	
Moayd	Alkahalifah			University of Miami Hospital	Miami, FL, United States	Sub-Investigator	
Sushanth	Aroor			University of Miami Hospital	Miami, FL, United States	Sub-Investigator	
Negar	Asdaghi			University of Miami Hospital	Miami, FL, United States	Sub-Investigator	
Nirav	Bhatt			University of Miami Hospital	Miami, FL, United States	Sub-Investigator	
Victor	Del Brutto			University of Miami Hospital	Miami, FL, United States	Sub-Investigator	
George	Dillon			University of Miami Hospital	Miami, FL, United States	Sub-Investigator	
Paul	Gadient			University of Miami Hospital	Miami, FL, United States	Sub-Investigator	
Sebastian	Koch			University of Miami Hospital	Miami, FL, United States	Principal Investigator	
Litsa	Lambrakos			University of Miami Hospital	Miami, FL, United States	Sub-Investigator	
Amer	Malik			University of Miami Hospital	Miami, FL, United States	Sub-Investigator	
Erika	Marulanda-Londono			University of Miami Hospital	Miami, FL, United States	Sub-Investigator	
Zeeshan	Memon			University of Miami Hospital	Miami, FL, United States	Sub-Investigator	
Ivan	Mendoza			University of Miami Hospital	Miami, FL, United States	Sub-Investigator	
Raul	Mitrani			University of Miami Hospital	Miami, FL, United States	Sub-Investigator	
Gustavo	Ortiz			University of Miami Hospital	Miami, FL, United States	Sub-Investigator	
Jose	Ramano			University of Miami Hospital	Miami, FL, United States	Sub-Investigator	
Nicole	Sur			University of Miami Hospital	Miami, FL, United States	Sub-Investigator	
Luis	Torres			University of Miami Hospital	Miami, FL, United States	Sub-Investigator	
James	Daniels			University of Texas (UT) Southwestern Medical Center	Dallas, TX, United States	Sub-Investigator	

Supplemental Online Content: Nonauthor Collaborators
*First name, last name, and suffix (if applicable) are required and will appear in PubMed.

***First Name and Middle Initial(s)**	***Last Name**	***Suffix (eg, Jr, III)**	Academic Degrees	Institution	Location (city, state/province, country)	Role or Contribution, eg, chair, principal investigator	Group (if more than 1 Group listed in the byline) and/or Subgroup (eg, Steering Committee)
Mark	Johnson			University of Texas (UT) Southwestern Medical Center	Dallas, TX, United States	Principal Investigator	
Alejandro	Magadan			University of Texas (UT) Southwestern Medical Center	Dallas, TX, United States	Sub-Investigator	
Ty	Shang			University of Texas (UT) Southwestern Medical Center	Dallas, TX, United States	Sub-Investigator	
Nancy	Mcclelland			Medtronic	Mounds View, MN, United States	Clinical trial leader	
Theodore	Merriam			Medtronic	Mounds View, MN, United States	Statistician	
Karah	Neisen			Medtronic	Mounds View, MN, United States	Program manager	
