# Supplementary material for: Atrial Fibrillation In Patients With Stroke Attributed to Large- or Small-Vessel Disease: 3-Year Results From the STROKE AF Randomized Clinical Trial
Source: JAMA Neurol. 2023 Oct 30;80(12):1277–83. doi: 10.1001/jamaneurol.2023.3931 (PMC10616765; doi:10.1001/jamaneurol.2023.3931)
Supplement: Supplement 3. — Trial protocol [file jamaneurol-e233931-s003.pdf]

Medtronic

Clinical Investigation Plan

**Clinical Investigation Plan/Study Title**	Stroke AF
**Study Product Name**	Reveal LINQ™ Insertable Cardiac Monitor
**Sponsor/Local Sponsor**	Medtronic, Inc.<br>8200 Coral Sea Street NE<br>Mounds View, MN U.S.A. 55112<br>1 (800) 328-2518
**Document Version**	4.0<br>18 December 2018

Confidentiality Statement

The information contained in this document is confidential and the proprietary property of Medtronic. Any distribution, copying, or disclosure without the prior written authorization of Medtronic is strictly prohibited. Persons to whom the information is disclosed must know that it is confidential and that it may not be further disclosed by them.

Table of Contents

Table of Contents **2**
Listing of Tables **6**
1. Glossary **7**
2. Synopsis **10**
3. Introduction **14**
3.1. Background 14
3.2. Purpose 16
4. Objectives and Endpoints **16**
4.1. Objectives 16
4.1.1. Primary Objective(s) 16
4.1.2. Secondary Objective(s) 17
5. Study Design **17**
5.1. Study Design 17
5.1.1. Minimization of Bias 17
5.2. Duration 18
5.3. Rationale 18
6. Product Description **19**
6.1. General 19
6.2. Reveal LINQ System 19
6.3. Intended Population 21
6.4. Packaging 21
6.5. Equipment 21
6.6. Product Use 22
6.7. Product Training Requirements 22
6.7.1. Investigator / Investigation Site Selection 22
6.7.2. Site Activation 22
6.8. Product Storage 23
6.9. Product Return 23

7. Selection of Subjects....23
7.1. Study Population....23
7.2. Subject Enrollment....23
7.3. Inclusion Criteria....24
7.4. Exclusion Criteria....24
8. Study Procedures....25
8.1. Schedule of Events....25
8.2. Subject Consent....27
8.3. Randomization and Treatment Assignment....29
8.4. Procedures and Data Collection....29
8.4.1. Baseline....29
8.4.2. Reveal LINQ ICM Insertion....30
8.4.3. Scheduled Follow-up Visits/CareLink Transmissions....31
8.4.3.1. Follow-up Visits....32
8.4.3.2. Follow-up Visits / Remote CareLink Transmissions....33
8.4.4. Unscheduled Follow-up Visits....33
8.4.5. Stroke/TIA Recurrence....33
8.4.6. System Modification....33
8.4.7. Study Exit....34
8.5. Assessment of Safety....35
8.6. Recording Data....35
8.7. Deviation Handling....36
8.8. Subject Withdrawal or Discontinuation....37
9. Risks and Benefits....37
9.1. Potential Risks....37
9.2. Potential Benefits....37
9.3. Risk-Benefit Rationale....37
10. Adverse Events and Device Deficiencies....38
10.1. Adverse Event Assessment....38
10.1.1. Adverse Events....38
10.1.2. Device Deficiencies....39

10.1.3. Processing Updates and Resolution 39
10.2. Definitions/Classifications 39
10.2.1. Adverse Event and Device Deficiency Definitions 39
10.2.2. Adverse Events and Deficiency Classification 43
10.3. Reporting of Adverse Events 44
10.3.1. Subject Death 45
10.3.1.1. Death Data Collection 45
10.3.1.2. Death Classification and Reporting 47
10.4. Product Complaint Reporting 47
11. Data Review Committees 48
11.1. Clinical Events Committee (CEC) Review 48
12. Statistical Design and Methods 49
12.1. Primary Objective 49
i. Hypothesis 50
ii. Endpoint Definition 50
iii. Performance Requirements 50
iv. Rationale for Performance Criteria 50
v. Sample Size Determination 51
vi. Analysis Methods 51
vii. Determination of Patients/Data for Analysis 51
12.2. Secondary Objective 52
i. Hypothesis 52
ii. Endpoint Definition 52
iii. Performance Requirements 52
iv. Sample Size Determination 52
v. Analysis Methods 53
vi. Determination of Patients/Data for Analysis 53
13. Ethics 53
13.1. Statement(s) of Compliance 53
14. Study Administration 54

14.1. Monitoring 54
14.1.1. Monitoring Visits....54
14.2. Data Management 54
14.3. Direct Access to Source Data/Documents 55
14.4. Confidentiality 55
14.5. Liability 55
14.6. CIP Amendments....55
14.7. Record Retention....56
14.7.1. Investigator Records 56
14.7.2. Investigator Reports.... 56
14.7.3. Sponsor Records.... 57
14.7.4. Sponsor Reports 58
14.8. Publication and Use of Information 58
14.8.1. Publication Committee 58
14.8.2. Management of Primary, Secondary and Ancillary Publications 59
14.8.3. Criteria for Determining Authorship.... 59
14.8.4. Transparency.... 60
14.9. Suspension or Early Termination....60
14.9.1. Planned Study Closure 60
14.9.2. Early Termination or Suspension 60
14.9.2.1. Study-wide Termination or Suspension 61
14.9.2.2. Investigator/Site Termination or Suspension 61
14.9.3. Procedures for Termination or Suspension.... 61
14.9.3.1. Medtronic-Initiated and Regulatory Authority-Initiated 61
14.9.3.2. Investigator-Initiated.... 62
14.9.3.3. Ethics Committee-Initiated 62
15. References 63
16. Version History 65

Listing of Tables

Table 1: Reveal LINQ System Component Information 21
Table 2: Data Collection and Study Procedures 26
Table 3: Reveal LINQ ICM Programming 31
Table 4: Follow-up Visit / CareLink Transmission Schedule 32
Table 5: Recommended Programming Parameters for Medtronic IPG, ICD or CRT Devices 34
Table 6: Adverse Event Definitions 40
Table 7: Adverse Event Classification Responsibilities 44
Table 8: AE Reporting Requirements 45
Table 9: Subject Death Classification 47
Table 10: Investigator Reports per Medtronic Requirements 57
Table 11: Sponsor Reports 58

1. Glossary

Term	Definition
Adverse Event (AE)<br>(Adapted from ISO 14155:2011, 3.2)	Any untoward medical occurrence, unintended disease or injury, or untoward clinical signs (including abnormal laboratory findings) in subjects, users or other persons, whether or not related to the investigational medical device<br>NOTE 1: This definition includes events related to the investigational medical device or the comparator.<br>NOTE 2: This definition includes events related to the procedures involved.
Adverse Device Effect (ADE)<br>(ISO 14155:2011, 3.1)	Adverse event related to the use of an investigational medical device<br>NOTE 1: This definition includes adverse events resulting from insufficient or inadequate instructions for use, deployment, implantation, installation, or operation, or any malfunction of the investigational medical device.<br>NOTE 2: This definition includes any event resulting from an error use or from intentional misuse of the investigational medical device.
American Heart Association (AHA)	A non-profit organization in the United States that fosters appropriate cardiac care in an effort to reduce disability and deaths caused by cardiovascular disease and stroke.
Antiplatelet (AP)	Antiplatelet agents decrease platelet aggregation (clumping together) and inhibit thrombus formation.
Atrial Fibrillation (AF)	Atrial fibrillation (also called AFib or AF) is a quivering or irregular heartbeat (arrhythmia) that can lead to blood clots, stroke, heart failure and other heart-related complication.
Cardiac Death	A death directly related to the electrical or mechanical dysfunction of the heart.
Cardiac Resynchronization Therapy (CRT)	CRT is a clinically proven treatment option for some individuals with heart failure. A CRT device sends small electrical impulses to both lower chambers of the heart to help them beat together in a more synchronized pattern. This may improve the heart's ability to pump blood and oxygen to your body.
Cardiovascular Related	An adverse event relating to the heart and the blood vessels or the circulation.
CHADS Score	Clinical prediction rules for estimating the risk of stroke in patients with non-rheumatic atrial fibrillation. A high score corresponds to a greater risk of stroke, while a low score corresponds to a lower risk of stroke.
Carotid Endarterectomy (CEA)	A surgical procedure used to reduce the risk of stroke by correcting stenosis in the common carotid artery or internal carotid artery. Endarterectomy is the removal of material on the inside of an artery.
Clinical Event Committee (CEC)	An independent committee of experts not participating in the clinical study that provides adjudication of study specific endpoints and/or events utilizing study-specific or consensus definitions available in the field.
Clinical Investigation Plan (CIP)	The present document describing the study protocol.
Complication	An adverse event that results in death, involves any termination of significant device function, or requires invasive intervention.<br>Non-invasive: when applied to a diagnostic device or procedure, means one that does not by design or intention:

Term	Definition
	• Penetrate or pierce the skin or mucous membranes of the body, the ocular cavity or the urethra or<br>• Enter the ear beyond the external auditory canal, the nose beyond the nares, the mouth beyond the pharynx, the anal canal beyond the rectum, or the vagina beyond the cervical os<br>For purposes of this part, blood sampling that involves simple venipuncture is considered non-invasive, and the use of surplus samples of body fluid or tissue that are left over from samples taken for non-investigational purposes is also considered non-invasive.
Coronary Artery Bypass Graft Surgery (CABG)	Coronary artery bypass surgery, also known as coronary artery bypass graft surgery, and colloquially heart bypass or bypass surgery, is a surgical procedure to restore normal blood flow to an obstructed coronary artery.
Cryptogenic Stroke	A stroke without an obvious explanation (of unknown origin).
Device Deficiency (DD)<br>(ISO 14155:2011, 3.15)	Inadequacy of a medical device with respect to its identity, quality, durability, reliability, safety or performance.<br>NOTE: Device deficiencies include malfunctions, use errors and inadequate labeling
Electronic Case Report Forms (eCRF)	Forms where the clinical data are collected. eCRF is the electronic version of case report forms.
Ethical Committee / Institutional Review Board (EC / IRB)	Any board, committee, or other group formally designated by an institution to review, to approve the initiation of, and to conduct periodic review of, biomedical research involving human subjects. The primary purpose of such review is to assure the protection of the rights and welfare of the human subjects.
Hospitalization	A therapeutic inpatient hospitalization (excludes outpatient and emergency room visits) lasting greater than or equal to 24 hours.
Insertable Cardiac Defibrillator (ICD)	A device implantable inside the body, able to perform cardioversion, defibrillation, and pacing of the heart.
Insertable Cardiac Monitor (ICM)	An implantable loop recorder, also known as an insertable cardiac monitor, is a small device that is implanted just under the skin of the chest for long term cardiac monitoring.
Ischemic Stroke (IS)	Ischemic stroke occurs as a result of an obstruction within a blood vessel supplying blood to the brain.
Non-cardiac Death	A death not classified as a cardiac death
Non-sudden Cardiac Death	All cardiac deaths that are not classified as sudden deaths, including all cardiac deaths of hospitalized subjects on inotropic support.
Observation	Any adverse event that is not a complication.<br>Note 1: Only system or procedure related AEs will be classified as Complication or Observation.
Oral anticoagulation (OAC)	Anticoagulant agents prevent or reduce coagulation of blood.
Remote Data Capture (RDC)	An interface that allows site users at sites to enter data directly into the study database via a web interface. RDC is an example of Electronic Data Capture method or EDC.
Reveal LINQ ICM Procedure Related	An adverse event that occurs that is directly related to the implantation or modification of the Reveal LINQ system.

Term	Definition
Reveal LINQ ICM System Related	An adverse event that results from the presence or performance (intended or otherwise) of any component of the system (including: Reveal LINQ ICM, Programmer, Patient Assistant, and MyCareLink Patient Monitor).
Serious Adverse Event (SAE)<br>(Adapted from ISO 14155:2011, 3.37)	Adverse event that<br>a) led to death,<br>b) led to serious deterioration in the health of the subject, that either resulted in<br>1. a life-threatening illness or injury, or<br>2. a permanent impairment of a body structure or a body function, or<br>3. in-patient or prolonged hospitalization, or<br>4. medical or surgical intervention to prevent life-threatening illness or injury or permanent impairment to a body structure or a body function,<br>c) led to fetal distress, fetal death or a congenital abnormality or birth defect<br>NOTE 1: Planned hospitalization for a pre-existing condition, or a procedure required by the CIP, without serious deterioration in health, is not considered a serious adverse event.
Stroke	Hemorrhagic or Ischemic stroke with rapid onset of a focal or global neurological deficit or other neurological signs/symptoms consistent with stroke.
Stroke Diagnosis	Diagnosis will be determined by the site’s neurologist clinical evaluation and include clinical signs and symptoms consistent with focal or global neurological deficit in addition to one or more of the following:<br>• Neuroimaging procedure (CT, MR, or cerebral angiogram)<br>• Lumbar Puncture (i.e. subarachnoid hemorrhage)
Sudden Cardiac Death (SCD)	Natural death due to cardiac causes, indicated by abrupt loss of consciousness within one hour of the onset of acute symptoms; preexisting heart disease may have been known to be present, but the time and mode of death are unexpected. If time of onset cannot be determined, SCD will alternatively be defined as any unexpected cardiac death occurring out of the hospital or in the emergency room as dead on arrival.
Transient Ischemic Attack (TIA)	New focal neurological deficit with rapid symptom resolution (usually 1 – 2 hours), always within 24 hours without tissue injury (based on neuroimaging).
Unavoidable Adverse Event	An Adverse Event inherent to a surgical procedure that is expected to occur in all subjects for a projected duration according to the Investigator’s opinion, including, but not limited to:

<table>
<tr><th>Term</th><th>Definition</th></tr>
<tr><td></td><td>
<table>
<tr><th>Event Description</th><th>Timeframe (hours) from the Surgical Procedure</th></tr>
<tr><td>Anesthesia related nausea / vomiting</td><td>24</td></tr>
<tr><td>Low-grade fever (<100°F or 37.8°C)</td><td>48</td></tr>
<tr><td>Pocket site / Incisional pain</td><td>72</td></tr>
<tr><td>Mild to moderate bruising / ecchymosis</td><td>168</td></tr>
<tr><td>Sleep problems (insomnia)</td><td>72</td></tr>
<tr><td>Back pain related to laying on table</td><td>72</td></tr>
<tr><td>Shoulder pain/discomfort/stiffness related to shoulder immobilization during procedure</td><td>72</td></tr>
</table>
</td></tr>
<tr><td>Unknown</td><td>There is insufficient or inadequate information to classify the death.</td></tr>
</table>

2. Synopsis

**Title**	**Stroke AF**
**Clinical Study Type**	Post Market
**Product Name**	Medtronic Reveal LINQ™ System
**Sponsor**	Medtronic, Inc.<br>8200 Coral Sea Street NE<br>Mounds View, MN 55112<br>U.S.A.
**Indication under investigation**	The Reveal LINQ system is being used in accordance with the indications for the device.
**Investigation Purpose**	The purpose of the Stroke AF study is to compare the incidence of atrial fibrillation (AF) through 12 months between continuous cardiac rhythm monitoring with the Reveal LINQ™ Insertable Cardiac Monitor (ICM) (continuous monitoring arm) and standard of care (SoC) medical treatment (control arm) in subjects with a recent ischemic stroke of presumed known origin.
**Product Status**	The Stroke AF study will be conducted using the components of the Medtronic Reveal LINQ system, as described in the below.

	Model Number: LNQ11 or other commercial model — Component: Reveal LINQ Insertable Cardiac Monitor — Market- released

Model Number	Component	Investigational or Market-released
LNQ11 or other commercial model	Reveal LINQ Insertable Cardiac Monitor	Market- released
2090 or other commercial model	Medtronic Programmer	Market- released
960000 or other commercial model	Patient Assistant	Market- released
24950 or other commercial model	MyCareLink Patient Monitor	Market- released

**Primary Objective**	To compare the incidence rate of AF through 12 months between the continuous monitoring arm and the control arm in subjects with a recent ischemic stroke of presumed known origin.
**Secondary Objective**	To compare the incidence rates of AF through the duration of study follow-up between study arms.
**Ancillary Objectives**	1.
**Study Design**	Stroke AF is a prospective, multi-site, randomized, controlled, non-blinded, post-market study. The Stroke AF study will compare the incidence rate of AF through 12 months between the continuous monitoring arm and the control arm in subjects with a recent ischemic stroke of presumed known origin.
**Randomization**	Once enrolled, subjects will be randomized 1:1 (continuous monitoring vs control).
**Treatment**	Subjects randomized to the continuous monitoring arm will have a Reveal LINQ ICM inserted within 10 days of the qualifying stroke and undergo continuous remote monitoring. Subjects randomized to the control arm will be followed per site specific standard of care.
**Sample Size**	The study will be conducted at approximately 40 study sites in the United States (U.S.). Approximately 496 subjects may be enrolled in the study. It is anticipated that it will take approximately 42 months to complete study enrollments. Subjects will be followed for 3 years, until end of device life or until official study closure, whichever occurs first.
**Inclusion Criteria**	• Subject has had an ischemic stroke believed to be due to small vessel disease, large vessel cervical or intracranial atherosclerosis within the past 10 days<br>• Subject is willing and able to undergo study requirements and expected to be geographically stable during study follow-up<br>• Subject is 60 years of age or older, or age 50 to 59 years plus a documented medical history of at least one of the following additional risk factors for stroke:<br>o Congestive heart failure<br>o Hypertension (Systolic Blood Pressure >140)<br>o Diabetes Mellitus

	o Prior Stroke (>90 days ago, other than study qualifying index event)<br>o Vascular disease (e.g. coronary artery disease, heart attack, peripheral artery disease and complex aortic plaque)
**Exclusion Criteria**	• Subject has had a cryptogenic stroke<br>• Subject has had a cardioembolic stroke<br>• Subject has untreated hyperthyroidism<br>• Subject has had a recent myocardial infarction <1 month of stroke<br>• Subject has had a recent cardiac surgery (e.g. coronary artery bypass surgery (CABG)) <1 month of stroke<br>• Subject has a mechanical heart valve<br>• Subject has valvular disease requiring immediate surgical intervention<br>• Subject has documented prior history of atrial fibrillation or atrial flutter<br>• Subject has permanent indication for oral anticoagulation<br>• Subject has permanent contraindication to oral anticoagulation such that detection of AF would not change medical management, based on enrolling investigators judgment<br>• Subject is enrolled in a concurrent study that may confound the results of this study. Co-enrollment in any concurrent clinical study (including registries) requires approval of the study manager or designee.<br>• Subject's life expectancy is less than 1 year<br>• Subject is pregnant<br>• Subject has or is indicated for implant with a pacemaker, ICD, CRT, or an implantable hemodynamic monitor<br>• Subject with a medical condition that precludes the patient from participation in the opinion of the investigator
**Safety Assessments**	All Reveal LINQ ICM system/component-related, Reveal LINQ ICM procedure related, cardiovascular related AEs, and all serious adverse events leading to death will be collected throughout the study duration, starting at the time of signing the consent form.

167

Medtronic

Clinical Procedures

Study Procedure	Baseline	LINQ Insertion*	1, 6 and 12 Month Follow-up	3, 9 Months Remote Follow-up*	Recurring 6 Month Follow-up (through study exit)	Unscheduled Follow-up	Stroke/TIA Recurrence
Informed Consent	X						
Inclusion/Exclusion Assessment	X						
Randomization	X						
Demographics, Medical History	X						
Cardiovascular Medications	X		X		X		
TOAST1	X						X
NIH Stroke Scale	X						X
Modified Rankin Scale	X		X				X
EQ-5D	X		X				
Blood Draw	X (if available)						
System & Procedure Information*		X*					
Full Reveal LINQ Interrogation or CareLink Transmission*		X*	X*	X*	X*	X* (if available)	X* (if available)
AF Assessment			X		X	X	X
Symptom Assessment			X		X	X	
Stroke/TIA Assessment			X		X	X	
Adverse Events	As they occur						
Device Deficiencies							
System Modifications*							
Crossover							
Study Deviations							
Subject Exits							
Death							

[1]Trial of Org 10172 in Acute Stroke Treatment

** Subjects with a Reveal LINQ ICM*

3. Introduction

3.1. Background

Stroke is a devastating disease. In the United States (U.S.) more than 780,000 people experience a new or recurrent stroke each year; approximately 600,000 of these are first attacks and 180,000 are recurrent attacks. About 20% of patients die within the first year after having a stroke and another 20% are bound to a nursing home for the rest of their life. Furthermore, stroke is the fifth leading cause of death in the U.S. (when considered separately from other cardiovascular diseases) and the leading cause of serious long-term disability in the U.S. (Dariush Mozaffarian, 2015).

Of all strokes, 87% are ischemic (Dariush Mozaffarian, 2015), which occur as a result of decreased or insufficient blood flow to the brain. Patients with ischemic stroke (IS) are often treated with antithrombotic therapy. For most patients with IS, the mainstay of treatment is anti-platelet (AP) therapy with aspirin, clopidogrel, or aspirin+extended release dipyridamole. However, patients with IS who have been diagnosed with atrial fibrillation (AF) are normally treated with long-term oral anticoagulation (OAC), per recommendations within the AHA/ASA 2014 guidelines. OAC is dramatically more effective at preventing recurrent IS than AP therapy in patients with AF (Owen, 2009). In contrast, for patients without AF (or other rare cardiac sources of embolism), OAC has not been shown more effective than AP therapy. Therefore, detection of AF after IS is crucial, as it results in an important change in the administration of antithrombotic therapy from AP to OAC, thereby reducing the risk of recurrent stroke.

Episodes of AF are often asymptomatic, which makes detection of AF based on patient's symptoms unreliable (L. M. Christensen, 2014) (S. Adam Strickberger, 2005). Additionally, due to its episodic nature, AF is difficult to detect via intermittent monitoring techniques (Paul D. Ziegler, 2009). While AHA/ASA guidelines recommend cardiac monitoring of patients for up to 30 days after stroke, the optimum duration of monitoring is not known. It has generally been shown that longer durations of cardiac monitoring result in higher rates of AF detection after stroke. The advent of insertable cardiac monitors (ICM) allows continuous monitoring to detect infrequent episodes of AF for up to 3 years. Longer monitoring periods may be necessary to detect infrequent but clinically important paroxysms of AF (William C. Choe, 2015).

Studies to date suggest that AF is present in ~4-25% of patients with a recent IS of presumed known origin, but this has only been examined using short term (up to 3 weeks) or intermittent monitoring. Rabinstein et al (2013) compared the rate of detection of AF over a three-week period of cardiac monitoring in 66 patients with cryptogenic stroke to an equal number of patients with stroke of known origin. Overall, there was no difference in detection rate between cryptogenic stroke and stroke of known origin (25% vs 14%, respectively, P=0.12). However, in patients over 65 years, the rates of detection were 27% and 25% respectively, whereas in younger patients, more AF was found in those

with cryptogenic stroke (21% vs 0%, p=0.03). Similarly, Sposato et al (2012) found that continuous cardiac monitoring during hospitalization found newly diagnosed AF in a substantial number of patients with non-cryptogenic stroke. Other studies have shown that the presence of atherosclerotic risk factors as denoted by the CHADS2 score predicts the risk of newly detected AF after IS, regardless of stroke mechanism. Lastly, Grond et al (2013) found that of a total of 1135 patients enrolled post stroke or TIA, unknown AF was detected in 49 patients (4.3% by 72-hour ECG). In summary, these data suggest that AF is present in patients with a recent IS of presumed known origin, but this has only been examined using short term (up to 3 weeks) or intermittent monitoring. To date, there is yet to be a published study that has used an ICM for AF detection specifically in patients with stroke of presumed known origin.

The occurrence of a first stroke dramatically increases the risk of a second stroke. About 25% of all IS occur in patients who have had a prior IS (recurrent IS) (Ralph L. Sacco, et al., 2006). Recurrent ischemic strokes are generally more severe than first strokes, and recurrent strokes are more costly, disabling, and fatal. Additionally, recurrent stroke is the most likely major adverse event after a first IS. Therefore, in patients with a first stroke, prevention of recurrence (secondary prevention) is the primary goal of therapy.

Secondary prevention of IS rests on determining and addressing the risk factors for future stroke. For example, patients with stroke ipsilateral to carotid stenosis are treated with carotid endarterectomy (CEA), which has been proven to reduce the risk of recurrence. Although strokes are classified by mechanism, definitive identification of the cause of stroke is often difficult, and many patients have multiple potential causes or have no clear cause. It is therefore not always possible to tailor stroke prevention to the cause of the prior stroke. Rather, preventive therapies are usually directed at identifiable causes of future stroke. Importantly, the effectiveness of a preventive therapy does not prove that the target of that therapy caused the index stroke. Rather, it proves that the intervention addressed a risk factor for future stroke.

Both persistent AF and paroxysmal AF are potent predictors of first and recurrent stroke. More than 75,000 cases of stroke per year are attributed to AF (Ralph L. Sacco, et al., 2006). AF is a major risk factor in this patient population, and it often goes undetected due to its episodic and asymptomatic nature. While guidelines do recommend cardiac monitoring of patients after stroke, the optimum duration of monitoring in all patients with stroke is not known. For most patients standard of care appears to involve monitoring these patients for 24 hours with a Holter monitor. It has generally been shown that longer durations of cardiac monitoring have better rates of detection of AF after stroke. The advent of insertable cardiac monitors (ICM) allows detection of infrequent episodes of AF for up to 3 years.

This clinical study was designed to address known gaps in the clinical evidence related to the incidence of AF in patients with an IS. ISs are generally classified according to their presumed cause. However, there is a subset of patients who have suffered stroke for which a cause cannot be determined. These patients are said to have suffered a cryptogenic stroke, or an embolic stroke of an unknown source

(ESUS). The CRYSTAL AF study demonstrated that continuous monitoring via ICM is superior to standard of care for detecting AF after cryptogenic stroke. At 6 months, AF had been detected at a rate of 8.9% in the ICM group vs 1.4% of patients in the control group. By 12 months, AF had been detected at a rate of 12.4% in the ICM group vs 2.0% in the control group (Tommaso Sanna, 2014). While the CRYSTAL AF data is promising for patients with a cryptogenic stroke, it remains unclear what the long-term rate of AF is in the IS patient population with a presumed known origin. Many post IS patients have risk factors for AF, regardless of the cause of their stroke. Newly detected episodes of AF were found in 28% of patients with previous thromboembolic events in the TRENDS study (Paul D. Ziegler, et al., 2010) and most of these episodes would not have been detected by standard intermittent monitoring.

3.2. Purpose

Medtronic is sponsoring the Stroke AF study. This is a prospective, multi-site, randomized, controlled, non-blinded, post-market study.

The purpose of the Stroke AF study is to compare the incidence rate of AF through 12 months between continuous cardiac rhythm monitoring and standard of care in subjects with a recent IS of presumed known origin (the cause of the stroke is not unknown), specifically due to small vessel disease, large vessel cervical or intracranial atherosclerosis. Upon enrollment subjects will be randomized 1:1 to continuous monitoring arm vs control arm. Subjects randomized to the continuous monitoring arm will have a Reveal LINQ™ ICM inserted within 10 days of the index event and undergo continuous monitoring. Subjects randomized to the control arm will be followed per site specific standard of care. Detection of AF after IS is crucial, as it results in an important change in antithrombotic therapy from AP to OAC, thereby reducing the risk of recurrent stroke in these patients.

This study supports introducing Reveal LINQ ICM into the care pathway in subjects with an ischemic stroke of presumed known origin.

4. Objectives and Endpoints

4.1. Objectives

The Stroke AF study will compare the incidence rate of AF through 12 months between continuous monitoring and standard of care in subjects with a recent ischemic stroke of presumed known origin. Subjects will be randomized 1:1 to the continuous monitoring arm or control arm.

4.1.1. Primary Objective(s)

To compare the incidence rate of AF through 12 months between the continuous monitoring arm vs control arm in subjects with a recent ischemic stroke of presumed known origin.

4.1.2. Secondary Objective(s)

To compare the incidence rates of AF through the duration of study follow-up between study arms.

5. Study Design

5.1. Study Design

Stroke AF is a prospective, multi-site, randomized, controlled, non-blinded, post-market study. The Stroke AF study will compare the incidence rate of AF through 12 months between the continuous monitoring arm and the control arm in subjects with a recent ischemic stroke of presumed known origin. Once enrolled, subjects will be randomized 1:1 (continuous monitoring vs control). Subjects randomized to the continuous monitoring arm will have a Reveal LINQ ICM inserted within 10 days of the qualifying stroke and undergo continuous remote monitoring. Subjects randomized to the control arm will be followed per site specific standard of care.

The study is expected to be conducted at approximately 40 sites located in the United States (U.S.).

It is estimated that approximately 496 subjects will be enrolled in the study. Assuming 2% attrition from enrollment to randomization, approximately 486 subjects will have randomized. A total of 23 first documented AF events within 12 months of randomization are required for the study to test the primary objective. AF will be defined as an AF event lasting more than 30 seconds and adjudicated by the endpoint adjudication committee. The analysis of the primary objective will occur after all enrolled subjects have completed 12 months of follow-up. Approximately 248 devices may be used in the study. Based on previous studies of this scope and magnitude, it is estimated that sites may identify and enroll on average one eligible subject per month.

To ensure a widespread distribution of data and minimize site bias in study results, the maximum number of subjects who may be enrolled at a single site is 50 subjects. Sites that enroll faster than others will be allowed to do so in order to maintain an adequate overall study enrollment rate. In addition, to ensure a robust dataset is available for subgroup analysis, a maximum of 50% (approximately248) subjects with lacunar stroke (i.e., small vessel disease) may be enrolled. Medtronic will notify study sites when enrollment of lacunar stroke subjects is no longer allowed, if applicable.

5.1.1. Minimization of Bias

Selection of subjects, treatment of subjects, and evaluation of study data are potential sources of bias. Methods incorporated in the study design to minimize potential bias include (but are not limited to):

- Subjects will be screened to confirm eligibility for enrollment with defined inclusion/exclusion criteria prior to enrollment.

- Subjects will be randomized 1:1 to the continuous monitoring arm vs control arm
- Demographics and medical history will be collected at baseline in order to later assess possible characteristics that may influence endpoints.
- To ensure a widespread distribution of data from study sites, no more than 10% of total expected enrollments (50 subjects) may come from a single site.
- It is recommended that all patients at an investigational site with a recent ischemic stroke of presumed known origin be screened for the study.
- All study site and Medtronic personnel will be trained using standardized training materials.
- Regular monitoring visits will be conducted to verify adherence to the Clinical Investigation Plan and source data.
- An independent clinical events committee (CEC) will be utilized to regularly review and adjudicate reported adverse events and recurrent stroke/TIAs.
- An independent committee will adjudicate AF episodes. The committee will be comprised of individuals experienced with identifying AF will review the device EGM records to ensure AF was appropriately identified.

In summary, potential sources of bias that may be encountered in this clinical study have been considered and minimized by careful study design.

5.2. Duration

The enrollment period is expected to start in early 2016 and will take approximately 42 months. Subjects will be followed for 3 years, until end of device life or until official study closure, whichever occurs first. Therefore, the expected study duration from first enrollment to official study closure (final report) is approximately 6.5 years. The duration of individual subject participation will vary based on timing of site activation and their enrollment; however, at a minimum, participation of an individual subject will be for 3 years, until end of device life or until study closure, whichever occurs first.

5.3. Rationale

The results of the Stroke AF clinical study will provide evidence that could impact the treatment and long-term prognosis for patients classified as having an IS of presumed known origin by demonstrating the value of long term monitoring for AF in this patient population. While current guidelines do recommend short term (up to 30 days) cardiac monitoring in patients after stroke, evidence generated in the Stroke AF study could influence the guidelines with regards to long term (greater than 30 day) ICM use post IS.

6. Product Description

6.1. General

The Stroke AF study will be conducted using the components of the Medtronic Reveal LINQ system.

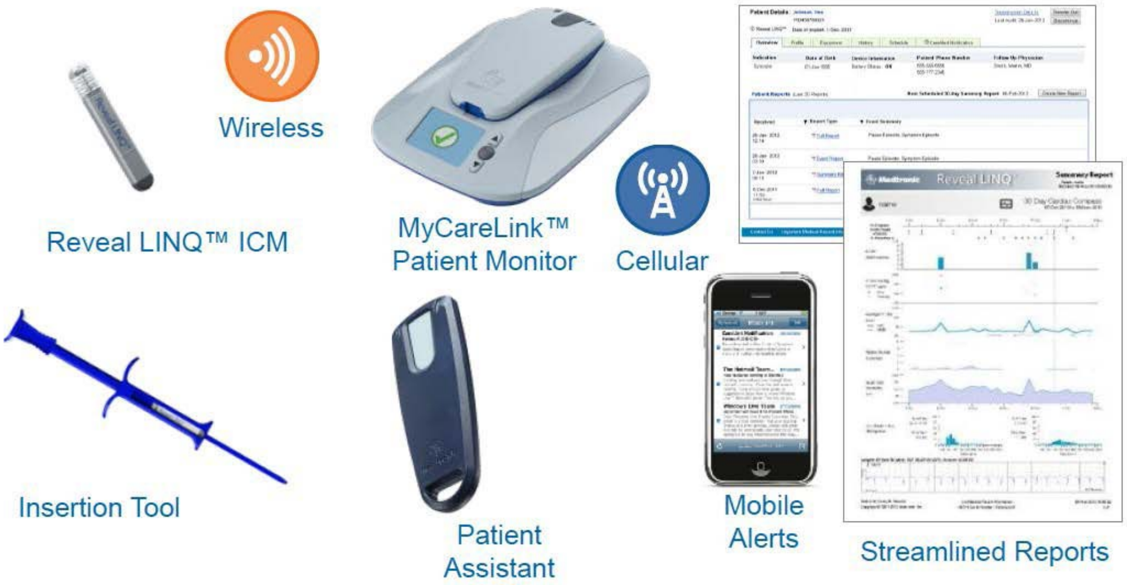

6.2. Reveal LINQ System

The Reveal LINQ system consists of 4 main components.

The Medtronic Reveal LINQ ICM is a programmable device that continuously monitors a patient's ECG and other physiological parameters. The device records cardiac information in response to automatically detected arrhythmias and patient activation.

The device is designed to record the occurrence of an arrhythmia in a patient automatically. Arrhythmias may be classified as tachyarrhythmia, brady arrhythmia, pause, atrial tachyarrhythmia, or atrial fibrillation. In addition, while experiencing or immediately after a symptomatic event, the patient can activate the device to record their cardiac rhythm.

Reveal LINQ ICM: is a small, leadless device that is inserted under the skin, in the chest. The device uses 2 electrodes on the body of the device to monitor the patient's subcutaneous ECG continuously. The device memory can store up to 27 min of ECG recordings from automatically detected arrhythmias and up to 30 min of ECG recordings from patient-activated episodes. The system provides 3 options for segmenting the patient-activated episode storage: up to four 7.5 min recordings, up to three 10 min recordings, or up to two 15 min recordings. Arrhythmia detection parameters are set to pending automatically, based on patient information entered on the programmer during pre-insertion device setup: the patient's Date of Birth and the clinician's Reason for Monitoring the patient. Arrhythmia detection parameters can also be programmed manually by the clinician.

Medtronic Programmer: is used to set up the device to detect arrhythmias. It also allows the user to view, save, or print the information stored by the device.

Patient Assistant: is a hand-held, battery-operated telemetry device that enables the patient to activate the recording of cardiac information in the Reveal LINQ ICM while experiencing or immediately after a symptomatic event. The clinician uses the recorded information to determine if the symptoms were associated with a cardiac event.

MyCareLink Patient Monitor: is used by patients to gather information automatically from their inserted device and communicate the information to their physician. The inserted device communicates wirelessly with this monitor which then transmits the information over a cellular telephone connection to the Medtronic CareLink Network. This daily wireless audit transmission is scheduled by the clinic and is usually set for a time when the patient is asleep. At other times, if requested to do so by their physician or clinic, the patient can use their monitor to perform a manual device interrogation to gather information from their inserted device and communicate it to their physician. Patient interaction with their monitor includes the initial setup procedure, performing physician-requested data gathering, and responding to physician-specified notifications on the monitor screen. Refer to the literature that is included with the MyCareLink Patient Monitor for connection and usage information.

Additional information related to the Reveal LINQ ICM, including indications, contraindications, warnings and precautions can be found in the respective clinician manual.

Table 1: Reveal LINQ System Component Information

Model Number	Component (Manufacturer)	Investigational or Market-released in the United States
LNQ11 or other commercial model	Reveal LINQ Insertable Cardiac Monitor	Market- released
2090 or other commercial model	Medtronic Programmer	Market- released
960000 or other commercial model	Patient Assistant	Market- released
24950 or other commercial model	MyCareLink Patient Monitor	Market- released

6.3. Intended Population

The Reveal LINQ system is being used in accordance with the indications for the device. The Reveal ICM is indicated for:

- Individuals with clinical syndromes or situations at increased risk of cardiac arrhythmias.
- Individuals who experience transient symptoms such as dizziness, palpitation, syncope, and chest pain that may suggest a cardiac arrhythmia.

6.4. Packaging

Labeling and package for all products used in this study will follow the local regulatory requirements. Labeling and reference/technical manuals for the Reveal LINQ ICM will be provided under separate cover. Labeling for all other market approved system components can be found with each package insert.

6.5. Equipment

The following equipment must be available at each site to support study activities:

- Computer with high speed internet and Windows Internet Explorer for data entry (Version 6, 8 or future compatible version) or other compatible browser
- Medtronic Market Released Programmer

The maintenance and calibration of the equipment used for this study will be assessed by the study site. Programmer calibration will not be monitored by the clinical investigation team, but will be maintained by Medtronic field representatives as per standard practice.

6.6. Product Use

Instructions for use of the devices used in this study are provided in their respective manuals.

6.7. Product Training Requirements

6.7.1. Investigator / Investigation Site Selection

All clinical investigators managing the subject's condition during the study must be qualified practitioners experienced in the diagnosis and medical treatment of subjects with ischemic stroke. All physicians performing the insertion procedure must be experienced and/or trained in the handling of insertable cardiac monitoring devices.

The role of the principal investigator is to implement and manage the day-to-day conduct of the clinical investigation as well as ensure data integrity and the rights, safety and well-being of the subjects involved in the clinical investigation.

The principal investigator shall:

- Be qualified by education, training, and experience to assume responsibility for the proper conduct of the clinical investigation
- Be experienced in the diagnoses and treatment of stroke patients
- Disclosure of potential conflicts of interest that interfere with the conduct of the clinical investigation or interpretation of results

The principal investigator shall be able to demonstrate that the proposed investigational site:

- Has the required number of eligible subjects needed within the recruitment period
- Has one or more qualified investigators, a qualified investigational site team and adequate facilities for the foreseen duration of the clinical investigation

Site personnel training will be completed prior to participation in this clinical study.

6.7.2. Site Activation

During the activation process (prior to subject enrollment), Medtronic will train site personnel on the clinical investigation plan, relevant standards and regulations, if needed, informed consent, and on data collection and reporting tools. If new members join the study site team, they will receive training on the applicable clinical study requirements relevant to their role before contributing to the clinical study.

Prior to performing study related activities, all local regulatory requirements shall be fulfilled, including, but not limited to the following:

- Ethics Committee approval of the current version of the CIP and Patient Informed Consent.

- Fully executed Clinical Trial Agreement (CTA)
- Curriculum Vitae (CV) of investigators and key members of the investigation site team (as required by local law)
- Documentation of delegated tasks
- Documentation of study training

Additional requirements imposed by the Ethics Committee and regulatory authority shall be followed, if appropriate.

In addition, all participating site staff must be trained on the current version of the CIP and must be delegated by the principal investigator to perform study related activities.

Medtronic will provide each study site with documentation of study site/investigator readiness; this letter must be received prior to subject enrollment.

6.8. Product Storage

The Reveal LINQ ICM is commercially available and will be managed in a manner consistent with other market-released product.

6.9. Product Return

All explanted devices (devices or leads or activators, etc.) should be returned to Medtronic for analysis when permissible by local laws and regulations. To receive a Returned Product Mailer Kit, please contact your local Medtronic field personnel.

7. Selection of Subjects

7.1. Study Population

Subjects will be screened to ensure they meet all of the inclusion and none of the exclusion criteria prior to study enrollment.

7.2. Subject Enrollment

Ethics Committee approval of the Stroke AF Study Clinical Investigation Plan and Informed Consent Form must be obtained prior to enrolling patients in the study. Subjects are considered enrolled in the study upon signing the informed consent. Informed consent must be obtained prior to performing any study related procedures. The subject must be enrolled within 10 days of the qualifying stroke event.

7.3. Inclusion Criteria

Inclusion Criteria	Rationale
Subject has had an ischemic stroke believed to be due to small vessel disease, large vessel cervical or intracranial atherosclerosis within the past 10 days.	Study will be evaluated in an already indicated patient population where evidence demonstrating benefit is needed.
Subject is willing and able to undergo study requirements and expected to be geographically stable during study follow-up.	Ensure ascertainment of data required for clinical evaluation.
Subject is 60 years of age or older, or age 50 to 59 years plus a documented medical history of at least one of the following additional risk factors for stroke:<br>• Congestive heart failure<br>• Hypertension (Systolic Blood Pressure > 140)<br>• Diabetes Mellitus<br>• Prior Stroke (>90 days ago, other than study qualifying index event)<br>• Vascular disease (e.g. coronary artery disease, heart attack, peripheral artery disease and complex aortic plaque)	Ensure the enrollment of patients who are at highest risk.

7.4. Exclusion Criteria

Exclusion Criteria	Rationale
Subject has had a cryptogenic stroke	This study aims to fill gaps in knowledge around the rate of AF in patients with ischemic stroke of presumed known origin. Data on the incidence rate of AF in these patients already exists.
Subject has had a cardioembolic stroke	Patients with known cardioembolic stroke are already being treated with OAC.
Subject has untreated hyperthyroidism	Avoid possible confounding factors relative to patient's medical condition.
Subject has had a recent myocardial infarction <1 month of stroke	Avoid possible confounding factors relative to patient's medical condition.
Subject has had a recent cardiac surgery (e.g. coronary artery bypass surgery (CABG)) <1 month of stroke	Avoid possible confounding factors relative to patient's medical condition.
Subject has a mechanical heart valve	Avoid possible confounding factors relative to patient's medical condition.

Exclusion Criteria	Rationale
Subject has valvular disease requiring immediate surgical intervention	Avoid possible confounding factors relative to patient's medical condition.
Subject has documented prior history of atrial fibrillation or atrial flutter	AF has already been diagnosed in these patients.
Subject has permanent indication for oral anticoagulation	Generally patients begin an OAC regimen once diagnosed with AF, these patients are already on OAC.
Subject has permanent contraindication to oral anticoagulation such that detection of AF would not change medical management, based on enrolling investigators judgment	The detection of AF would not result in a change to the patients' medical management.
Subject is enrolled in a concurrent study that may confound the results of this study. Co-enrollment in any concurrent clinical study (including registries) requires approval of the study manager or designee.	Standard exclusion criteria.
Subject's life expectancy is less than 1 year	Standard exclusion criteria to ensure study cohort is expected to survive to the time of endpoint evaluation.
Subject is pregnant	Standard exclusion criteria.
Subject has or is indicated for implant with a pacemaker, ICD, CRT, or an implantable hemodynamic monitor	Avoid possible confounding factors relative to patients' cardiac condition.
Subject with a medical condition that precludes the patient from participation in the opinion of the investigator	Standard exclusion criteria to apply medical discretion in subject selection

8. Study Procedures

Prior to performing study related procedures, all sites must have Ethics Board/IRB/MEC as well as documentation from Medtronic of site readiness.

8.1. Schedule of Events

Clinical data will be collected at baseline, 1, 6, and 12 months post-randomization, and at recurring 6 month intervals after that until study exit. Additionally, subjects randomized to the continuous monitoring arm will have data collected at Reveal LINQ ICM insertion, and 3, and 9 months (Remote CareLink Transmission).

Data collection requirements are summarized in the table below.

Table 2: Data Collection and Study Procedures

Study Procedure	Baseline	LINQ Insertion*	1, 6 and 12 Month Follow-up	3, 9 Months Remote Follow-up*	Recurring 6 Month Follow-up (through study exit)	Unscheduled Follow-up	Stroke/TIA Recurrence
Informed Consent	X						
Inclusion/Exclusion Assessment	X						
Randomization	X						
Demographics, Medical History	X						
Cardiovascular Medications	X		X		X		
TOAST1	X						X
NIH Stroke Scale	X						X
Modified Rankin Scale	X		X				X
EQ-5D	X		X				
Blood Draw	X (if available)						
System & Procedure Information*		X*					
Full Reveal LINQ Interrogation or CareLink Transmission*		X*	X*	X*	X*	X* (if available)	X* (if available)
AF Assessment			X		X	X	X
Symptom Assessment			X		X	X	
Stroke/TIA Assessment			X		X	X	
Adverse Events	As they occur						
Device Deficiencies							
System Modifications*							
Crossover							
Study Deviations							
Subject Exits							
Death							

[1]*Trial of Org 10172 in Acute Stroke Treatment*
** Subjects with a Reveal LINQ ICM only*

8.2. Subject Consent

Patient informed consent (PIC) is defined as a legally effective documented confirmation of a subject's (or their legally authorized representative) voluntary agreement to participate in a particular clinical study after information has been given to the subject on all aspects of the clinical study that are relevant to the subject's decision to participate. This process includes obtaining a Patient Informed Consent Form and an Authorization to Use and Disclose Personal Health Information/Research Authorization/and other privacy language as required by law that has been approved by the study site's Ethics Committee and signed and dated by the subject (or their legally authorized representative). A subject may only consent after information has been given to the subject on all aspects of the clinical investigation that are relevant to the subject's decision to participate. Informed consent may be given by the legally authorized representative only if a subject is unable to make the decision to participate in a clinical investigation. In such cases, the subject shall also be informed about the clinical investigation within his/her ability to understand.

Investigators shall consider all subjects who meet eligibility requirements for study participation to avoid any bias in the subject population. Prior to enrolling subjects, each site's Ethics Committee will be required to approve the CIP, PIC Form, and any other written study information to be provided to the subjects. The document(s) must be controlled (i.e. versioned and/or dated) to ensure it is clear which version(s) were approved by the Ethics Committee. Any adaptation of the sample Consent Form must be reviewed and approved by Medtronic and the Ethics Committee reviewing the application prior to enrolling subjects.

The investigator must notify the subject (or their legally-authorized representative) of any significant new findings about the study that become available during the course of the study which are pertinent to the safety and well-being of the subject, as this could impact a subject's willingness to participate in the study. If relevant, approval may be requested from subjects to confirm their continued participation.

Refer to Appendix C for the sample Patient Informed Consent Form(s).

Prior to initiation of any study-specific procedures, patient informed consent must be obtained from the subject (or their legally authorized representative). Likewise, privacy or health information protection regulation may require subjects to sign additional forms to authorize sites to submit subject information to the study sponsor. The informed consent process must be conducted by the principal investigator or an authorized designee, and the Patient Consent Form and Authorization to Use and Disclose Personal Health Information/Research Authorization/other privacy language as required by law must be given to the subject (or their legally authorized representative) in a language he/she is able to read and understand. The process of patient informed consent must be conducted without using coercion or undue improper influence on or inducement of the subject to participate by the investigator or other site personnel. The informed consent process shall not waive or appear to waive subject's legal rights.

The language used shall be as non-technical as possible and must be understandable to the subject and the impartial witness, where applicable.

The subject must have ample time and opportunity to read and understand the informed consent form, to inquire about details of the study, and to decide whether or not to participate in the clinical study. All questions about the study should be answered to the satisfaction of the subject.

When the subject decides to participate in the clinical study, the PIC must be signed and personally dated by the subject and investigator or authorized designee, as required by the Patient Consent Form.

A copy of the Patient Consent Form and the Authorization to Use and Disclose Personal Health Information/Research Authorization/other privacy language, signed and dated as required by law, must be provided to the subject.

If the PIC is obtained the same day the subject begins participating in study-related procedures, it must be documented in the subject's case history that consent was obtained prior to participation in any study-related procedures. It is best practice for the informed consent process to be documented in the subject's case history, regardless of circumstance.

In the event the subject cannot read and/or write, witnessed (impartial third party) patient informed consent will be allowed, provided detailed documentation of the process is recorded in the subject's case history and the witness signs and dates the patient informed consent. The Consent Form should document the method used for communication with the prospective subject and the specific means by which the prospective subject communicated agreement to participate in the study.

The Patient Consent Form and Authorization to Use and Disclose Personal Health Information/Research Authorization/other privacy language as required by law must be available for monitoring and auditing. Any Medtronic Field personnel who support the Reveal LINQ ICM insertion procedure must be able to review the subject's original signed and dated Consent Form and verify its completeness prior to proceeding with the insertion. In the event the Medtronic Field personnel identify patient informed consent as being incomplete, the study procedure will not be allowed to occur until the consent of the subject can be adequately and appropriately obtained.

When a patient and the principal investigator or authorized designee, as required have personally signed and dated the Consent Form, the patient is considered a subject enrolled in the study.

The date the subject signed the Consent Form and data protection authorization must be documented in the subject's medical records.

8.3. Randomization and Treatment Assignment

Subjects are eligible for randomization assignment after study enrollment and verification of eligibility criteria. Enrollment, randomization and insertion of the Reveal LINQ ICM must occur within 10 days of the qualifying stroke event.

Subjects will be randomized in a 1:1 fashion to the continuous monitoring arm (Reveal LINQ ICM) or control arm (standard of care) using the RDC electronic system. Randomization schedules will be created by a statistician and randomization assignments will be automatically populated on the Randomization e-CRF after subject consent and eligibility verification have been entered in RDC. The randomization schedule will be stratified by study site to ensure 1:1 randomization within each study site.

Once subjects are assigned to a study arm (continuous monitoring or control), they are considered randomized. All randomized subjects should be encouraged to comply with the Stroke AF study procedures until study closure and every effort should be made to avoid crossover. Statistical analysis will be done on an intention to treat basis unless otherwise specified. Therefore, once a subject is assigned to an arm, he/she will remain in this arm and all efforts will be made to provide the optimal therapy specified per the assigned treatment arm. In case this is clinically or technically not feasible, the intention to treat is assured. A per-protocol analysis cohort will also be defined within the statistical analysis plan (SAP) and additional supportive analyses of the objectives may be performed in this cohort.

8.4. Procedures and Data Collection

8.4.1. Baseline

The baseline visit can be a standalone visit or can be performed on the same day, but not later than the insertion procedure.

The following information is required to be collected at the baseline visit:

- Informed Consent
- Inclusion/Exclusion Assessment
- Randomization
- Subject Demographics
- Medical History, including but not limited to cardiovascular history, and details about the qualifying stroke event (if available)
- Cardiovascular Medications
- TOAST Stroke Classification
- National Institute of Health (NIH) Stroke Scale
- Modified Rankin Scale
- EQ-5D™
- Blood Draw

- Five blood tubes (one plasma, three serum and one whole blood) will be collected, processed, and sent to the central laboratory for analysis of biomarkers that are considered potential predictors of identifying patients who may at high risk for AF.
  - One 6 mL tube of blood will be collected and processed for plasma for B-type Natriuretic Peptide (BNP) analysis. The residual from the tube will be kept in long-term storage for future analysis.
  - Two 5 mL tubes of blood will be collected and processed for serum for Troponin-I, C-reactive protein (CRP), and thyroid-stimulating hormone (TSH) testing. The residuals from these three tubes will be kept in long-term storage for future analysis.
  - One 5 mL tube of blood will be collected, processed for serum and kept in long-term storage for future analysis.
  - One 4 mL tube of whole blood will be collected for genetic testing such as genotyping single nucleotide polymorphisms (SNP).
- Specifics regarding the acquisition of these specimens, necessary supplies, and shipping information, under separate cover, will be provided to all study centers by the central laboratory.
- If a study center does not have the ability to perform the blood draw per the CIP or if the patient declines, a study deviation is not required.

8.4.2. Reveal LINQ ICM Insertion

The insertion procedure must occur within 10 days of the qualifying stroke event and after randomization. The insertion will be performed in accordance with the hospital's standard insertion practice and in accordance with the Medtronic Reveal LINQ ICM insertion instructions which are found within the Reveal LINQ ICM Clinician manual. The following information is required to be collected at the insertion visit:

- System and Procedure Information

  System and procedure details will be collected including, but not limited to procedure date, device serial number, location of insertion procedure, location/orientation of inserted device, antibiotic use, and closure method.
- Device Programming/Interrogation

  Programming requirements for the Reveal LINQ ICM are outlined in Table 6 below. A full device interrogation will be collected after final programming. Programming changes will be allowed only when clinically necessary. All deviations from the programming guidelines (regardless of when they occur) must be documented on a Study Deviation e-CRF, including the rationale for the deviation.

Table 3: Reveal LINQ ICM Programming

Parameter	Required Setting
Reason for Monitoring	Cryptogenic Stroke
AF Detection Sensitivity	Balanced Sensitivity
Ectopy Rejection	Aggressive
AT/AF Recording Threshold	All Episodes
Type of AT/AF Detection	AF Only
Wireless Data Priority	Tachy, Pause, Brady
Tachy Detection	ON
Brady Detection	ON
Pause Detection	ON

The subject will be instructed about the use of the Medtronic MyCareLink Monitor and the Patient Assistant.

If the subject does not have a Reveal LINQ ICM device in the body at the conclusion of the procedure, the subject should remain in the study and the follow-up schedule remains unchanged. For these patients, CareLink transmissions at 3 and 9 months are not required.

8.4.3. Scheduled Follow-up Visits/CareLink Transmissions

After receiving notice of randomization assignment, Medtronic will provide the target dates and windows for each scheduled visit/CareLink transmission to the site. Should a subject miss a visit/transmission or the visit/transmission fall outside the pre-specified window, a study deviation must be reported and the original follow-up schedule maintained for subsequent visits/transmissions.

Data analyses include follow-up visits/CareLink transmissions, regardless of whether the visit occurs within the window; therefore, a late visit is preferred over a missed visit. Follow-up visit/CareLink transmission windows are listed in the Table 7 below and are based on days post randomization.

Table 4: Follow-up Visit / CareLink Transmission Schedule

Study Follow-up Visit	Window (Calculated days post randomization)		
	Window Start	Target	Window End
1 Month	23	30	37
3 Month (CareLink Transmission)	76	91	106
6 Month	168	183	198
9 Month (CareLink Transmission)	259	274	289
12 Month (1 year)	365	365	421
18 Month	521	549	577
24 Month (2 year)	702	730	758
30 Month	887	915	943
36 Month (3 year)	1067	1095	1123

8.4.3.1. Follow-up Visits

The following information will be collected at during follow-up visits at 1, 6, and 12 months post-insertion, and at recurring 6 month intervals after that until study closure:

- Cardiovascular Medications
- Modified Rankin Scale (1, 6 and 12 month only)
- EQ-5D™ (1, 6 and 12 month only)
- Reveal LINQ Interrogation or remote CareLink transmission (subjects with a Reveal LINQ ICM only)
- AF Assessment
- Symptom Assessment
- Stroke/TIA Assessment

Data may be collected remotely for subjects unable to return to the clinic for the follow up visit.

Patients in the control arm should be monitored for cardiac arrhythmias per the study site standard of care. Source documentation (e.g. ECG, Holter, etc.) from any additional cardiac arrhythmia diagnostic testing or monitoring which detects AF must be sent to Medtronic.

8.4.3.2. Follow-up Visits / Remote CareLink Transmissions

Subjects are required to perform a manual CareLink transmission at 3, and 9 months following successful Reveal LINQ ICM insertion.

CareLink transmission data will be automatically transferred to the Medtronic Data Warehouse for CareLink. If unavailable, sites may be asked to submit CareLink reports by uploading them to a secure server or sending printed versions of the CareLink reports to Medtronic. Printouts only need to be provided to Medtronic upon request.

8.4.4. Unscheduled Follow-up Visits

An unscheduled visit is defined as any unplanned cardiology or neurology visit by the subject to the study site. Routine visits or other planned visits are not collected.

The following information is required to be collected at unscheduled follow-up visits:

- Reveal LINQ Interrogation (if available, subjects with a Reveal LINQ ICM only)
- AF Assessment
- Symptom Assessment
- Stroke/TIA Assessment
- Adverse Event e-CRF (if applicable)

8.4.5. Stroke/TIA Recurrence

Following the index event, a subject should be monitored for stroke recurrence. If there is a stroke/TIA recurrence, the following data is required to be collected:

- Details about the stroke event, such as type, size, and location.
- TOAST stroke classification
- National Institute of Health (NIH) Stroke Scale
- Modified Rankin Scale
- Reveal LINQ Interrogation (if available, subjects with a Reveal LINQ ICM only)
- AF Assessment
- Adverse Event e-CRF

8.4.6. System Modification

A system modification will be reported in the event the Reveal LINQ ICM requires invasive modification (e.g., explant, replacement, repositioning).

- If the modification consists of repositioning or replacement, the follow-up schedule for the subject will remain unchanged.

- If the Reveal LINQ ICM is explanted, the subject should continue participation in this study and a crossover e-CRF is required. If the subject receives implant of another Medtronic device (IPG, ICD or CRT) reference Table 8 for recommended programming parameters for these devices. The other parameters for these devices are to be programmed based on the investigator's opinion.

Table 5: Recommended Programming Parameters for Medtronic IPG, ICD or CRT Devices

Parameter	Recommended Setting
Atrial preference pacing (APP)	OFF
Atrial rate stabilization (ARS)	OFF
Post-mode switch overdrive pacing (PMOP)	OFF
Atrial Anti-tachycardia pacing (ATP)	OFF
AT/AF detection and EGM collection	Nominal

The following will be collected in the event of a system modification:

- Reason for modification
- System/Procedure Information
- Pre-modification: device interrogation
- Post-modification: device interrogation (if modification includes a Medtronic device)

All explanted product should be returned to Medtronic for analysis when permissible by local laws and regulations.

8.4.7. Study Exit

A study exit e-CRF is required for all subjects except in the case of death. Prior to exiting a subject from the study, it is recommended to follow the subject until all ongoing system and/or procedure related AEs are resolved or unresolved with no further actions planned. Following exit, subjects should continue to receive standard medical care. Upon exiting from the study, no further study data will be collected or study visits will occur for the subject. All data available through the time of the subject's exit will be used for analysis.

Subjects are urged to remain in the study as long as possible but may be exited from the study for any of the following situations:

- Subject lost to follow-up
- Subject did not meet inclusion/exclusion criteria and not randomized
- Subject not randomized
- Subject did not provide consent or data use protection authorization

- Subject chooses to withdraw (e.g., consent withdrawal, relocation to another geographic location)
- Investigator deems withdrawal necessary (e.g., medically justified, inclusion/exclusion criteria not met, failure of subject to maintain adequate study compliance)
- Subject with a Reveal LINQ ICM that has reached end of device life (continuous monitoring patients only)
- Subject has completed 3 years of follow-up
- Study Closure

The following information is required to be collected at study exit on the study exit e-CRF:

- Date of exit
- Reason for exit
- Reveal LINQ Interrogation (if available, subjects with a Reveal LINQ ICM inserted only)
- AF Assessment (except in the case of lost to follow-up or death)
- Stroke/TIA Assessment (except in the case of lost to follow-up or death)

In the case that the subject is determined to be lost to follow-up, details of a minimum of two attempts and the method of attempt (e.g., one letter and one phone record or two letters) to contact the subject must be documented. In addition, follow the regulations set forth by the governing Ethics Committee.

8.5. Assessment of Safety

Timely, accurate, and complete reporting and analysis of safety information for clinical studies are crucial for the protection of subjects. Reporting and analysis of safety data are mandated by regulatory authorities worldwide. Medtronic has established procedures in conformity with worldwide regulatory requirements to ensure appropriate reporting of safety information. This study is conducted in accordance with these procedures and regulations.

Since the safety reporting requirements and classification systems vary for each regulatory agency, requirements from all geographies are taken into account for the collection and reporting of safety information.

8.6. Recording Data

Data will be collected via electronic Case Report Forms (e-CRFs), Reveal LINQ ICM device interrogation files, and Medtronic CareLink transmissions. A web-based application tool, Remote Data Capture (RDC) will be used for data entry of e-CRFs. The investigator is responsible for the preparation (review and signature) of the e-CRF.

8.7. Deviation Handling

A study deviation is defined as an event within a study that did not occur according to the Clinical Investigation Plan or the Clinical Trial Agreement.

Prior approval by Medtronic is expected in situations where the investigator anticipates, contemplates, or makes a conscious decision to deviate. Prior approval is not required when a deviation is necessary to protect the safety, rights or well-being of a subject in an emergency or in unforeseen situations beyond the investigator's control (e.g. subject failure to attend scheduled follow-up visits, inadvertent loss of data due to computer malfunction, inability to perform required procedures due to subject illness).

For medically justifiable conditions which preempt a subject's ability to complete a study-required procedure, it may be permitted to report only one deviation which will apply to all visits going forward. This may also apply for other unforeseen situations (e.g. the subject permanently refuses to complete a study required procedure and the data will not contribute to the primary end point analysis). However, prior approval from Medtronic is required for such situations.

All study deviations must be reported on the Study Deviation e-CRF regardless of whether medically justifiable (e.g. crossover), pre-approved by Medtronic, an inadvertent occurrence, or taken to protect the subject in an emergency. The deviation description must be recorded with an explanation for the deviation. In the occurrence of a corrupted device interrogation file, Medtronic may request a deviation to document that a readable interrogation file is unavailable.

In the event the deviation involves a failure to obtain a subject's consent, or is made to protect the life or physical well-being of a subject in an emergency, the deviation must be reported to the Ethics Committee as well as Medtronic within five (5) working days. Reporting of all other study deviations should comply with Ethics Committee policies and/or local laws and must be reported to Medtronic as soon as possible upon the site becoming aware of the deviation. Reporting of deviations must comply with Ethics Committee policies, local laws, and/or regulatory agency requirements. Refer to Investigator Reports, Table 12 for geography-specific deviation reporting requirements and timeframes for reporting to Medtronic and/or regulatory bodies.

Medtronic is responsible for analyzing deviations, assessing their significance, and identifying any additional corrective and/or preventive actions (e.g. amend the Clinical Investigation Plan, conduct additional training, terminate the investigation). Repetitive or serious investigator compliance issues may result in initiation of a corrective action plan with the investigator and site, and in some cases, necessitate suspending enrollment until the problem is resolved or ultimately terminating the investigator's participation in the study. Medtronic will provide site-specific reports to investigators summarizing information on deviations that occurred at the investigational site on a periodic basis.

8.8. Subject Withdrawal or Discontinuation

Subjects will be followed until study closure.

9. Risks and Benefits

Medtronic follows rigorous Quality Assurance and Control procedures throughout the life of a product, from the business analysis phase through development, market release, and post-market surveillance.

The Stroke AF study system and components are commercially released and used in accordance with their approved labeling. The safety and clinical performance of these devices has been demonstrated through previous pre-clinical testing and previous clinical studies. There are no incremental risks introduced to the subject as a result of participation in the Stroke AF study. Devices should be handled in accordance to the Clinical Manual.

For subjects with a Reveal LINQ ICM inserted, Risks and Risk Mitigations to the subject identified below are consistent with those outlined in the device manual. There may be other discomforts and risks related to the device system and/or this study that are not foreseen at this time.

9.1. Potential Risks

Possible risks associated with the Reveal LINQ ICM include but are not limited to the following:

- Risks associated with a minor surgical procedure
- Slight risk of infection
- Sensitivity to the device

9.2. Potential Benefits

The Reveal LINQ ICM may offer no benefit. The potential benefits of having the Reveal LINQ ICM include more intensive follow-up treatment and frequent device transmissions and/or continuous monitoring enabling more precise detection of AF. This better precision to detect AF may lead to clinicians having better data to quantify AF burden. The information gained from this study could result in the improved detection and management of atrial fibrillation. Additionally, information collected from this study may assist in the design of new product(s)/therapy(ies) and/or instructions for use. The System may not offer a direct clinical benefit to study subjects. The information collected from this study may assist in the design of new product(s)/therapy(ies) and/or instructions for use.

9.3. Risk-Benefit Rationale

The potential risks associated with the Reveal LINQ ICM were identified and have been successfully mitigated. Any potential risks associated with this study are further minimized by selecting qualified investigators and training study personnel on the Clinical Investigation Plan.

In addition, investigators will be actively involved in the insertion and follow-up of the subjects implanted with the Reveal LINQ system.

Risks will be minimized by careful assessment of each subject prior to, during, and after insertion of the Reveal LINQ ICM. Prior to insertion, it is recommended subjects undergo a complete cardiac evaluation.

After insertion, subjects in the Stroke AF clinical study will be followed at regular intervals. At each protocol required follow-up, the investigator must interrogate the Reveal LINQ ICM and assess for any adverse events.

- Risks associated with a minor surgical procedure
- Slight risk of infection
- Sensitivity to the device

10. Adverse Events and Device Deficiencies

All reported Adverse Events (AEs) must be followed until resolved, ongoing without further actions to be taken, the subject exits the study or until study closure, whichever occurs first. In the event that a subject is exited from the study prior to study closure, all efforts should be made to continue following the subject until all ongoing AEs are resolved or they are ongoing without further actions to be taken.

10.1. Adverse Event Assessment

10.1.1. Adverse Events

All Reveal LINQ ICM system/component-related, Reveal LINQ ICM procedure related, cardiovascular related AEs, and all serious adverse events leading to death will be collected throughout the study duration, starting at the time of signing the consent form.

Reporting of these events to Medtronic will occur on an Adverse Event (AE) e-CRF, including a description of AE, date of onset of AE, date of awareness of site, treatment, resolution, assessment of both the seriousness and the relatedness to the investigational device. Each AE must be recorded on a separate AE Form. Subject deaths are also required to be reported. Refer to Section 10.5 for Subject Death collection and reporting requirements.

Documented pre-existing conditions are not considered AEs unless the nature or severity of the condition has worsened. Additionally, detected arrhythmias (including AF) for which no action is taken are not reportable AEs. Unavoidable Adverse Events, listed in Table 9 need not be reported unless the adverse event worsens or is present outside the stated timeframe post-insertion.

For AEs that require immediate reporting (see Table 11), initial reporting may be done by phone, fax, or on the e-CRF completing as much information as possible, with missing or follow-up information provided as soon as it becomes available. The AE e-CRF must be completed as soon as possible.

10.1.2. Device Deficiencies

Device deficiency (DD) information will be collected throughout the study and reported to Medtronic. Note that device deficiencies that result in an adverse device effect (ADE) to the subject should be captured as an Adverse Event only.

10.1.3. Processing Updates and Resolution

For any changes in status of a previously reported AE (i.e. change in actions taken, change in outcome, change in relatedness), information needs to be updated on or added to the original AE e-CRF. All reported adverse events must be followed until the adverse event has been resolved, is unresolved with no further actions planned, the subject exits the study or until study closure, whichever occurs first.

In the event that a subject is exited from the study prior to study closure, all efforts should be made to continue following the subject until all unresolved procedure or system related adverse events, as classified by the investigator, are resolved or they are unresolved with no further actions planned.

At the time of study exit, all AEs with an outcome of "Unresolved, further actions or treatment planned" must be reviewed and an update to the original AE e-CRF must be reported. At a minimum, if there are no changes to the description, relatedness, test and procedures or actions taken, the outcome must be updated to reflect "Unresolved at time of study closure."

10.2. Definitions/Classifications

10.2.1. Adverse Event and Device Deficiency Definitions

Where the definition indicates "device", it refers to any device used in the study.

Table 6: Adverse Event Definitions

General	
Adverse Event (AE)<br>(Adapted from ISO 14155:2011, 3.2)	Any untoward medical occurrence, unintended disease or injury, or untoward clinical signs (including abnormal laboratory findings) in subjects, users or other persons, whether or not related to the investigational medical device<br><br>NOTE 1: This definition includes events related to the investigational medical device or the comparator.<br><br>NOTE 2: This definition includes events related to the procedures involved.
Adverse Device Effect (ADE)<br>(ISO 14155:2011, 3.1)	Adverse event related to the use of an investigational medical device<br><br>NOTE 1: This definition includes adverse events resulting from insufficient or inadequate instructions for use, deployment, implantation, installation, or operation, or any malfunction of the investigational medical device.<br><br>NOTE 2: This definition includes any event resulting from an error use or from intentional misuse of the investigational medical device.
Device Deficiency (DD)<br>(ISO 14155:2011, 3.15)	Inadequacy of a medical device with respect to its identity, quality, durability, reliability, safety or performance.<br><br>NOTE: Device deficiencies include malfunctions, use errors and inadequate labeling
**Relatedness**	
Reveal LINQ ICM Procedure Related	An adverse event that occurs that is directly related to the implantation or modification of the Reveal LINQ system.
Reveal LINQ ICM System Related	An adverse event that results from the presence or performance (intended or otherwise) of any component of the system (including: Reveal LINQ ICM, Programmer, Patient Assistant, and MyCareLink Patient Monitor).
Cardiovascular Related	An adverse event relating to the heart and the blood vessels or the circulation.
**Seriousness**	

Complication	An adverse event that results in death, involves any termination of significant device function, or requires invasive intervention.<br><br>Non-invasive: when applied to a diagnostic device or procedure, means one that does not by design or intention:<br>• Penetrate or pierce the skin or mucous membranes of the body, the ocular cavity or the urethra or<br>• Enter the ear beyond the external auditory canal, the nose beyond the nares, the mouth beyond the pharynx, the anal canal beyond the rectum, or the vagina beyond the cervical os<br><br>For purposes of this part, blood sampling that involves simple venipuncture is considered non-invasive, and the use of surplus samples of body fluid or tissue that are left over from samples taken for non-investigational purposes is also considered non-invasive.
Observation	Any adverse event that is not a complication.<br><br>Note 1: Only system or procedure related AEs will be classified as Complication or Observation.
Serious Adverse Event (SAE) (Adapted from ISO 14155:2011, 3.37)	Adverse event that<br>a) led to death,<br>b) led to serious deterioration in the health of the subject, that either resulted in<br>5. a life-threatening illness or injury, or<br>6. a permanent impairment of a body structure or a body function, or<br>7. in-patient or prolonged hospitalization, or<br>8. medical or surgical intervention to prevent life-threatening illness or injury or permanent impairment to a body structure or a body function,<br>c) led to fetal distress, fetal death or a congenital abnormality or birth defect<br><br>NOTE 1: Planned hospitalization for a pre-existing condition, or a procedure required by the CIP, without serious deterioration in health, is not considered a serious adverse event.
**Other**	
Hospitalization	A therapeutic inpatient hospitalization (excludes outpatient and emergency room visits) lasting greater than or equal to 24 hours.
Stroke	Hemorrhagic or Ischemic stroke with rapid onset of a focal or global neurological deficit or other neurological signs/symptoms consistent with stroke.
Stroke Diagnosis	Diagnosis will be determined by the site's neurologist clinical evaluation and include clinical signs and symptoms consistent with focal or global neurological deficit in addition to one or more of the following:<br>• Neuroimaging procedure (CT, MR, or cerebral angiogram)<br>• Lumbar Puncture (i.e. subarachnoid hemorrhage)

<table>
<tr><td>Transient Ischemic Attack (TIA)</td><td>New focal neurological deficit with rapid symptom resolution (usually 1 – 2 hours), always within 24 hours without tissue injury (based on neuroimaging).</td></tr>
<tr><td>Unavoidable Adverse Event</td><td>An Adverse Event inherent to a surgical procedure that is expected to occur in all subjects for a projected duration according to the Investigator’s opinion, including, but not limited to:
<table>
<tr><th>Event Description</th><th>Timeframe (hours) from the Surgical Procedure</th></tr>
<tr><td>Anesthesia related nausea / vomiting</td><td>24</td></tr>
<tr><td>Low-grade fever (<100°F or 37.8°C)</td><td>48</td></tr>
<tr><td>Pocket site / Incisional pain</td><td>72</td></tr>
<tr><td>Mild to moderate bruising / ecchymosis</td><td>168</td></tr>
<tr><td>Sleep problems (insomnia)</td><td>72</td></tr>
<tr><td>Back pain related to laying on table</td><td>72</td></tr>
<tr><td>Shoulder pain/discomfort/stiffness related to shoulder immobilization during procedure</td><td>72</td></tr>
</table>
</td></tr>
</table>

10.2.2. Adverse Events and Deficiency Classification

All reported AEs and DDs will be reviewed by a Medtronic representative. AEs will be classified according to the definitions provided.

Upon receipt of AEs at Medtronic, a Medtronic representative will review the AE/DD for completeness and accuracy and when necessary will request clarification and/or additional information from the Investigator. Medtronic will utilize MedDRA, the Medical Dictionary for Regulatory Activities, to assign a MedDRA term for each adverse event based on the information provided by the investigator.

Regulatory reporting of AEs and DDs will be completed according to local regulatory requirements. Refer to Table 12 and Table 13 for a list of required investigator and Medtronic reporting requirements and timeframes. It is the responsibility of the investigator to abide by any additional AE reporting requirements stipulated by the Ethics Committee responsible for oversight of the study.

For emergency contact regarding a SAE, contact a clinical study representative immediately (refer to the study contact list provided in the site’s study documents binder/investigator site file or refer to the contact information provided on the title page).

Adverse Events and Deaths will be classified according to the standard definitions as outlined below:

Table 7: Adverse Event Classification Responsibilities

What is classified?	Who classifies?	Classification Parameters
Relatedness	Investigator	• Procedure Related<br>• System Related
	Sponsor	• Procedure Related<br>• System Related<br>• Cardiovascular Related
Seriousness	Investigator	SAE
	Sponsor	SAE, Complication or Observation (for all system or procedure related adverse events)
Diagnosis	Investigator	Based on presenting signs and symptoms and other supporting data
	Sponsor	MedDRA term assigned based on the data provided by Investigator
Death Classification	Investigator	Sudden Cardiac, Non-sudden Cardiac, Non-Cardiac, Unknown

An independent Clinical Events Committee (CEC) will at minimum conduct a medical review of all endpoint related events for subjects participating in the study. Additionally, the CEC will provide an adjudication of the death classification for all reported deaths.

10.3. Reporting of Adverse Events

Regulatory reporting of AEs/DDs will be completed according to local regulatory requirements. Refer to Table of Adverse Event Reporting Requirements for a list of required investigator reporting requirements and timeframes, and of required Medtronic reporting requirements and timeframes.

The investigator is required to report all SAEs to Medtronic immediately, and to the Ethics Committee per local requirements. Medtronic is also required to report these events to the local regulatory authority based on their requirements. It is the responsibility of the investigator to abide by any additional AE/DD reporting requirements stipulated by the Ethics Committee responsible for oversight of the study.

For AEs/DDs that require immediate reporting, initial reporting may be done by contacting the study sponsor per the sponsor contact information provided in this document.

Table 8: AE Reporting Requirements

**Serious Adverse Events (SAEs)**	
**Investigator submit to:**	
Medtronic	Submit in a timely manner after the investigator first learns of the event.
EC/IRB/MEC	Submit to EC/IRB/MEC per local reporting requirement.
Regulatory authorities	Submit to regulatory authority per local reporting requirement.
**Sponsor submit to:**	
Regulatory authorities	Submit to regulatory authority per local reporting requirement.
EC/IRB/MEC	Submit to EC/IRB/MEC per local reporting requirement.
**All other reportable Adverse Events (System, Procedure, Cardiovascular-Related)**	
**Investigator submit to:**	
Medtronic	Submit in a timely manner after the investigator first learns of the event.
EC/IRB/MEC	Submit to EC/IRB/MEC per local reporting requirement.
Regulatory authorities	Submit to regulatory authority per local reporting requirement.
**Sponsor submit to:**	
Regulatory authorities	Submit to regulatory authority per local reporting requirement.
EC/IRB/MEC	Submit to EC/IRB/MEC per local reporting requirement.
**Device Deficiencies**	
**Investigator submit to:**	
Medtronic	Submit in a timely manner after the investigator first learns of the event.
EC/IRB/MEC	Submit to EC/IRB/MEC per local reporting requirement.
Regulatory authorities	Submit to regulatory authority per local reporting requirement.

10.3.1. Subject Death

10.3.1.1. Death Data Collection

All subject deaths must be reported by the investigator to Medtronic using an AE e-CRF as soon as possible after the investigator first learns of the death. The AE leading to death must be reported and will document the death as the outcome.

For subjects with a Reveal LINQ ICM

In the event of a subject's death, the inserted device should be explanted and returned to Medtronic for analysis whenever possible. Local laws and procedures must be followed where applicable.

System Interrogation Data Recommendations:

- After the subject has died but prior to explant, the device shall be interrogated and a full summary interrogation (Interrogate All) performed when possible.

If the device is not interrogated, an explanation must be entered on the AE form. If any system component is returned to Medtronic, internal return product reporting systems may be used to gather additional information about the returned device/component.

A copy of the death certificate, if available and allowed by state/local law, should be sent to the Medtronic clinical study team. When a death occurs in a hospital, a copy of the death summary report and all relevant hospital records should be sent to the Medtronic clinical study team, if available. If an autopsy is conducted, the autopsy report should also be sent to the Medtronic clinical study team if available and allowed by state/local law. When the death occurs at a remote site, it is the investigative site's responsibility to attempt retrieval of information about the death. In summary, the following data will be collected:

- Date of death
- Detailed description of death
- Cause of death
- Relatedness to system and/or procedure (subjects with Reveal LINQ inserted)
- Device interrogation (if available, subjects with a Reveal LINQ ICM)
- Death summary/hospital records (if available and allowed by state/local law)
- Autopsy report (if available and allowed by state/local law)
- Death certificate (if available and/or allowed by state/local law)

10.3.1.2. Death Classification and Reporting

Sufficient information will be required in order to properly classify the subject's death. The Investigator shall classify each subject death per the following definitions:

Table 9: Subject Death Classification

Classification	Definition
Cardiac Death	A death directly related to the electrical or mechanical dysfunction of the heart
Sudden Cardiac Death (SCD)	Natural death due to cardiac causes, indicated by abrupt loss of consciousness within one hour of the onset of acute symptoms; preexisting heart disease may have been known to be present, but the time and mode of death are unexpected. If time of onset cannot be determined, SCD will alternatively be defined as any unexpected cardiac death occurring out of the hospital or in the emergency room as dead on arrival.
Non-sudden Cardiac Death	All cardiac deaths that are not classified as sudden deaths, including all cardiac deaths of hospitalized subjects on inotropic support.
Non-cardiac Death	A death not classified as a cardiac death
Unknown	There is insufficient or inadequate information to classify the death.

The Clinical Events Committee (CEC) will review deaths and provide a final adjudication of the primary cause of death and death classification.

Regulatory reporting of Subject Deaths will be completed according to local regulatory requirements. Refer to Table 12 and Table 13 for a list of required investigator and sponsor reporting requirements and timeframes.

10.4. Product Complaint Reporting

Product complaint reporting is applicable to any Medtronic market released product, regardless of subject's participation in a clinical study.

All devices used in the study are market-released, product complaint reporting is applicable. This includes when an AE is related to a market-released device during the study. The reporting of product complaints is not part of the clinical study and should be done in addition to the AE reporting requirements. Refer to local regulations for reporting requirements.

Product Complaint: Any written, electronic or oral communication that alleges deficiencies related to the identity, quality, durability, reliability, safety, effectiveness or performance of a medical device that has been placed on the market.

It is the responsibility of the investigator to report all product complaint(s) associated with a medical device distributed by Medtronic, regardless whether they are related to intended use, misuse or abuse of the product. Reporting must be done immediately and via the regular channels for market-released

products. The reporting of product complaints by the clinical study team must be done according to local Standard Operating Procedures.

Medtronic will notify the regulatory authorities (e.g. Competent Authority) as applicable for the following incidents immediately upon learning of them:

- Any malfunction or deterioration in the characteristics and/or performance of a device, as well as any inadequacy in the labeling or instructions for use which led or might have led to the death or serious deterioration in the state of health of a patient, user, or other person.
- Any technical or medical reason resulting in withdrawal of a device from the market by the manufacturer.
- A serious deterioration in the state of heath includes:
    - Life-threatening illness or injury
    - Permanent impairment of a body function or permanent damage to a body structure
    - In-patient or prolonged hospitalization
    - Medical or surgical intervention to prevent life-threatening illness or injury or permanent impairment to a body structure or a body function
    - Fetal distress, fetal death or a congenital abnormality or birth defect

11. Data Review Committees

The Stroke AF study will utilize a Steering Committee for study design and oversite, a CEC for the assessment of Adverse Events and recurrent stroke/TIA data, an Endpoint Adjudication Committee (the CEC may serve as the EAC) for an independent review of endpoint (AF) and a Publication Committee to manage study publications.

Committee membership rosters will be maintained at Medtronic and will be made available upon request

11.1. Clinical Events Committee (CEC) Review

At regular intervals, an independent Clinical Events Committee (CEC) will at minimum conduct a medical review (adjudicate) of reported arrhythmias including AF episodes/events as well as reported strokes/TIAs and deaths for subjects participating in the study.

The CEC will consist of a minimum of three (3) non-Medtronic employed physicians that are not participating investigators for the study, including a CEC chairperson. At least three CEC members must adjudicate, at a minimum, all deaths and endpoint related events. All other AEs may be adjudicated by at least one physician member of the CEC.

Medtronic personnel may facilitate and participate in a CEC meeting but will be non-voting members.

For adverse events and deaths reviewed by the CEC, Medtronic will provide the CEC with the Investigator's description and classification. The CEC is responsible for reviewing the Investigator's assessment and supportive documentation (when available), reviewing applicable definitions, and determining final classifications for all adjudication parameters. Additionally, the CEC will provide an adjudication of the death classification for all reported deaths including primary cause of death.

If the CEC disagrees with the investigator's classification of the event, the rationale will be provided to the investigator. If the investigator agrees with the CEC's adjudication, the case report form documenting the AE will be updated accordingly.

If the investigator does not agree with the CEC's adjudication classification, both determinations will be provided within the final report; however the CEC's adjudication will be used for data analysis. The disagreement will also be included in reporting to ethic committees and regulatory authorities, if required.

12. Statistical Design and Methods

Medtronic employees or designees will perform all statistical analyses. Additionally, a separate Statistical Analysis Plan (SAP) will be developed to further describe statistical methods, pre-specified data handling rules, and pre-specified analyses that will be included in study reports. Any change to the data analysis methods described in the CIP will require an amendment only if it changes a principal feature of the CIP. Any other change to the data analysis methods described in the CIP, and the justification for making the change, will be described in the clinical study report. Additional exploratory analyses of the data may be conducted as deemed appropriate. Missing data will not contribute to the objectives unless specified otherwise within the analysis methods.

The study has been powered for the primary objective (see sample size determination for the primary objective below), to be evaluated after 12 months of follow-up have been completed. The primary objective data and applicable ancillary objective data will be provided in a 12 month report. Secondary objective data, and remaining ancillary objective data will be provided in a final report. Ancillary objects are considered exploratory. There will be no correction for multiple testing. Intent-to-treat principles will be used for analyzing each of objectives unless otherwise specified.

12.1. Primary Objective

The primary objective is to compare the incidence rate of AF through 12 months between the continuous monitoring arm vs control arm in subjects with a recent ischemic stroke of presumed known origin.

i. Hypothesis

H_0: $h_C(t) = h_T(t)$ for $t \leq 12$ months

- H_A: $h_C(t) \neq h_T(t)$ for $t \leq 12$ months

- where $h_T(t)$ and $h_C(t)$ are the hazard functions of first detected and adjudicated AF at time t for subjects with and without the Reveal LINQ diagnostics for AF, respectively.

ii. Endpoint Definition

AF will be defined as an AF event lasting more than 30 seconds. The first AF episode detected and adjudicated by the endpoint adjudication committee will be used for this analysis.

iii. Performance Requirements

The null hypothesis will be rejected if the two-sided log-rank test p-value is less than 0.05.

iv. Rationale for Performance Criteria

The exact rate of AF is unknown in this population. Data from the CRYSTAL AF study and Rabinstein et al (2013) has been used to estimate the 12 month incidence rate of AF in patients with an ischemic stroke of presumed known origin.
The incidence rate of AF through 12-months was 12.4% in the continuous monitoring arm in the CRYSTAL AF study which was conducted in patients with a cryptogenic stroke.

Rabinstein et al (2013) used prolonged ambulatory cardiac monitoring to assess the incidence rate of paroxysmal AF through 3 weeks in patients with a cryptogenic stroke and patient with a stroke of presumed known cause. The incidence rate of paroxysmal AF through 3-weeks was 25% in the patients with a cryptogenic stroke and 14% in patients with an ischemic stroke of presumed known origin.

The above incidence rates were used to estimate the 12 month incidence rate of AF in patients with ischemic stroke of presumed known origin. We expect the AF incidence rate to be lower in patients with an ischemic stroke of presumed known origin when compared to the incidence rate in patients with a cryptogenic stroke. Consequently, an AF incidence rate of 8% through 12 months is expected in our continuous monitoring arm. An AF incidence rate of 2% through 12 months is expected our control arm. This is the same rate observed in the control arm for the CRYSTAL AF study. Although we expect the AF incidence rate to be lower in patients with an ischemic stroke of presumed known origin, we also expect that present standard of care treatment includes more rhythm monitoring then it did during the time of the CRYSTAL AF study.

The objective of the study is to compare the incidence rate of AF through 12 months between the continuous monitoring arm vs control arm in subjects with a recent ischemic stroke of presumed known origin.

v. Sample Size Determination

- A total of 23 events are required to demonstrate that the hazard rates are different between the two arms. Approximately 496 subjects will be enrolled. Assuming 2% attrition from enrollment to randomization, approximately 486 subjects will randomized. Subjects will be followed for 3 years, until end of device life or until official study closure, whichever occurs first. Withdrawn subjects will not be replaced.

- The assumptions used for the calculations were:

  - Two-sided log-rank test
  - Alpha = 0.05
  - 85% power
  - Annual Attrition rate = 10%
  - Annual Crossover rate = 5%
  - Follow-up Time = 1 year
  - Event-free one-year survival rate of 92% in the continuous monitoring arm
  - Event-free one-year survival rate of 98% in the control arm (hazard ratio of 4.13)

The procedure "Logrank tests (Lakatos) using proportion surviving" in PASS 2008 was used to calculate the number of events required to test this objective.

vi. Analysis Methods

Arrhythmic episodes recorded by the subject's inserted/implanted device or from external monitoring will be adjudicated by the adjudication committee to determine if they meet the primary endpoint definition. Only first detected and adjudicated AF episodes within 12-months (365 days) will be included for analysis. Each subject's time to first detected and adjudicated AF will be defined as the time from randomization date to the date of first documented AF (i.e. the date of event per Reveal LINQ ICM, ECG, etc) to meet the primary endpoint definition, and Kaplan-Meier curves will be generated for each randomization arm. Subjects who have not experienced an AF endpoint through 12 months will be censored at:

- their point of last contact, usable Reveal LINQ ICM interrogation or CareLink transmission if they occur before 12 months, whichever is later or

- 12 months if their point of last contact, usable Reveal LINQ ICM interrogation or CareLink transmission go beyond 12 months.

A two-sided log-rank test will be performed to compare the rates between arms.

In addition to the analysis on the full population, subgroup analysis for subjects with small vessel disease stroke and large vessel cervical or intracranial atherosclerosis stroke will be presented.

vii. Determination of Patients/Data for Analysis

- All randomized subjects will be included in the analysis.

12.2. Secondary Objective

To compare the incidence rates of AF through the duration of study follow-up between study arms

i. Hypothesis

H_0: $h_C(t) = h_T(t)$ for $t > 0$ months

- H_A: $h_C(t) \neq h_T(t)$ for $t > 0$ months

- where $h_T(t)$ and $h_C(t)$ are the hazard functions of first detected and adjudicated AF at time t for subjects with and without the Reveal LINQ diagnostics for AF, respectively.

ii. Endpoint Definition

Adjudicated AF event as described in section 13.1.ii.

iii. Performance Requirements

This secondary objective will be tested in a hierarchical fashion. This means that if the primary objective is met, then this secondary objective will be tested.

The null hypothesis will be rejected if the two-sided log-rank test p-value is less than 0.05.

iv. Sample Size Determination

- A two-sided log-rank test with the assumptions below achieves 100% power to test the secondary objective.

- The assumptions used for the calculations were:
  - Two-sided log-rank test
  - Alpha = 0.05
  - Annual Attrition rate = 10%
  - Annual Crossover rate = 5%
  - Accrual Time = 2 years
  - Follow-up Time = 3 years
  - Event-free survival rate of 80% in the continuous monitoring arm
  - Event-free survival rate of 97% in the control arm (hazard ratio of 7.33)
  - Number of subjects randomized = 486

The procedure “Logrank tests (Lakatos) using proportion surviving” in PASS 2008 was used to calculate the power to test this objective.

v. Analysis Methods

Arrhythmic episodes as described in section 13.1 will be used for this objective. However, only first detected and adjudicated AF episodes through the duration of the study follow-up will be included for analysis.

Each subject's time to first detected and adjudicated AF will be defined as in section 13.1, and Kaplan-Meier curves will be generated for each randomization arm. Subjects who have not experienced an AF endpoint through the duration of the study follow-up will be censored at their point of last contact, usable Reveal LINQ ICM interrogation or CareLink transmission, whichever is later. A two-sided log-rank test will be performed to compare the rates between arms.

In addition to the analysis on the full population, subgroup analysis for subjects with small vessel disease stroke and large vessel cervical or intracranial atherosclerosis stroke will be presented.

vi. Determination of Patients/Data for Analysis

All randomized subjects will be included in the analysis.

13. Ethics

13.1. Statement(s) of Compliance

This study will be conducted in compliance with international ethical and scientific quality standards, known as good clinical practice (GCP). GCP includes review and approval by an independent Ethics Board/IRB/MEC before initiating a study, continuing review of an ongoing study by an Ethics Boards and obtaining and documenting the freely given informed consent of a subject before initiating the study. The principles of the Declaration of Helsinki have been implemented through the patient informed consent (IC) process, Ethics Board/IRB/MEC approval, study training, clinical trial registration, preclinical testing, risk-benefit assessment and publication policy.

This study will be conducted in compliance with 21 CFR Parts 11, 50 and 56.

The study will be publicly registered prior to first enrollment in accordance with the 2007 Food and Drug Administration Amendments Act (FDAAA) and Declaration of Helsinki on http://clinicaltrials.gov (PL 110-85, Section 810(a)).

Approval of the CIP is required from the following groups prior to any study procedures at a study site:

- Medtronic
- An independent medical ethics committee or institutional review board.

14. Study Administration

14.1. Monitoring

It is the responsibility of Medtronic to ensure proper monitoring of this clinical study. Trained Medtronic personnel or delegates appointed by Medtronic may perform study monitoring at the study site in order to ensure that the study is conducted in accordance with the CIP, the Clinical Trial Agreement, and applicable regulatory and local requirements. Medtronic, or delegates, must therefore be allowed access to the subjects' case histories (clinic and hospital records, and other source data/documentation) upon request as per the Subject Informed Consent, Research Authorization (where applicable) and Clinical Trial Agreement. The principal investigator should also be available during monitoring visits.

14.1.1. Monitoring Visits

Frequency of monitoring visits may be based upon subject enrollment, duration of the study, study compliance, number of adverse events, number of deviations, findings from previous monitoring visits and any suspected inconsistency in data that requires investigation. Regulatory documents may be reviewed at each study site. Monitoring for the study, including site qualification visits, site initiation visits, interim monitoring visits, and closeout visits, will be done in accordance to the study-specific monitoring plan. Monitoring will be planned either at the study site, via telephone, or electronically to assure compliance with the study protocol. Site activation, periodic visits and study closure visits will occur either on-site, via email or via telephone.

Monitoring visits may be conducted periodically to assess site study progress, the investigator's adherence to the CIP, regulatory compliance including but not limited to IRB/Ethics Committee approval and review of the study, maintenance of records and reports, and review of source documents against subject e-CRFs. Monitors review site regulatory and study compliance by identifying findings of non-compliance and communicating those findings along with recommendations for preventative/corrective actions to site personnel. Monitors may work with study personnel to determine appropriate corrective action recommendations and to identify trends within the study or at a particular site.

14.2. Data Management

Data will be collected using an electronic data management system for clinical studies. e-CRF data will be stored in a secure, password-protected database which will be backed up nightly. Data will be reviewed using programmed and manual data checks. Data queries will be made available to sites for resolution. Study management reports may be generated to assess data quality and study progress. At the end of the study, the data will be frozen and will be retained indefinitely by Medtronic.

All records and other information about subjects participating in this study will be treated as confidential. Data will be transferred and processed by Medtronic or a third party designated by Medtronic in a key coded form, unless it's impossible to make it anonymous, for instance, where the patient's name cannot be removed from the data carrier, such as fluoroscopy images.

Procedures in the CIP require source documentation. Source documentation will be maintained at the site. Source documents, which may include worksheets, patient medical records, programmer printouts, and interrogation files, must be created and maintained by the investigational site team.

The data reported on the e-CRFs shall be derived from source documents and be consistent with these source documents, and any discrepancies shall be explained in writing.

Device data from Reveal LINQ ICM interrogations and CareLink transmissions will be uploaded to secure servers. Save-to-media data collected at office visits will be sent to Medtronic. Upon receipt, device data will be maintained with databases and retrieved for analysis and reporting.

14.3. Direct Access to Source Data/Documents

The sponsor or a regulatory authority may audit or inspect the study site to evaluate the conduct of the study. The clinical investigator(s)/institution(s) shall allow study related monitoring, audits, Ethics Board review and regulatory inspection by providing direct access to source data/documents.

14.4. Confidentiality

All records and other information about subjects participating in this study will be treated as confidential. The identity of a subject will never be disclosed in the event that study data are published.

14.5. Liability

Warranty information is provided in the product packaging for the Reveal LINQ ICM and additional copies are available upon request.

14.6. CIP Amendments

Approval of the CIP is required from the following groups prior to any study procedures at a study site:

- Medtronic
- An independent medical ethics committee or institutional review board.

Similarly, approval of subsequent revisions to the CIP is required at each study site from the above-mentioned groups prior to implementation of the revised CIP at that site.

14.7. Record Retention

14.7.1. Investigator Records

The investigator is responsible for the preparation and retention of the records cited below. All of the below records, with the exception of case history records and case report forms, should be kept in the Investigator Site File (i.e., the study binder provided to the investigator) or Subject Study Binder. E-CRFs must be maintained and signed electronically within the electronic data capture system during the study. The following records are subject to inspection and must be retained for a period of two years (or longer as local law or hospital administration requires) after the date on which the investigation is terminated.

- All correspondence between the IRB/MEC, sponsor, monitor, and the investigator that pertains to the investigation, including required reports.
- Subject's case history records, including:
    - Signed and dated informed consent form personally signed by subject
    - Observations of adverse events/adverse device effects/device deficiencies
    - Medical history
    - Insertion and follow-up data
    - Documentation of the dates and rationale for any deviation from the protocol
- List of investigation sites
- All approved versions of the CIP, and PIC.
- Signed and dated Clinical Trial Agreement.
- Current curriculum vitae of principal investigators.
- Documentation of delegated tasks.
- IRB/MEC approval documentation. Written information that the investigator or other study staff, when member of the IRB/MEC, did not participate in the approval process. Approval documentation must include the Ethics Board composition, where required per local law.
- Study training records for site staff.
- Final Study Report including the statistical analysis.

14.7.2. Investigator Reports

The investigator is responsible for the preparation (review and signature) and submission to the sponsor of all case report forms, adverse events and adverse device effects (reported per the country-specific collection requirements), device deficiencies, deaths, and any deviations from the clinical investigation plan. If any action is taken by an IRB/MEC with respect to this clinical study, copies of all pertinent

documentation must be forwarded to Medtronic in a timely manner. Reports are subject to inspection and to the retention requirements as described above for investigator records.

Safety data investigator reporting requirements are listed in Section 10 of the Adverse Event section. The investigator shall prepare and submit in a complete, accurate and timely manner the reports listed in this section.

Table 10: Investigator Reports per Medtronic Requirements

Report	Submit to	Description/Constraints
Withdrawal of IRB/MEC approval	Sponsor and Relevant Authorities	The investigator must report a withdrawal of approval by the reviewing IRB/MEC of the investigator's part of the investigation within 5 working days.
Study Deviations	Sponsor and IRB/MEC	Any deviation from the clinical investigational plan shall be recorded together with the explanation of the deviation. Notice of deviations from the CIP to protect the life or physical well-being of a subject in an emergency shall be given as soon as possible, but no later than 5 working days after the emergency occurred. Except in such emergency, prior approval is required for changes in the plan or deviations.
Final Report	IRBs/MECs and Relevant Authorities	This report must be submitted within 3 months of study completion or termination.

14.7.3. Sponsor Records

Medtronic shall maintain the following accurate, complete, and current records:

- All correspondence which pertains to the investigation
- Signed Investigator Trial Agreements and current curriculum vitae of principal investigator and delegated task list
- All approved informed consent templates, and other information provided to the subjects and advertisements, including translations
- Copies of all IRB/MEC approval letters and relevant IRB/MEC correspondence and IRB/MEC voting list/roster/letter of assurance
- Names of the institutions in which the clinical study will be conducted
- Names/contact addresses of monitors
- Statistical analyses and underlying supporting data
- Final report of the clinical study
- The Clinical Investigation Plan
- Study training records for site personnel and Medtronic personnel involved in the study
- Any other records that local regulatory agencies require to be maintained.

14.7.4. Sponsor Reports

Medtronic shall prepare and submit the following complete, accurate, and timely reports listed in the table below. In addition to the reports listed below, Medtronic shall, upon request of reviewing IRB/MEC, regulatory agency or FDA, provide accurate, complete and current information about any aspect of the investigation. Safety data Medtronic reporting requirements are listed in Section 10 of the Adverse Event section.

Table 11: Sponsor Reports

Report	Submit to	Description/Constraints
Premature termination or suspension of the clinical investigation	Investigators<br>IRB/MEC<br>Relevant authorities<br>Head of the Institution	Provide prompt notification of termination or suspension and reason(s).
Final report	Investigators,<br>IRB/MEC,	A final report will be submitted to the investigators, and IRBs/MECs within six months after completion or termination of this study.
Study deviation	Investigators	Site specific study deviations will be submitted to investigators periodically.

Medtronic records and reports will be maintained in a password-protected document management system, and paper documents (where applicable) will be stored in stored in secured file cabinets at Medtronic during the course of this study.

After closure of the study Medtronic will archive records and reports indefinitely.

14.8. Publication and Use of Information

Publications from the Stroke AF study will be handled according to Cardiac Rhythm Heart Failure Standard Operating Procedures and as indicated in the Clinical Trial Agreement.

14.8.1. Publication Committee

Medtronic may form the Stroke AF Publication Committee from study investigators. Medtronic personnel may serve as members of the committee. This committee will manage study publications with the goal of publishing findings from the data. The Publication Committee will develop the final Publication Plan as a separate document.

The Publication Committee's role is to: 1) manage elements addressed in the publication plan as outlined in this appendix, 2) develop the final Publication Plan under separate cover, 3) execute the Publication Plan, 4) oversee the publication of primary and ancillary study results, 5) review and prioritize publication proposals, 6) provide input on publication content, and 7) determine authorship.

In addition, the committee will apply and reinforce the authorship guidelines set forth in the Publication Plan.

Membership in the Publication Committee does not guarantee authorship. The committee will meet at regular intervals, as needed.

14.8.2. Management of Primary, Secondary and Ancillary Publications

The Publication Committee reviews, prioritizes, and manages all publications including primary, secondary and ancillary publications. Primary and secondary publications are those that address analyses of any or all primary objectives or secondary objectives, respectively, as specified in the Clinical Investigation Plan.

An ancillary publication is any publication that does not address the study objectives identified in the Clinical Investigation Plan. They include publications proposed and developed by other Medtronic departments or entities, clinicians participating in this clinical study, and clinicians not participating in this clinical study. The committee will work with Medtronic to ensure that requests do not present conflicts with other proposals, are not duplicative, and to determine which ancillary publication proposals, if any, will be supported.

The committee may decide that no publications, including abstracts, will be published prior to the end of the study or with individual site data. Requests for publications on study objectives utilizing subset data (e.g., regional) will be evaluated for scientific validity and the ability of Medtronic to provide resources.

14.8.3. Criteria for Determining Authorship

Publications will adhere to authorship criteria defined by the International Committee of Medical Journal Editors (ICMJE, Uniform requirements for manuscripts submitted to biomedical journals, www.icmje.org). Individual authorship criteria defined by the target journal or conference will be followed when it differs from ICMJE criteria.

Authors, including Medtronic personnel, must at a minimum meet all of the conditions below:

- Substantial contribution to conception and design, or acquisition of data, or analysis and interpretation of data
- Drafting the article or revising it critically for important intellectual content
- Final approval of the version to be published

Decisions regarding authorship and contributor-ship will be made by the committee. The selected authors will be responsible for drafting the publication. All selected authors must fulfill the authorship conditions stated above to be listed as authors, and all contributors who fulfill the conditions must be listed as authors.

All investigators not listed as co-authors will be acknowledged as the "Medtronic Stroke AF Clinical Study Investigators" and will be individually listed according to the guidelines of the applicable scientific journal when possible and affiliation. Any other contributors will be acknowledged by name with their specific contribution indicated.

14.8.4. Transparency

Transparency of study results will be maintained by the following means:

- a final report, describing the results of all objectives and analysis, will be distributed to all investigators, MECs and Competent Authorities of participating countries when required by local law
- registering and posting the study results on ClinicalTrials.gov based on the posting rules stipulated
- submitting for publication the primary study results after the study ends
- disclosing conflicts of interest of the co-authors of publications according to the policies set forth by the corresponding journals and conferences
- making an individual sites study data accessible to the corresponding investigator after the completion of the study, if requested

14.9. Suspension or Early Termination

14.9.1. Planned Study Closure

Study Closure is a process initiated by distribution of a study closure letter. Study closure is defined as closure of a clinical study that occurs when Medtronic and/or regulatory requirements have been satisfied per the Clinical Investigation Plan and/or by a decision by Medtronic or regulatory authority), whichever occurs first. The study closure process is complete upon distribution of the Final Report or after final payments, whichever occurs last. Ongoing Ethics Committee oversight is required until the overall study closure process is complete. Refer to section 7.14 for additional information regarding study exit procedures.

14.9.2. Early Termination or Suspension

Early Termination of the Study is the closure of a clinical study that occurs prior to meeting defined endpoints. This is possible for the whole study or a single site. Study Suspension is a temporary postponement of study activities related to enrollment and distribution of the product. This is possible for the whole study or a single site.

14.9.2.1. Study-wide Termination or Suspension

Possible reasons for considering study suspension or termination of the study include but are not limited to:

- Adverse events associated with the system or product under investigation which might endanger the safety or welfare of the subject
- Observed/suspected performance different from the product's design intent
- Decision by Medtronic or regulatory body (where the study is operating under regulatory body authority)
- Technical issues during the manufacturing process

14.9.2.2. Investigator/Site Termination or Suspension

Possible reasons for clinical investigator or site termination or suspension include but are not limited to:

- Failure to obtain initial Ethics Committee approval or annual renewal of the study
- Persistent non-compliance to the clinical investigation (e.g. failure to adhere to inclusion/exclusion criteria, failure to follow subjects per scheduled follow-ups)
- Lack of enrollment
- Noncompliance to regulations and the terms of the Clinical Trial Agreement (e.g. failure to submit data in a timely manner, failure to follow-up on data queries and monitoring findings in a timely manner, etc.)
- Ethics Committee suspension of the site
- Fraud or fraudulent misconduct is discovered (as defined by local law and regulations)
- Investigator request (e.g. no longer able to support the study)

14.9.3. Procedures for Termination or Suspension

14.9.3.1. Medtronic-Initiated and Regulatory Authority-Initiated

- Medtronic will promptly inform the clinical investigators of the termination or suspension and the reasons and inform the regulatory authority(ies) where required
- In the case of study termination or suspension for reasons other than a temporary MEC/IRB/Head of Medical Institution approval lapse, the investigator will promptly inform the MEC/IRB/Head of Medical Institution
- In the case of study termination, the investigator must inform the subjects and may inform the personal physician of the subjects to ensure appropriate care and follow-up is provided
- In the case of a study suspension, subject enrollment must stop until the suspension is lifted by Medtronic
- In the case of a study suspension, enrolled subjects should continue to be followed out of consideration of their safety, rights and welfare

14.9.3.2. Investigator-Initiated

- The investigator will inform Medtronic and provide a detailed written explanation of the termination or suspension
- The investigator will promptly inform the institution (where required per regulatory requirements)
- The investigator will promptly inform the MEC/IRB/Head of Medical Institution
- The investigator will promptly inform the subjects and/or the personal physician of the subjects to ensure appropriate care and follow-up is provided
- In the case of a study suspension, subjects enrolled should continue to be followed out of consideration of their safety, rights and welfare

14.9.3.3. Ethics Committee-Initiated

- The investigator will inform Medtronic and provide a detailed written explanation of the termination or suspension within 5 business days
- Subject enrollment must stop until the suspension is lifted
- Subjects already enrolled should continue to be followed in accordance with MEC/IRB/Head of Medical Institution policy or its determination that an overriding safety concern or ethical issue is involved
- The investigator will inform his/her institution (where required per local requirements)
- The investigator will promptly inform the subjects, or legally-authorized designees or guardians and/or the personal physician of the subjects, with the rationale for the study termination or suspension

15. References

Alejandro A. Rabinstein, M. J. (2013). Paroxysmal Atrial Fibrillation in Cryptogenic Stroke:A Case–Control Study. *Journal of Stroke and Cerbrovascular Disease*, 1-7.

Dariush Mozaffarian, E. J.-P. (2015). Heart Disease and Stroke Statistics—2015 Update: A Report From the American Heart Association . *Circulation*, 29-322.

L. M. Christensen, D. W. (2014). Paroxysmal atrial fibrillation occurs often in cryptogenic. *European Journal of Neurology*, 884-889.

Luciano A. Sposato, M. M. (2012). Newly Diagnosed Atrial Fibrillation after Acute Ischemic Stroke and Transient Ischemic Attack: Importance of Immediate and Prolonged Continuous Cardiac Monitoring. *Journal of Stroke and Cerebrovascular Diseases*, 210-216.

Owen, A. (2009). Antithrombotic treatment for the primary prevention of stroke in patients with non valvular atrial fibrillation: A reappraisal of the evidence and network meta analysis. *International Journal of Cardiology*, 1-6.

Paul D. Ziegler, M., Taya V. Glotzer, M., Emile G. Daoud, M., D. George Wyse, M. P., Daniel E. Singer, M., Michael D. Ezekowitz, M. P., et al. (2010). Incidence of Newly Detected Atrial Arrhythmias via Implantable Devices in Patients With a History of Thromboembolic Events. *Stroke, Journal of the American Heart Association*, 256-260.

Paul D. Ziegler, T. V. (2009). Incidence of Newly Detected Atrial Arrhythmias via Implantable Devices in Patients With a History of Thromboembolic Events. *Stroke: Journal of the American Heart Association*, 256-260.

Ralph L. Sacco, M. M., Robert Adams, M. F., Greg Albers, M., Mark J. Alberts, M. F., Oscar Benavente, M., Karen Furie, M. M., et al. (2006). Guidelines for Prevention of Stroke in Patients With Ischemic Stroke or Transient Ischemic Attack. *Stroke*, 577-617.

S. Adam Strickberger, M. J. (2005). Relationship between atrial tachyarrhythmias and symptoms. *Heart Rhythm Society*, 125-131.

Tommaso Sanna, M. H.-C. (2014). Cryptogenic Stroke and Underlying Atrial Fibrillation. *The new england journal of medicine*, 2478-2486.

William C. Choe, M. R.-C. (2015). A Comparison of Atrial Fibrillation Monitoring Strategies after Cryptogenic Stroke. *The American Journal of Cardiology*.

16. Version History

Version	Summary of Changes	
1.0	• Not Applicable, New Document’	
2.0	• Table 1. Taryn Randall now monitoring contact at Medtronic. Rationale: Accuracy<br>• Table 2. Clarified that there will be a CRO to manage blood samples. Rationale: Updated study design includes long term blood sample storage which will need to be managed by a CRO which is TBD.<br>• Section 1 (Synopsis). Updated to reflect changes in study design which include: randomization (SoC vs LINQ), blood draw, additional study sites (was 30 now 40) and larger sample size (was 250, now 496). Study patients to be followed for 3 years, until end of device life or study closure, whichever comes first. Rationale: Medtronic business decision to make the study more robust.<br>• Section 2 (Introduction). Study purpose and description updated to include randomized design, larger sample size(sites and patients) and longer study duration. Rationale: Medtronic business decision to make the study more robust.<br>• Section 6 (Methodology). Study objectives updated based on changes in study design (randomization). Rationale: Medtronic business decision to make the study more robust.<br>• Section 7 (Study Procedures). Added randomization, blood draw, and cross over to study procedure table and updated visit descriptions as applicable. Added clarity around data collection only applicable to one study arm. Randomization section (7.8) added. Rationale: Medtronic business decision to make the study more robust.<br>• Section 9 (Study Deviations). Added crossover as an example of a study deviation. Rationale: Clarity.<br>• Section 13 (Statistical Methods and Data Analysis). Section updated to reflect revised study design and objectives. Rationale: Changes to study design.<br>• Appendix I. Clarified that the CEC could serve as the EAC if desired. Rationale: To allow flexibility in study management.	
3.0	• Glossary added, and document format updated. Rationale: Align with current document template.<br>• Removed contact information. Rationale: Contact information is maintained under separate cover.<br>• Study Description and Statistical Methods and Data Analysis: Removed stopping rule for enrollment based on number of AF events. Rationale: Robust study with adequate data for analysis of all objectives.	

	• Duration: Enrollment updated from 20 to 42 months and study from 4.5 to 6.5 years. Rationale: Estimate updated per observed enrollment rate.<br>• Informed Consent: Updated best practice documentation to should document the informed consent process in the source documentation. Rationale: Clarity.<br>• Study Procedures related to blood draw: Added "or if the patient declines". Rationale: Clarity.<br>• Study Procedures related to Reveal LINQ ICM removed requirements for continuous monitoring arm only. Rationale: Allow for consistency in data collection for subjects with an ICM.<br>• Follow-up Visit Procedure: Removed "in-clinic". Data may be collected remotely for subjects unable to return to the clinic for the follow up visit. Rationale: Reduce patient burden of attending in-clinic as all data may be captured remotely.<br>• System Modification Procedure: Removed "continuous monitoring arm only". Rationale: Device modification should be reported for any subject with an ICM.<br>• Data Review Committee: Steering Committee referenced in committee section. Removed contact details. Rationale: Clarity as contact details for all committees noted to be maintained separately.<br>• Data Review Committee: Clinic Clinical Events Committee (CEC) review. "All" removed regarding reported arrhythmias. Rationale: Consistency with adjudication of time to first event.<br>• Statistical Methods and Data Analysis. Add endpoint AF related hospitalization. Rationale: Business decision for additional analysis.<br>• Statistical Methods and Data Analysis. CareLink transmission may be used for censoring. Rationale: Clarity.<br>• Confidentiality: Added statement that all records and other information about subjects participating in this study will be treated as confidential. The identity of a subject will never be disclosed in the event that study data are published. Rationale: Clarity.	
4.0	• Study Design: Updated the maximum of 200 subjects with lacunar stroke to a maximum of 50% (approximately 248) subjects. Rationale: Allow equal characterization of ischemic population.<br>• Procedure table and follow up: Updated device data to include either interrogation or transmissions. Rationale: Include either mode of device data collection for patients completing visits in-clinic or remotely, consistency with text as updated in CIP revision 3.0.	

Medtronic

Statistical Analysis Plan

**Clinical Investigation Plan Title**	Stroke AF
**Clinical Investigation Plan Version**	Version 2 (27/JAN/2016)
**Sponsor/Local Sponsor**	Medtronic, Inc.<br>8200 Coral Sea Street NE<br>Mounds View, MN U.S.A. 55112
**Document Version**	Version 2.0

Confidentiality Statement

The information contained in this document is confidential and the proprietary property of Medtronic. Any distribution, copying, or disclosure without the prior written authorization of Medtronic is strictly prohibited. Persons to whom the information is disclosed must know that it is confidential and that it may not be further disclosed by them.

This document is electronically controlled. Printed copies are considered uncontrolled.

Table of Contents

1. Version History ... 3
2. List of Abbreviations and Definitions of Terms ... 3
3. Introduction ... 5
4. Study Objectives ... 5
5. Investigation Plan ... 6
6. Determination of Sample Size ... 8
7. Statistical Methods ... 9
7.1 Study Subjects ... 9
7.1.1. Disposition of Subjects ... 9
7.1.2. Clinical Investigation Plan (CIP) Deviations ... 10
7.1.3. Analysis Sets ... 10
7.2. General Methodology ... 11
7.3. Center Pooling ... 12
7.4. Handling of Missing Data and Dropouts ... 12
7.5. Adjustments for Multiple Comparisons ... 12
7.6. Demographic and Other Baseline Characteristics ... 12
7.7. Treatment Characteristics ... 12
7.8. Subgroup Analyses ... 12
7.9. Evaluation of Objectives ... 13
7.10. Safety Evaluation ... 16
7.11. Health Outcomes Analyses ... 17
7.12. Changes to Planned Analysis ... 17
8. Validation Requirements ... 17

1. Version History

Version	Summary of Changes	Author(s)/Title
**1.0**	Not Applicable, New Document	Francesca Lemme, Sr. Statistician<br>Brett Peterson, Sr. Statistician
**2.0**	• Added description of events required to demonstrate the primary objective of the study in section 6.1<br>• Clarified that mentioned attrition and crossover rates are for each treatment group in section 6.2	Francesca Lemme, Sr. Statistician<br>Carola Alfaro Vives, Pr. Statistician

2. List of Abbreviations and Definitions of Terms

1798

**Abbreviation**	**Definition**
AE	Adverse Event
AF	Atrial Fibrillation
AT	Atrial Tachycardia
CABG	Coronary Artery Bypass Grafting
CEC	Clinical Events Committee
CIP	Clinical Investigation Plan
CRF	Case Report Form (synonymous with eCRF)
CRT	Cardiac Resynchronization Therapy
CT	Computerized Tomography
ECG	Electrocardiogram
eCRF	electronic Case Report Forms (synonymous with CRF)
ICD	Implantable Cardioverter Defibrillator
ICM	Insertable Cardiac Monitor
IS	Ischemic Stroke
ITT	Intention To Treat
LA	Left Atrial
LAA	Left Atrial Appendage
LVEF	Left Ventricular Ejection Fraction
MRI	Magnetic Resonance Imaging
NIH	National Institutes of Health
OAC	Oral anticoagulation
PP	Per Protocol

RDC	Remote Data Capture
SAP	Statistical Analysis Plan
SOC	System Organ Class
TIA	Transient Ischemic Attack

3. Introduction

The purpose of the Stroke AF study is to compare the incidence of atrial fibrillation (AF) through 12 months by continuous cardiac rhythm monitoring with the Reveal LINQ™ Insertable Cardiac Monitor (ICM) (continuous monitoring arm) vs standard of care (SoC) medical treatment (control arm) in subjects with a recent ischemic stroke of presumed known origin. AF will be defined as an AF event lasting more than 30 seconds and adjudicated by the endpoint adjudication committee.

The study is conducted at approximately 40 sites located in the United States (U.S.) and approximately 496 subjects are being enrolled in the study. A total of 23 first documented AF events within 12 months of randomization are required for the study to test the primary objective. In addition, a guidance was sent to sites stating that a maximum of 3 subjects with lacunar stroke (i.e. small vessel disease) may be enrolled at each site. This restriction is not in the CIP.

The enrollment period started in April 2016 and will take approximately 20 months. Subjects will be followed for 3 years, until end of device life or until official study closure, whichever occurs first. Therefore, the expected study duration from first enrollment to official study closure (final report) is approximately 4.5 years. The duration of individual subject participation will vary based on timing of site activation and their enrollment; however, participation of an individual subject will be for 3 years, until end of device life or until study closure, whichever occurs first.

This Statistical Analysis Plan (SAP) has been designed to document, before data is analyzed, the rationale for the study design, and the planned analyses that will be included in a 12-month follow-up study report related to the primary objective (primary report), and a final report related to the secondary objective. This SAP does not limit the analysis in reports, and additional analysis of the study data beyond this plan is expected.

The Stroke AF CIP Version 2, dated 27/JAN/2016 was used to create this SAP.

4. Study Objectives

The Stroke AF study will compare the incidence rate of AF through 12 months between continuous monitoring and standard of care in subjects with a recent ischemic stroke of presumed known origin. Subjects will be randomized 1:1 to the continuous monitoring arm or control arm.

4.1. Primary objective

To compare the incidence rate of AF through 12 months between the continuous monitoring arm vs control arm in subjects with a recent ischemic stroke of presumed known origin.

4.2. Secondary objective

To compare the incidence rates of AF through the duration of study follow-up between study arms.

4.3. Primary and final reports

The objectives for the primary report can be analyzed after all enrolled subjects have 1) had their 12-month visit, 2) missed the 12-month visit and the 12-month visit window has closed, or 3) exited prior to the 12-month visit window closing. A data snapshot will be used to analyze data for the primary report since follow-up will continue. Once follow-up is complete and the study data collection and cleaning is finalized, a second data snapshot will be used to analyze data for the final report.

Table 1 shows which objectives will be analyzed for the primary and final reports. All objectives will be included in the final report using the final data snapshot.

Table 1: Objectives analyzed for the primary and final reports

Objective	Primary Report	Final Report
Primary Objective	X	X
Secondary Objective		X

5. Investigation Plan

5.1 Study Design and background information

The Stroke AF study is a prospective, multi-site, randomized, controlled, non-blinded, post-market study, comparing the incidence rate of Atrial Fibrillation (AF) through 12 months between continuous cardiac monitoring and standard of care in subjects with a recent ischemic stroke (IS) of presumed known origin. No interim analyses are planned.

Most patients with IS are treated with anti-platelet (AP) therapy. However, patients with IS who have been diagnosed with AF are normally treated with long-term oral anticoagulation (OAC) as OAC is dramatically more effective at preventing recurrent IS than AP therapy in patients with AF. Therefore, detection of AF after IS crucial, as it results in an important change in the administration of antithrombotic therapy from AP to OAC, thereby reducing the risk of recurrent stroke. The CRYSTAL AF study demonstrated that continuous monitoring via the Reveal LINQ ICM is superior to standard of care for detecting AF after cryptogenic stroke. However, it remains unclear what the long-term rate of AF is in the IS patient population with a presumed known origin. The results of the Stroke AF clinical study will then provide evidence that could impact the treatment and long-term prognosis for patients classified as having an IS of presumed known origin by demonstrating the value of long term monitoring for AF in this patient population.

5.2 Eligibility

The inclusion and exclusion criteria to be enrolled into the study are as follows:

Inclusion criteria

- Subject has had an ischemic stroke believed to be due to small vessel disease, large vessel cervical or intracranial atherosclerosis within the past 10 days
- Subject is willing and able to undergo study requirements and expected to be geographically stable during study follow-up
- Subject is 60 years of age or older, or age 50 to 59 years plus a documented medical history of at least one of the following additional risk factors for stroke:
    - Congestive heart failure
    - Hypertension (Systolic Blood Pressure >140)
    - Diabetes Mellitus
    - Prior Stroke (>90 days ago, other than study qualifying index event)
    - Vascular disease (e.g. coronary artery disease, heart attack, peripheral artery disease and complex aortic plaque)

Exclusion criteria

- Subject has had a cryptogenic stroke
- Subject has had a cardioembolic stroke
- Subject has untreated hyperthyroidism
- Subject has had a recent myocardial infarction <1 month of stroke
- Subject has had a recent cardiac surgery (e.g. coronary artery bypass surgery (CABG)) <1 month of stroke
- Subject has a mechanical heart valve
- Subject has valvular disease requiring immediate surgical intervention
- Subject has documented prior history of atrial fibrillation or atrial flutter
- Subject has permanent indication for oral anticoagulation
- Subject has permanent contraindication to oral anticoagulation such that detection of AF would not change medical management, based on enrolling investigators judgment
- Subject is enrolled in a concurrent study that may confound the results of this study. Co-enrollment in any concurrent clinical study (including registries) requires approval of the study manager or designee.
- Subject’s life expectancy is less than 1 year
- Subject is pregnant
- Subject has or is indicated for implant with a pacemaker, ICD, CRT, or an implantable hemodynamic monitor
- Subject with a medical condition that precludes the patient from participation in the opinion of the investigator

5.3. Treatment and Study Procedures

5.3.1. Treatment

Subjects are randomized 1:1 to the continuous monitoring arm or the control arm. Subjects randomized to the continuous monitoring arm will have a Reveal LINQ ICM inserted within 10 days of the qualifying stroke and undergo continuous remote monitoring. Subjects randomized to the control arm will be followed per site specific standard of care.

5.3.2. Study Procedures

Data will be collected via electronic Case Report Forms (eCRF), Reveal LINQ ICM device interrogation files and Medtronic CareLink transmissions. A web-based application tool, Remote Data Capture (RDC), will be used for data entry of the eCRF. Clinical data will be collected at baseline, 1, 6 and 12 months post-randomization, and then at recurring 6 months intervals until study exit. Additionally, subjects randomized to the continuous monitoring arm will have data collected at REVEAL LINQ ICM insertion, 3 and 9 months.

6. Determination of Sample Size

6.1. Primary Objective

A total of 23 first events across the two treatment groups are required to demonstrate the primary objective of the study. Assuming 2% attrition from enrollment to randomization, approximately 486 subjects will be randomized. Subjects will be followed for 3 years, until end of device life or until official study closure, whichever occurs first. However, the primary objective of the study, upon which this sample size calculation is based, will be analyzed considering data up to the 12 months follow-up visit for all subjects in the study at the time of the analysis. This sample size calculation then assumes a follow-up time equal to 1 year and an accrual time equal to zero (data from all subjects is analyzed from randomization to the 12th month follow-up visit, regardless of how long they've been in the study at the time of the analysis). Withdrawn subjects will not be replaced.

The assumptions used for the calculations were:

- Two-sided log-rank test
- Alpha = 0.05
- 85% power
- Annual Attrition rate = 10% (in each treatment group)
- Annual Crossover rate = 5% (in each treatment group)
- Follow-up Time = 1 year
- Event-free one-year survival rate of 92% in the continuous monitoring arm
- Event-free one-year survival rate of 98% in the control arm (hazard ratio of 4.13)

The procedure "Logrank tests (Lakatos) using proportion surviving" in PASS 2008 was used to calculate the number of events required to test this objective.

6.2. Secondary Objective

A number of 486 subjects randomized to achieve the primary objective and the assumptions below will also ensure 100% power to test the **secondary objective** of the study.

The assumptions used for the calculations were:

- Two-sided log-rank test
- Alpha = 0.05
- Annual Attrition rate = 10% (in each treatment group)
- Annual Crossover rate = 5% (in each treatment group)
- Accrual Time = 2 years
- Follow-up Time = 3 years
- Event-free survival rate of 80% in the continuous monitoring arm
- Event-free survival rate of 97% in the control arm (hazard ratio of 7.33)

The procedure "Logrank tests (Lakatos) using proportion surviving" in PASS 2008 was used to calculate the power to test this objective.

7. Statistical Methods

7.1 Study Subjects

7.1.1. Disposition of Subjects

A subject is enrolled in the study when he/she signs and dates the Patient Informed Consent. The subject is then eligible for randomization assignment after study enrollment and verification of eligibility criteria. Enrollment, randomization and insertion of the Reveal LINQ ICM must occur within 10 days of the qualifying stroke event. Once a subject is assigned to a study arm, he is considered eligible. A flow chart similar to Figure 1 will be created to describe patient disposition.

Study exits will be summarized according to exit reason. Violation of inclusion and exclusion criteria at baseline will be summarized for all enrolled patients.

The following tables will be considered to summarize patient disposition:

1. Number (%) of patients per center and randomization group
2. Number (%) of patients by visit and randomization group
3. Follow-up time per randomization group

Follow-up time will be determined as the time between date of enrollment and date of study exit or date of last contact with subject if the subject was lost to follow-up.

Figure 1: Patient Disposition Flow Chart

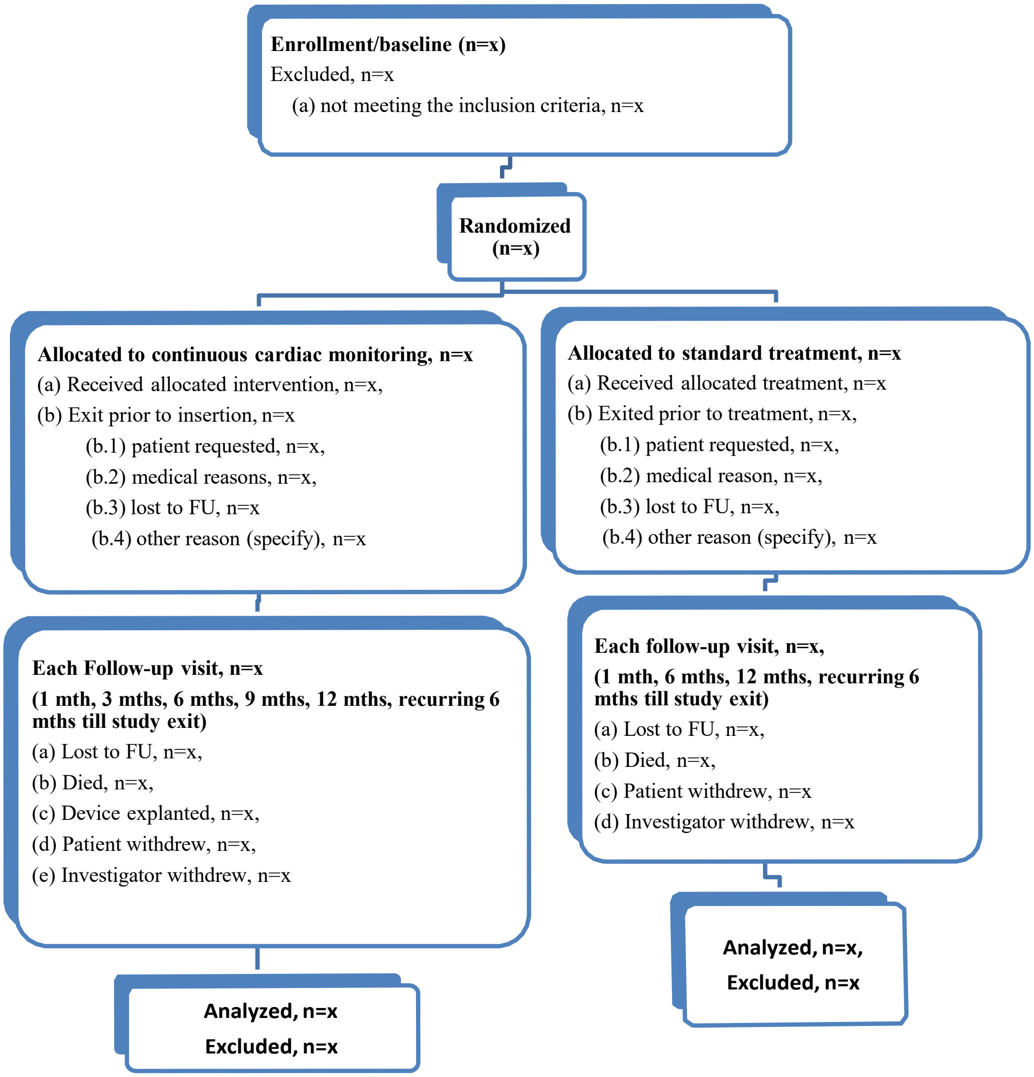

7.1.2. Clinical Investigation Plan (CIP) Deviations

Deviations from the clinical investigation plan will be collected on the Study Deviation eCRF. Deviations will be summarized in the final report in a table by coded category. Deviation coding will be performed by Medtronic, and the coding will be collected on the Medtronic Use Only Deviation eCRF. The number of deviations per category, and the number and percentage of subjects with a deviation in each category will be reported.

7.1.3. Analysis Sets

The following analysis sets will be used in this study:

Enrollment Cohort: All subjects who signed the informed consent and meet inclusion/exclusion criteria.

Intent to Treat (ITT) Cohort: Enrolled subjects who are randomized, according to the treatment arm to which they were randomized.

Per Protocol (PP) Cohort: All subjects who fulfill the protocol as follows:

- In ITT cohort (i.e., meets inclusion/exclusion criteria, gave informed consent, and was randomized)
- Exclude subjects that cross-over to the other arm
- Exclude subjects that are not programmed as follows at any point during the study (from CIP):
  - Reason for Monitoring = Cryptogenic Stroke
  - AF Detection Sensitivity = Balanced Sensitivity
  - Ectopy Rejection = Aggressive
  - AT/AF Recording Threshold = All Episodes
  - Type of AT/AF Detection = AF Only

The ITT Cohort will be used to analyze the primary and secondary objectives. The use of the PP Cohort will be specified in each evaluation objective.

Table 2 below shows the use of each analysis set:

Table 2: Use of analysis cohorts

Analysis Item	Analysis Cohort
All baseline summaries (i.e.: demographic, medical history)	ITT
Primary Objective	ITT, PP
Secondary Objective	ITT, PP
Adverse Event summary	ITT
Device Deficiency summary	ITT
Deviation summary	ITT

Summaries for subjects enrolled but not in the ITT set can be presented if necessary.

7.2. General Methodology

Data summaries for categorical data will be summarized as count, e.g., number of patients, and/or number of events, and a percentage relative to the total number of patients/events. Continuous variables will be represented by mean, standard deviation, median and quartiles.

Time to event methods will be used for the primary and secondary objectives. P-values for hypothesis testing will be evaluated based on two-sided testing using a significance level of 0.05. Confidence intervals will be reported as two-sided 95% confidence intervals.

7.3. Center Pooling

The study is expected to be conducted in approximately 40 centers in the USA. The data from all centers will be pooled. There will be no minimum limit that each investigator must enroll. The maximum number of enrolled subjects per center is 50 subjects. In addition, a guidance was sent to sites stating that a maximum of 3 subjects with lacunar stroke (i.e. small vessel disease) may be enrolled at each site. This restriction is not in the CIP. Adjustment for center effect will not be included in statistical modeling.

7.4. Handling of Missing Data and Dropouts

The primary and secondary objectives use time to event methods, thus there will only be missing data for subjects with no follow-up time. Otherwise, each subject will have data to contribute to the analysis.

7.5. Adjustments for Multiple Comparisons

No adjustments for multiple comparisons will be made.

7.6. Demographic and Other Baseline Characteristics

Baseline variables such as demographics, physical examination, cardiac disease classification, testing results, qualifying stroke event, medications, modified rank scale, and NIH stroke scale information may be reported. Continuous variables will be reported as mean, standard deviation, median, minimum, maximum, and quartiles and categorical variables will be reported as number and percentage of subjects.

The summary tables will include a column for ITT treatment arm subjects, ITT control arm subjects, and all ITT subjects.

A summary table for subjects enrolled but not in the ITT cohort may be presented if necessary.

7.7. Treatment Characteristics

Treatment characteristic variables from the Insertion Procedure and System Modification CRFs will be reported. Continuous variables will be reported as mean, standard deviation, median, minimum, maximum, and quartiles. Categorical variables will be reported as number and percentage of subjects.

The summary tables will include a column for ITT treatment arm subjects who underwent the Reveal implant procedure.

7.8. Subgroup Analyses

Stroke Subgroups

Subgroup analysis for subjects with small vessel disease stroke and large vessel cervical or intracranial atherosclerosis stroke will be presented.

The source data for defining the subgroups come from the question "TOAST Classification of Subtypes of Acute Ischemic Stroke" on the BASELINE CRF.

- If the CRF value is "Large-artery atherosclerosis", then the subject is in the "large vessel cervical or intracranial atherosclerosis" subgroup
- If the CRF value is "Small-vessel occlusion (lacune)", then the subject is in the "small vessel disease stroke" subgroup

Kaplan-Meier curves will be generated for each randomization arm in each subgroup.

A Cox proportional hazards model will be used to test the interaction using the Wald test between treatment and subgroup.

<u>AF Subgroups</u>

Subgroup analysis for subjects with AF and without AF will be presented. AF events adjudicated by the CEC will be used to identify subgroups.

- If a subject has at least one AF event as adjudicated by the CEC during the follow-up period, then the subject is in the "AF" subgroup
- If a subject does not have any AF events as adjudicated by the CEC during the follow-up period, then the subject is in the "No AF" subgroup

The number of events and the number of subjects in each subgroup will be reported overall and within each randomization arm. OAC medications may also be considered in the temporal pattern. A logistic regression model will be used to test the interaction between treatment and subgroup.

<u>Recurrent Stroke Subgroups</u>

Subgroup analysis for subjects with recurrent stroke and without recurrent stroke will be presented. Stroke events adjudicated by the CEC will be used to identify subgroups.

- If a subject has at least one stroke event as adjudicated by the CEC during the follow-up period, then the subject is in the "Stroke" subgroup
- If a subject does not have any stroke events as adjudicated by the CEC during the follow-up period, then the subject is in the "No Stroke" subgroup

The number of events and the number of subjects in each subgroup will be reported overall and within each randomization arm. A logistic regression model will be used to test the interaction between treatment and subgroup.

7.9. Evaluation of Objectives

Primary Objective

The primary objective is to compare the incidence rate of AF through 12 months between the continuous monitoring arm vs control arm in subjects with a recent ischemic stroke of presumed known origin.

i. Hypothesis

H_0: $h(t) = h_T(t)$ for $t \leq 12$ months

H_A: $h_C(t) \neq h_T(t)$ for $t \leq 12$ months

where $h_T(t)$ and $h_C(t)$ are the hazard functions of first detected and adjudicated AF at time t for subjects with and without the Reveal LINQ diagnostics for AF, respectively. Hazard functions and survival functions are transformations of each other.

ii. Endpoint Definition

AF will be defined as an AF event lasting more than 30 seconds. The first AF episode detected and adjudicated by the endpoint adjudication committee will be used for this analysis.

iii. Performance Requirements

The null hypothesis will be rejected if the two-sided log-rank test p-value is less than 0.05.

iv. Analysis Methods

Arrhythmic episodes recorded by the subject's inserted/implanted device or from external monitoring will be adjudicated by the adjudication committee to determine if they meet the primary endpoint definition. Only first detected and adjudicated AF episodes within 12-months (365 days) will be included for analysis. Each subject's time to first detected and adjudicated AF will be defined as the time from randomization date to the date of first documented AF (i.e. the date of event per Reveal LINQ ICM, ECG, etc.) to meet the primary endpoint definition, and Kaplan-Meier curves will be generated for each randomization arm. Subjects who have not experienced an AF endpoint through 12 months will be censored at:

(1) their point of last contact, usable Reveal LINQ ICM interrogation or CareLink transmission if they occur before or at 12 months, whichever is later

or

(2) 12 months if their point of last contact, usable Reveal LINQ ICM interrogation or CareLink transmission go beyond 12 months.

A two-sided log-rank test will be performed to compare the rates between arms. The 12-month survival estimate with its 95% confidence interval will be reported for each arm. The hazard ratio estimate for the treatment effect with its 95% confidence interval will be reported.

The types of monitoring (i.e., wired monitor, wireless monitor, etc.) used to identify AF events in the control arm may be reported to further characterize the events.

v. Determination of Patients/Data for Analysis

The ITT cohort will be used to analyzed this objective. The PP cohort may be used to analyze this objective as a supplementary analysis.

vi. Subgroup Analyses

Subgroup analysis for subjects with small vessel disease stroke and large vessel cervical or intracranial atherosclerosis stroke will be presented. See section 7.9 for details.

Secondary Objective

To compare the incidence rates of AF through the duration of study follow-up between study arms

i. Hypothesis

H_0: $h_C(t) = h_T(t)$ for $t > 0$ months

H_A: $h_C(t) \neq h_T(t)$ for $t > 0$ months

where $h_T(t)$ and $h_C(t)$ are the hazard functions of first detected and adjudicated AF at time t for subjects with and without the Reveal LINQ diagnostics for AF, respectively. Hazard functions and survival functions are transformations of each other.

ii. Endpoint Definition

AF will be defined as an AF event lasting more than 30 seconds. The first AF episode detected and adjudicated by the endpoint adjudication committee will be used for this analysis. This is the same definition used for the primary objective.

iii. Performance Requirements

This secondary objective will be tested in a hierarchical fashion. This means that if the primary objective is met, then this secondary objective will be tested. The null hypothesis will be rejected if the two-sided log-rank test p-value is less than 0.05.

iv. Analysis Methods

Arrhythmic episodes recorded by the subject's inserted/implanted device or from external monitoring will be adjudicated by the adjudication committee to determine if they meet the secondary endpoint definition. Only first detected and adjudicated AF episodes during all follow-up will be included for analysis. Each subject's time to first detected and adjudicated AF will be defined as the time from randomization date to the date of first documented AF (i.e. the date of event per Reveal LINQ ICM, ECG, etc.) to meet the secondary objective endpoint definition.

Each subject's time to first detected and adjudicated AF will be defined as the time from randomization date to the date of first documented AF (i.e. the date of event per Reveal LINQ ICM, ECG, etc.) as in the secondary endpoint definition above, and Kaplan-Meier curves will be generated for each randomization arm. Subjects who have not experienced an AF endpoint through the duration of the study follow-up will be censored at their point of last contact, usable Reveal LINQ ICM interrogation or CareLink transmission, whichever is later.

A two-sided log-rank test will be performed to compare the rates between arms. Survival estimates with 95% confidence intervals will be reported for each arm. The hazard ratio estimate for the treatment effect with its 95% confidence interval will be reported.

The types of monitoring (i.e., wired monitor, wireless monitor, etc.) used to identify AF events in the control arm may be reported to further characterize the events.

v. Determination of Patients/Data for Analysis

The ITT cohort will be used to analyze this objective. The PP cohort may be used to analyze this objective as a supplementary analysis.

vi. Subgroup Analyses

Subgroup analysis for subjects with small vessel disease stroke and large vessel cervical or intracranial atherosclerosis stroke will be presented. See section 7.9 for details.

7.10. Safety Evaluation

Relatedness of adverse events using the CEC adjudication will be summarized using counts and percentages. Seriousness of adverse events using the Medtronic classification will be summarized using counts and percentages. Details of individual adverse events will be listed.

7.11. Health Outcomes Analyses

Health outcomes will be compared between study arms using the EQ-5D questionnaire and symptoms collected in the symptoms assessments.

7.12. Changes to Planned Analysis

Any changes to the data analysis methods described in the CIP and this Statistical Analysis Plan, and the justification for making the change, will be described in the clinical study report. Additional exploratory analyses of the data will be conducted as deemed appropriate.

8. Validation Requirements

Minimum validation requirements for the programs written to execute the analyses in this SAP:

- Primary objective: Level I (independent program)
- Secondary objective: Level II (peer review)
- AE tables and listings: Level II (peer review)
- Deviation table and listing: Level II (peer review)
- Patient disposition flowchart numbers: Level II (peer review)
- Other programs needed for final report analyses not specified: Level II (peer review)

Programs previously validated at Level I or Level II further modified with minor changes may be validated at Level III.

It is expected that Standard Operating Procedures will be followed for other programs that effect the programs written to execute the analyses in this SAP, such as data retrieval programs, dataset mapping programs, analysis dataset programs, etc.
