# Supplementary material for: Atrial Fibrillation In Patients With Stroke Attributed to Large- or Small-Vessel Disease: 3-Year Results From the STROKE AF Randomized Clinical Trial
Source: JAMA Neurol. 2023 Oct 30;80(12):1277–83. doi: 10.1001/jamaneurol.2023.3931 (PMC10616765; doi:10.1001/jamaneurol.2023.3931)
Supplement: Supplement 4. — Data sharing statement [file jamaneurol-e233931-s004.pdf]

Data Sharing Statement

Bernstein. Atrial Fibrillation In Patients With Stroke Attributed to Large- or Small-Vessel Disease. *JAMA Neurol*. Published October 30, 2023. doi:10.1001/jamaneurol.2023.3931

Data
Data available: No
